# Sub‐Micromolar Pulse Dipolar EPR Spectroscopy Reveals Increasing Cu^II^‐labelling of Double‐Histidine Motifs with Lower Temperature

**DOI:** 10.1002/anie.201904848

**Published:** 2019-07-18

**Authors:** Joshua L. Wort, Katrin Ackermann, Angeliki Giannoulis, Alan J. Stewart, David G. Norman, Bela E. Bode

**Affiliations:** ^1^ EaStCHEM School of Chemistry Biomedical Sciences Research Complex, and Centre of Magnetic Resonance University of St Andrews North Haugh St Andrews KY16 9ST UK; ^2^ School of Medicine Biomedical Sciences Research Complex, and Centre of Magnetic Resonance University of St Andrews North Haugh St Andrews KY16 9TF UK; ^3^ School of Life Sciences University of Dundee, Medical Sciences Institute Dundee DD1 5EH UK

**Keywords:** dissociation constant, double-histidine motif, EPR spectroscopy, non-covalent interactions, RIDME

## Abstract

Electron paramagnetic resonance (EPR) distance measurements are making increasingly important contributions to the studies of biomolecules by providing highly accurate geometric constraints. Combining double‐histidine motifs with Cu^II^ spin labels can further increase the precision of distance measurements. It is also useful for proteins containing essential cysteines that can interfere with thiol‐specific labelling. However, the non‐covalent Cu^II^ coordination approach is vulnerable to low binding‐affinity. Herein, dissociation constants (*K*_D_) are investigated directly from the modulation depths of relaxation‐induced dipolar modulation enhancement (RIDME) EPR experiments. This reveals low‐ to sub‐μm Cu^II^ *K*_D_s under EPR distance measurement conditions at cryogenic temperatures. We show the feasibility of exploiting the double‐histidine motif for EPR applications even at sub‐μm protein concentrations in orthogonally labelled Cu^II^–nitroxide systems using a commercial Q‐band EPR instrument.

With the increasing complexity and scope of the biomolecular structures being studied, pulse dipolar electron paramagnetic resonance (PDEPR) spectroscopy is an attractive technique that can complement crystallography, NMR spectroscopy, or cryo electron microscopy with nanometre distance constraints. Similar to Förster resonance energy transfer (FRET), PDEPR does not require crystallisation, is not size‐limited, and is performed in solution. It has been used to investigate the structure and dynamics of proteins and nucleic acids on a length scale of 1.5–16 nm^1^ including multi‐component systems,^2^ intermolecular domain interactions,^3^ contribute distance constraints for structural modelling,^4^ and mechanistic insights.^5^ Furthermore, PDEPR can be used to monitor complexation^6^ and so principally can couple structural information to binding equilibria.^7^ Commonly, pairs of paramagnetic moieties, such as nitroxide radicals, are site‐specifically conjugated with thiol side‐chains of cysteines introduced at strategic positions via site‐directed mutagenesis. Covalent attachment of the commercial methanethiosulfonate spin label (MTSL, Figure 1 a) to cysteines results in the modified amino acid R1 bearing a spin‐labelled side‐chain. A major strength of this methodology is the opportunity to measure distances between identical labels.

**Figure 1:**
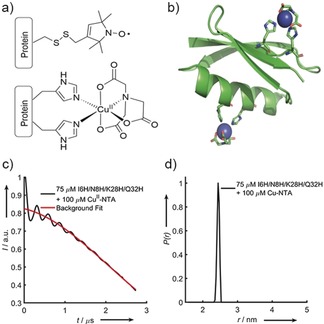
a) The structure of the modified cysteine residue R1 (top) and the Cu^II^–NTA spin label, coordinated to the δ‐nitrogen atoms of the imidazole rings of a protein dH site (bottom). b) Double‐dH (I6H/N8H/K28H/Q32H GB1) construct in cartoon representation (PDB: 4WH4),^14^ with the Cu^II^–NTA spin labels and coordinating dH sites in stick representation and Cu^II^ ions as blue spheres. c) Raw RIDME trace (black) and background fit (red) for the measurement of 75 μm double‐dH protein in the presence of 100 μm Cu^II^–NTA spin label and d) distance distribution corresponding to the raw RIDME trace shown in (c).

Nonetheless, spectroscopically orthogonal spin labels, such as transition metal ions,^8^ lanthanides,^9^ and triarylmethyl‐based spin labels,^10^ have gained increasing attention for use in conjunction with nitroxides. This is appealing because it can expand the accessible information content of a single multi‐labelled sample.^11^ Importantly, most labelling strategies rely on conjugation to cysteine thiols,^12^ which makes orthogonal site‐specific labelling problematic. To overcome this, genetically encoded labels can be used, though as non‐canonical amino‐acids,^13^ which can be more structurally perturbing than the post‐translational modification of natural amino‐acids, and can also restrict the yield of label incorporation.

Recently, Cu^II^‐labelling using genetically encoded double‐histidine (dH) motifs,^14^ introduced in α‐helices (at residue positions *i* and *i+4*), and β‐sheets (at residue positions *i* and *i+2*) have emerged as alternatives to nitroxides for pulse EPR applications.^15^ Significantly, the chemical orthogonality of histidine coordination to covalent cysteine modification brings systems previously unamenable to standard labelling (for example, those containing essential disulfide bridges) into reach. Furthermore, bipedal attachment and a reduced number of rotatable bonds between the paramagnetic centre and the protein backbone reduces the conformational flexibility of dH labels compared to R1, leading to dramatically improved precision in macromolecular distance measurements.^15^ This makes dH–Cu^II^ a powerful tool for the study of subtle conformational changes^16^ that may otherwise be hidden in the intrinsically broad inter‐label distributions observed with common spin labels.

However, as Cu^II^‐labelling of dH sites is non‐covalent, the binding equilibrium is described by a dissociation constant (*K*_D_). A poor dH affinity for Cu^II^ would lead to compromise on two fronts: i) either the efficiency of protein labelling would be consistently low, or ii) the excess of Cu^II^‐label would be very large, leading to a signal dominated by free label. This would diminish the modulation depth (Δ) and make retrieval of the dipolar signal challenging as instrumental artefacts and background become more dominant. Another limitation is the reduced sensitivity of the routinely used pulse electron–electron double resonance (PELDOR)^17^ experiment when applied to paramagnetic metal ions due to their large spectral widths. In this work, the 5‐pulse relaxation‐induced dipolar modulation enhancement (RIDME) experiment^18^ is used to mitigate this sensitivity issue. By refocusing the dipolar interaction using a stochastic spin flip rather than a microwave pulse, the common pulse excitation bandwidth limitation of PDEPR is overcome, leading to significant sensitivity enhancement. In the case of RIDME measurements between nitroxides and metal ions, the detection of the nitroxide spin improves the signal while also allowing for the addition of excess Cu^II^, thereby improving occupation of the dH site without significantly reducing sensitivity, as free Cu^II^ will not contribute to the EPR echo signal. However, the addition of large excesses of Cu^II^ will detriment sensitivity by shortening transverse relaxation and steepening the background. *K_D_*s have previously been estimated for Cu^II^–iminodiacetic acid (IDA) and Cu^II^–nitrilotriacetic acid (NTA) complexes binding to the protein dH sites used in this work.^15^

A group G *Streptococcus* protein G, domain B1 (GB1) tetra‐histidine (double‐dH) construct I6H/N8H/K28H/Q32H15a is investigated by RIDME before the investigation of the individual dH sites through two dH/R1 constructs with dH sites in a β‐sheet (I6H/N8H/K28R1) and an α‐helix (I6R1/K28H/Q32H), respectively. Lastly, we infer a sub‐μm binding affinity from 5‐pulse RIDME experiments at sub‐μm protein concentrations.

The 5‐pulse RIDME experiment (see the Supporting Information for details) on homo or hetero spin‐pairs relies on detected spins (A spins) accumulating a phase factor by longitudinal relaxation (with time constant *T*_1_) of partner spins (B spins) during the mixing‐time interval *T*_mix_. When varying the position of *T*_mix_ in the pulse sequence, the phase factor manifests as a modulation of the detected refocused electron spin echo by the dipolar coupling, ω_ΑΒ_, which is proportional to the inter‐spin distance *r*_AB_^−3^.^19^

For a system consisting of two electron spins *S*=1/2 (such as Cu^II^ or nitroxide), the *T*_mix_‐ and *T*_1_‐dependent modulation depth ($\Delta_{Tmix}$ ) is given by:18b

(1) $$\Delta_{Tmix}\;=\;\frac{1}{2}\left(1-exp\left(\frac{{-T}_{mix}}{T_{1}}\right)\right)$$

For a double‐dH construct, only proteins with both dH sites occupied will contribute to the experimental modulation depth (Δ ). With increasing Cu^II^ concentration $\Delta \times {\Delta_{Tmix}}^{-1}$ will first increase while the dH sites are not saturated; therefore, most detected Cu^II^ will be dH‐bound and contribute to Δ . However, once the dH sites saturate, all additional Cu^II^ will be free in solution and reduce Δ towards 0 as the Cu^II^ concentration tends to infinity (see the Supporting Information).

This is overcome in the orthogonally labelled case, where quantitatively bound nitroxide is detected. Here, $\Delta \times {\Delta_{Tmix}}^{-1}$ reports the loading with Cu^II^, and Δ will tend asymptotically to the theoretical limit of 0.5, as Cu^II^ tends to infinite concentration, since the excess of Cu^II^ does not contribute to the signal. This means that $\Delta \times {\Delta_{Tmix}}^{-1}$ as a function of Cu^II^ concentration is described by a quadratic binding equation:

(2) $$\Delta \times {\Delta_{Tmix}}^{-1}=\;\frac{\left(K_{D}+{\left[P\right]}_{t}+{\left[M\right]}_{t}\right)-\sqrt{{\left(K_{D}+{\left[P\right]}_{t}+{\left[M\right]}_{t}\right)}^{2}-4{\left[P\right]}_{t}{\left[M\right]}_{t}}}{2{\left[P\right]}_{t}}$$

where [*P*]_*t*_ and [*M*]_*t*_ are the concentrations of total protein and metal complex, respectively.

RIDME measurements were performed on the double‐dH GB1 construct (Figure 1 b) at 75 μm protein concentration in presence of 100 μm Cu^II^–NTA (Figure 1 a). The sensitivity is estimated to be a factor of approximately 100 superior to PELDOR (see below). However, estimating *K*_D_ from Cu^II^‐detected PDEPR has several pitfalls. Speciation of Cu^II^ ions into free and bound species leads to different transverse and longitudinal relaxation behaviour and EPR spectra for the different Cu^II^ species. This means the contributions to the signal will depend not only on the stoichiometric factors but also on the spectral position that is detected and the actual dipolar evolution time and experiment repetition rate. In this light, we refrained from quantifying *K*_D_s from Cu^II^‐detected PDEPR. Nevertheless, in favourable cases deviations between species can be negligible or are readily determined from independent experiments.

In an approach to independently investigate the *K*_D_ values of both dH sites via Cu^II^–nitroxide RIDME modulation depths, constructs I6R1/K28H/Q32H and I6H/N8H/K28R1 GB1 were designed (Figure 2 a and b, respectively), produced and characterised (see the Supporting Information). In analogy to previous studies, the binding strength can be estimated from PDEPR modulation depths by adding increasing amounts of titrant.6a For practical reasons pseudo‐titrations (as a titration with discrete samples prepared for each data point in the series) were performed. Initial predictions of concentration ranges consistently under‐estimated the Cu^II^ loading and this led us to incrementally decrease protein and spin label concentrations. Speculation that the change in cryoprotectant and buffer conditions used here (50 mm phosphate + 50 % ethylene glycol in contrast to 50 mm N‐ethyl morpholine + 20 % glycerol used previously)^15^ might cause the increased affinity were disproved (see the Supporting Information). Titrations at 25 and 75 μm protein concentrations led to *K*_D_ estimates of below 3 μm for all four combinations of both spin labels (Cu^II^–IDA and Cu^II^–NTA) with both constructs. The lowest estimate was below 500 nm for I6R1/K28H/Q32H and Cu^II^–NTA. It is important to note that at protein concentrations two orders of magnitude above the estimated *K*_D_, our approach does not allow a precise determination (see the Supporting Information). Thus, approximate Cu^II^‐loading was inferred from RIDME traces measured for both constructs at 5 μm protein and spin‐label concentration, confirming significant loading at low μm concentration with again the highest loading for I6R1/K28H/Q32H and Cu^II^–NTA (see Supporting Information). Isothermal titration calorimetry (ITC) data predicted *K*_D_ values in the range from 5 to 42 μm for all construct/Cu^II^‐label permutations (see Supporting Information). This is in line with previous literature reporting low μm *K*_D_ for dH–Cu^II^.^20^ Importantly, the ITC analysis also yields a binding enthalpy that allows predicting the dissociation constant as a function of temperature according to van't Hoff. Here, binding is exothermic so at lower temperature tighter binding is expected. Δ*H* in kcal mol^−1^ are −9.68 (NTA‐6H8H), −7.54 (NTA‐28H32H), −3.37 (IDA‐6H8H), and −5.75 (IDA‐28H32H). Closer inspection reveals that extrapolating to 235 to 240 K, the RIDME‐determined *K*_D_s agree remarkably well with the ITC data. As RIDME experiments are performed at 30 K, there will be no dissociation or association in the frozen sample, so the RIDME data will reflect the binding equilibrium when the dynamics of binding and releasing are frozen (see Supporting Information). These extrapolations, previous binding studies,6c and the ITC data all suggest that the binding equilibrates fast. Even when snap‐freezing a room temperature sample by immersion into liquid nitrogen, the equilibrium reflects a 50 to 60 K lower temperature.

**Figure 2:**
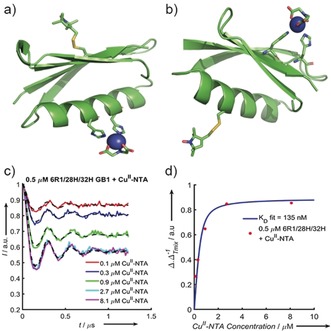
Cartoon structural representations of GB1 constructs I6R1/K28H/Q32H (a) and I6H/N8H/K28R1 (b). The histidine residues that form the dH site and the R1 labels are shown as sticks; the Cu^II^–NTA label is modelled with the Cu^II^‐centre shown as a blue sphere. c) Background‐corrected 5‐pulse RIDME traces of 500 nm I6R1/K28H/Q32H GB1 measured as a pseudo‐titration with 100–8100 nm Cu^II^–NTA. The experimental data are coloured according to the plot legend with the corresponding fits shown as dashed black lines. d) Experimental Δ as a quotient of $\Delta_{Tmix}$ [calculated using Eq. (1)], extracted from the Cu^II^–NTA pseudo‐titration series, (Figure 2 c), as a function of Cu^II^–NTA concentration (red dots) with the fitted binding isotherm in blue.

With the aim of unequivocally demonstrating the sub‐μm *K*_D_ of I6R1/K28H/Q32H and Cu^II^–NTA in frozen samples and the feasibility of RIDME studies at these concentrations a pseudo‐titration was measured at 500 nm I6R1/K28H/Q32H, in the presence of 100 to 8100 nm Cu^II^–NTA spin label (Figure 2 c). Qualitatively, Δ increases with increasing Cu^II^–NTA, before plateauing towards 0.5, as is expected according to Equation (1). The fit of the experimental $\Delta \times {\Delta_{Tmix}}^{-1}$ values to Equation (2) approximates a *K*_D_ value of 150–300 nm (see the Supporting Information), which is consistent with the prediction at higher protein concentration. Interestingly, the observed Δ values do not reach the asymptotic limit of 0.5; rather, values tend to approximately 0.45.6a Thus, we chose to scale Δ and to employ bivariate fitting to Equation (2) for the binding isotherm (Figure 2 d). Scaled results are virtually identical to fixing the asymptotic value of Equation (1) to 0.45 (see the Supporting Information).

In summary, it has been demonstrated that due to the exothermic nature of the binding process, the α‐helical *i* and *i+4* dH site has a sub‐μm affinity for Cu^II^–NTA under cryogenic EPR conditions. This has important implications for the use of Cu^II^‐chelators as spin labels, and particularly for their application to double‐dH constructs. The increased sensitivity afforded by both Q‐band RIDME in Cu^II^–nitroxide and Cu^II^–Cu^II^ systems compared with Cu^II^–Cu^II^ PELDOR experiments is truly promising; our sensitivity determination^21^ gives improvements by factors of approximately 150 and approximately 100, respectively (see the Supporting Information). Similarly, Ritsch et al. have shown that Cu^II^–nitroxide RIDME outperforms PELDOR by a significant margin, even in a state‐of‐the‐art ultra‐wide bandwidth spectrometer.^22^ Importantly, RIDME is also less prone to orientation selection than PELDOR, facilitating distance extraction.^23^ This sensitivity range opens up the possibility of routine distance measurements at greatly reduced protein concentration, making new systems accessible and potentiating new science.^24^ This work further demonstrates how RIDME can be used to measure binding equilibria, and subsequently determine *K*_D_s remotely via the dipolar interaction under experimental EPR conditions. This strategy is important as it couples structural and thermodynamic information for protein–ligand interactions and could be applied to ligand‐gated systems. The approach is complementary to calorimetric methods, such as ITC, especially in the study of high‐affinity interactions, where other methods of structural investigation are often sensitivity‐limited. Used together, we anticipate that dH sites and the RIDME experiment can expand the PDEPR tool‐box to the study of protein systems previously beyond reach. In conclusion, dH affinity for Cu^II^‐chelators is not limiting for PDEPR studies, and *K*_D_ values can be directly estimated from 5‐pulse RIDME data and agree well with low‐temperature extrapolated ITC data. Furthermore, this study demonstrates that using spectroscopically orthogonal spin labels, such as Cu^II^–NTA and nitroxide, in combination with sub‐μm PDEPR experiments is feasible.

## Conflict of interest

The authors declare no conflict of interest.

## Supporting information

As a service to our authors and readers, this journal provides supporting information supplied by the authors. Such materials are peer reviewed and may be re‐organized for online delivery, but are not copy‐edited or typeset. Technical support issues arising from supporting information (other than missing files) should be addressed to the authors.

Supplementary
