# Supplementary material for: Sub‐Micromolar Pulse Dipolar EPR Spectroscopy Reveals Increasing CuII‐labelling of Double‐Histidine Motifs with Lower Temperature
Source: Angew Chem Int Ed Engl. 2019 Jul 18;58(34):11681–5. doi: 10.1002/anie.201904848 (PMC6771633; doi:10.1002/anie.201904848)
Supplement: Supplementary file 1 — Supplementary [file ANIE-58-11681-s001.pdf]

Angewandte Chemie
Eine Zeitschrift der Gesellschaft Deutscher Chemiker

Supporting Information

Sub-Micromolar Pulse Dipolar EPR Spectroscopy Reveals Increasing Cu^{II}-labelling of Double-Histidine Motifs with Lower Temperature

*Joshua L. Wort, Katrin Ackermann, Angeliki Giannoulis, Alan J. Stewart, David G. Norman, and Bela E. Bode**

anie_201904848_sm_miscellaneous_information.pdf

Table of Contents

I **Experimental Procedures**……2

1.1 *Construct Design*……2

1.2 *Protein Expression and Purification*……2

1.3 *MTSL-labelling and Pulse EPR Sample Preparation*……3

1.4 *EPR Instrumentation*……3

1.5 *Parameters of Electron Longitudinal Relaxation Time Measurements*……4

1.6 *Parameters of Electron Transverse Relaxation Time Measurements*……4

1.7 *Parameters of PELDOR Measurements*……4

1.8 *Parameters of RIDME Measurements*……5

1.9 *Sensitivity Optimisation of RIDME and PELDOR Measurements*……5

1.10 *RIDME Data Processing and Analysis*……6

1.11 *Mass Spectrometry*……6

1.12 *Continuous-Wave EPR Sample Preparations*……7

1.13 *Circular Dichroism Spectroscopy and Thermal Denaturation Assays*……7

1.14 *Isothermal Titration Calorimetry Measurements*……7

1.15 *Molecular Dynamics Simulations and MMM*……7

1.16 *UV-visible Spectroscopy*……8

II **Results and Discussion**……8

2.1 *SDS-PAGE Gel Electrophoresis*……8

2.2 *Continuous-Wave EPR Measurements*……10

2.3 *Mass Spectrometry*……11

2.4 *Cu^{II}-label Concentration Quantification by UV-visible Spectroscopy*……12

2.5 *Circular Dichroism Spectra and Thermal Denaturation Assays*……15

2.6 *Isothermal Titration Calorimetry*……16

2.7 *Inversion Recovery*……18

2.8 *Electron-spin Echo Decay Measurements*……23

2.9 *Q-band RIDME*……26

2.10 *X-band PELDOR*……34

2.11 *Sensitivity Optimisation of Cu^{II}-Cu^{II} RIDME and PELDOR Measurements*……36

2.12 *RIDME Sensitivity Estimation*……38

2.13 *25 µM and 75 µM RIDME Pseudo-Titration Series*……41

2.14 *The Influence of T_1 and Δ_{Tmix} on K_D Estimation from Modulation Depth Quantitation*……43

2.15 *Theoretical Extension of Modulation Depth Quantitation to a Two-site Binding Model*……44

III **References**……47

IV **Author Contributions**……47

Experimental Procedures

1.1 Construct Design:

The full-length Group G *Streptococcus* I6H/N8H/K28H/Q32H mutant protein G-B1 domain (GB1) gene was inserted into the pET11a plasmid vector without N- or C-terminal affinity tags and under the control of the *lac* operon. Nucleotide primers were ordered internally from the University of Dundee and the first round of mutagenesis (QuikChange Lightning Site-Directed Mutagenesis Kit, Agilent) produced the following two GB1 constructs: I6H/N8H and K28H/Q32H, before a second round produced I6H/N8H/K28C and I6C/K28H/Q32H, with primer and construct sequences given in figures S1 and S2, respectively. The PCR products were then treated with the endonuclease *dpn1*, transformed into XL10-Gold Ultracompetent cells (Agilent), and the mutations and sequence fidelity were confirmed *via* DNA extraction and purification (QIAprep® Spin Miniprep Kit 250) followed by DNA sequencing (Dundee University DNA Sequencing and Services).

I6H/N8H GB1 Forward Primer Sequence:
5' GCT GCT ACC GCG GAA AAG GTT TTC AAA CAG TAC GCT AAC G 3'

I6H/N8H GB1 Reverse Primer Sequence:
5' CGT TAG CGT ACT GTT TGA AAA CCT TTT CCG CGG TAG CAG C 3'

K28H/Q32H GB1 Forward Primer Sequence:
5' CAG TAC AAG CTT ATC CTG AAC GGT AAA ACC CTG AAA GGT G 3'

K28H/Q32H GB1 Reverse Primer Sequence:
5' CAC CTT TCA GGG TTT TAC CGT TCA GGA TAA GCT TGT ACT G 3'

6H/8H/K28C GB1 Forward Primer Sequence:
5' GTC GAC GCT GCT ACC GCG GAA TGC GTT TTC AAA 3'

6H/8H/K28C GB1 Reverse Primer Sequence:
5' TTT GAA AAC GCA TTG CGC GGT AGC AGC GTC GAC 3'

I6C/28H/32H GB1 Forward Primer Sequence:
5' GCA GTA CAA GCT TTG CCT GCA CGG TAA AAC CCT G 3'

I6C/28H/32H GB1 Reverse Primer Sequence:
5' CAG GGT TTT ACC GTG CAG GCA AAG CTT GTA CTG C 3'

Figure S1. Forward and reverse nucleotide primer sequences used in the mutagenesis process for each GB1 construct.

I6H/N8H/K28C GB1 Protein Sequence:
6 8 28
MQYKLHLHGKTLKGETTTEAVDAATAECVFKQYANDNGVDGEWTYDDATKTFTVTE

I6C/K28H/Q32H GB1 Protein Sequence:
6 28 32
MQYKLCLNGKTLKGETTTEAVDAATAEHVFKHYANDNGVDGEWTYDDATKTFTVTE

Figure S2. Full amino-acid sequence for both I6H/N8H/K28C and I6C/K28H/Q32H GB1 constructs, with the cysteine residue for R1-labelling in red, and each histidine residue of the double-histidine motifs shown in blue; and residue number indicated above the sequence in each case.

1.2 Protein Expression and Purification:

The I6H/N8H/K28H/Q32H construct was expressed and purified as published previously.[1] The sequenced plasmid vectors for the other constructs were transformed into expression-strain BL21 (DE3) cells and grown in Luria-Bertani (LB) medium (50 µg/ml Ampicillin Sodium (Formedium™)) *via* incubation (37 °C, 180 rpm) until OD = 0.6-0.8, before expression was induced with 0.5 mM isopropyl-β-D-1-galactopyranoside (IPTG) (Formedium™) for 4 hours. Cultures were pelleted *via* centrifugation (45 minutes, 6,000 × *g*). Pellets were resuspended in lysis buffer (20 mM Tris-HCl, 5 mM NaCl, 0.1% v/v Triton X-100, 0.5 mg/mL hen-egg lysozyme (Sigma Aldrich), DNAse (Sigma Aldrich), cOmplete™ mini EDTA-free protease inhibitor cocktail (Roche), pH 8.5) and sonicated immediately. The 6C/28H/32H construct was lysed in a volume of lysis buffer corresponding to 10 × the pellet weight, for the 6H/8H/28C construct half of that volume (5 × pellet weight) was used. Cellular resuspensions were lysed *via* sonication (6 × 30 seconds pulsed, 30 seconds

off, 12 µm amplitude), and then heat-shocked (80 °C, 10 minutes) and centrifuged (30 minutes, 48,380 × *g*, 4 °C). The supernatant was sterile-filtered (0.2 µm hydrophilic membrane filter, Millex®, Sigma Aldrich) before loading onto a pre-equilibrated anionic exchange column (5 mL Hi-Trap™ Q HP). The column was subsequently washed with 10 column volumes of 20 mM Tris-HCl, 5 mM NaCl, pH 8.5 (buffer A), protein was eluted *via* a gradient over 8 column volumes to 50% 20 mM Tris-HCl, 1M NaCl, pH 8.5 (buffer B) and fraction purity for each construct was assayed *via* SDS-PAGE (sodium dodecyl sulfate polyacrylamide gel electrophoresis). In case of the 6H/8H/28C construct the majority of the protein was found in the flow-through of the HiTrap column. The flow-through was therefore diluted 1:1 with buffer A and reloaded using 2 × 5 mL Hi-Trap™ Q HP columns in tandem to avoid potential overloading. Fractions containing GB1 were pooled and subsequently concentrated *via* 3,000 Da MWCO (molecular weight cut-off) centrifugal concentrators (Pall Microsep™ Advance), with final sample purity being assessed *via* MALDI-TOF mass spectrometry, as shown in section 2.3, and SDS-PAGE, as shown in section 2.1, before proceeding with MTSL-labelling. All gel electrophoresis was performed with 4-12% polyacrylamide gels (NuPAGE), run at a constant 200 V for 30 minutes. As marker a broad range protein ladder (10-180 kDa) was used (PageRuler™ Pre-stained Protein Ladder) (ThermoFisher™).

1.3 MTSL-labelling and Pulse EPR Sample Preparation:

The I6C/K28H/Q32H and I6H/N8H/K28C GB1 constructs were both labelled with *S*-[(1-oxyl-2,2,5,5-tetramethyl-2,5-dihydro-1H-pyrrol-3-yl)methyl] methanesulfonothioate (MTSL) (Santa Cruz™ Biotechnology) *via* an initial incubation in 10 mM dithiothreitol (DTT) (Formedium™) (4 °C, 2 hours) to fully reduce the sulfhydryl moiety of the cysteine residues. DTT was then removed *via* a PD10 column (Amersham Biosciences) equilibrated in buffer A with a single elution fraction of 3.5 mL which was incubated with 10-fold molar excess MTSL spin label (4 °C, 16 hours). To remove free MTSL label, material was loaded on a HiLoad 26/600 Superdex 200 pg size-exclusion chromatography column equilibrated in 42.4 mM Na_2HPO_4, 7.6 mM KH_2PO_4, 150 mM NaCl, pH 7.4 (buffer C). Fractions containing GB1 were pooled and concentrated. The extent of spin labelling was assayed *via* MALDI-TOF mass-spectrometry and labelling efficiency was calculated using CW-EPR, as shown in section 2.2. Exchange of the quantitatively labelled material into deuterated buffer was performed by freeze-drying protein and re-dissolving in D_2O. Addition of 50% (v/v) ethylene glycol (EG) *d-6* (Deutero GmbH) ensured formation of a glassy frozen solution, after the addition of Cu^{II}-iminodiacetic acid (Cu^{II}-IDA) or Cu^{II} nitrilotriacetic acid (Cu^{II}-NTA) label, to a total volume of 70 µL, unless otherwise stated. The influence of buffer and cryoprotectant conditions was investigated, shown in section 2.8, by preparation of 75 µM I6R1/K28H/Q32H GB1 + 75 µM Cu^{II}-IDA either in 50 mM phosphate buffer (buffer C) or N-ethyl morpholine (NEM) buffer and with either 50% deuterated EG or 20% deuterated glycerol (gly) as cryo-protectants. All EPR samples were frozen by direct immersion into liquid nitrogen.

Cu^{II}-IDA and Cu^{II}-NTA stock solutions were prepared from anhydrous $CuCl_2$ (VWR™), IDA ($C_4H_5NO_4Na_2$, sodium iminodiacetate dibasic hydrate; Sigma Aldrich®), and NTA ($C_6H_9NO_6$; Sigma Aldrich®). For Cu^{II}-NTA, a 100 mM $CuCl_2$ solution in deionized H_2O at pH 2.0, and a 100 mM NTA solution in deionized H_2O at pH 12.0 were prepared. The NTA stock solution was diluted 1:8 in buffer C, before slowly adding 1 part of the $CuCl_2$ stock solution, to give a nominal concentration of 10 mM Cu^{II}-NTA.[2] Cu^{II}-IDA stock solution was prepared by mixing equal volumes of 200 mM $CuCl_2$ solution in deionized H_2O with 200 mM IDA in deionized H_2O to a final concentration of 100 mM Cu^{II}-IDA. For the Cu^{II}-labelling of the protein, Cu^{II}-IDA and Cu^{II}-NTA stock solutions were freeze-dried and re-dissolved in D_2O to final concentrations of 100 and 10 mM, respectively.

1.4 EPR Instrumentation:

All pulse EPR experiments were performed using a Bruker ELEXSYS 580 pulse EPR spectrometer. Temperatures were maintained using a cryogen-free variable temperature cryostat (Cryogenic Ltd) operating in the 3.5-300 K temperature range. All measurements of the electron spin longitudinal relaxation times (T_1) of Cu^{II}-IDA and Cu^{II}-NTA, and all 5-pulse dead-time free RIDME measurements[3] were performed at 30 K, using a high-power 150 W travelling-wave tube (TWT; Applied Systems Engineering) at Q-band (34 GHz) in a critically coupled 3 mm cylindrical resonator (Bruker ER 5106QT-2w in TE012 mode). All 4-pulse dead-time free PELDOR measurements[4] were performed at 15 K at X-band (9.4 GHz) with a 1 kW TWT (Applied Systems Engineering) in an over-coupled 3 mm split-ring resonator (Bruker 4118X-MS3), unless otherwise stated. All CW-EPR measurements were performed using a Bruker EMX 10/12 spectrometer equipped with an ELEXSYS Super Hi-Q resonator, at X-band frequencies (9.8 GHz) and at 298 K.

1.5 Parameters of Electron Longitudinal Relaxation Time Measurements:

The 3-pulse (π-T-π/2-τ-π) inversion recovery (IR) experiment was used and applied at the maximum field position of the Cu^{II} spectrum (figure S3), a 12 ns inversion pulse (nominal flip-angle π) was used and 20 and 40 ns pulses (π/2- and π, respectively) were used for the observer subsequence. Traces were acquired to 500 μs and the time interval T was incremented in steps of 200 ns, using a shot repetition time (SRT) of 2 ms (unless otherwise stated) and a τ of 800 ns. Raw data were fitted with mono- and bi-exponential functions, shown in equations 1 and 2, respectively, to estimate T_1:

$$y = M_0\left[1 - 2a \times exp\left(\frac{-x}{T_1}\right)\right] \quad (1)$$

$$y = M_0\left[1 - 2a\left(b \times exp\left(\frac{-x}{T_{1A}}\right) + (1-b) \times exp\left(\frac{-x}{T_{1B}}\right)\right)\right] \quad (2)$$

The relative observer positions used in the inversion recovery and RIDME measurements are indicated with arrows on the Cu^{II}-IDA and nitroxide spectra respectively, shown below in figure S3.

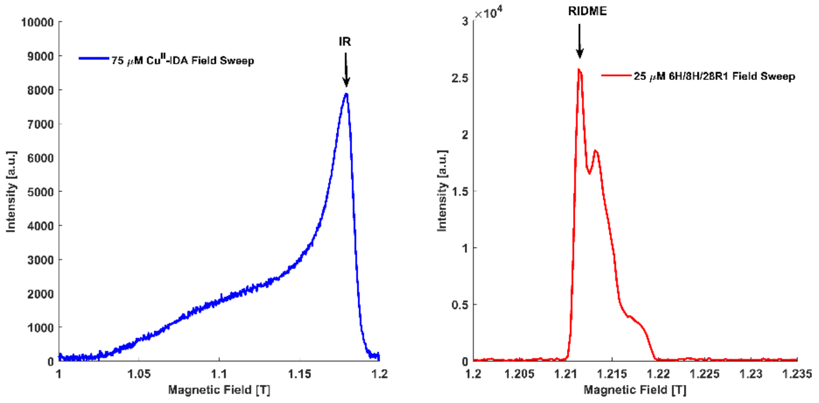

Figure S3. Echo detected field sweeps of 75 μM Cu^{II}-IDA (left panel), with an arrow indicating the observer field used for the inversion recovery (IR) experiments, and 25 μM I6H/N8H/K28R1 GB1 (right panel) with an arrow indicating the observer field used for the RIDME experiments. The Cu^{II}-IDA spectrum is shown in blue, and the nitroxide spectrum is shown in red.

1.6 Parameters of Electron Transverse Relaxation Time Measurements:

The 2-pulse (π/2-τ-π) electron spin-echo decay (ESE) experiment was applied at either the maximum field position of the nitroxide or Cu^{II}-chelate spectrum (figure S3), using 16 and 32 ns π/2- and π-pulses. Traces were acquired to various time windows between 10-20 μs depending on the measurement temperature in the range 10-50 K, using a τ of 800 ns (due to dead-time in high Q mode), and was incremented in steps of 8 ns. The SRT used varied depending on the measured temperature and detected species. Raw data was fitted with stretched-exponential functions, where the stretching exponent *x*, is constrained to values between 1 and 2, and is given in equation 3, to estimate T_m:

$$y = M_0\left[exp\left(\left(\frac{-\tau}{T_m}\right)^x\right)\right] \quad (3)$$

1.7 Parameters of PELDOR Measurements:

For the Cu^{II}-nitroxide X-band PELDOR measurements the 4-pulse experiment (π/2($υ_A$)-$τ_1$-π($υ_A$)-$τ_1$-t-π($υ_B$)-($τ_2$-t)-π($υ_A$)-t_2-echo) was used, where $υ_A$ and $υ_B$ indicate the pulse excitation at the observer and pump frequencies, respectively. In all cases pulses of lengths 16 and 32 and 10 ns were used for observer and pump pulses (π/2, π and π). The magnetic field and microwave frequency were adjusted for the maximum of the nitroxide spectrum to coincide with the pump pulse position, while the observer pulse was placed at 140 MHz higher frequency for detection of spins centred on copper ions and minimal overlap of pump and dection excitation bands. Due to strong electron spin echo envelope modulation (ESEEM) induced by deuterium nuclei a $τ_1$ of 470 ns was chosen, to maximise electron spin echo intensity, and thus improve the signal-to-noise ratio. The I6H/N8H/K28R1 and I6R1/K28H/Q32H GB1 constructs

were measured at concentrations of 220 and 250 µM, and at sample volumes of 15 and 25 µL, respectively, in presence of 1.5 molar equivalents of each Cu^{II}-chelate.The I6H/N8H/K28R1 Cu^{II}-chelate samples were measured with a time window of 1360 ns, a dipolar increment of 8 ns, 135 points, 50 shots-per-point and were averaged for 12 and 16 hours for Cu^{II}-IDA and Cu^{II}-NTA, respectively. The I6R1/K28H/Q32H Cu^{II}-IDA sample was measured with a time window of 1900 ns, a dipolar increment of 8 ns, 180 points, 50 shots-per-point and averaged for 3 hours. The Cu^{II}-NTA sample was measured with a time window of 1160 ns, a dipolar increment of 8 ns, 96 points, 50 shots-per-point and averaged for 3 hours.

The I6H/N8H/K28H/Q32H GB1 construct was measured using the 4-pulse PELDOR experiment at 30 K and Q-band frequency, at a concentration of 25 µM and at a sample volume of 100 µL, in presence of 2.0 molar equivalents of Cu^{II}-NTA. This was performed on the Bruker ELEXSYS 580 pulse EPR spectrometer described in section 1.4; the Bruker ER 5106QT-2w in TE012 mode resonator was overcoupled, pulses of lengths 16 and 32 and 24 ns were used for observer and pump pulses (π/2, π and π). Magnetic field and microwave frequency were adjusted for the maximum of the Cu^{II}-NTA spectrum to coincide with the pump pulse position, while the observer pulse was placed at 80 MHz higher frequency. The sample was measured with a time window of 1280 ns, a dipolar increment of 8 ns, 122 points, 1 shot-per-point and 1 scan, for the purpose of sensitivity comparison with Cu^{II}-Cu^{II} and Cu^{II}-NO RIDME.

1.8 Parameters of RIDME Measurements:

The 5-pulse RIDME experiment ($\pi/2$-τ_1-π-τ_1-t-$\pi/2$-T_{mix}-$\pi/2$-(τ_2-t)-π-τ_2-echo) was used (figure S4). Rectangular pulses of lengths 12 and 24 ns were used (π/2 and π, respectively), consistently placing the detection frequency to the maximum of the nitroxide spectrum. All acquisitions were performed over a single scan (unless otherwise stated), with two shots per point, with a SRT of 30 ms, and deuterium ESEEM was suppressed *via* a 16-step nuclear modulation *τ*-averaging cycle.[5] Signal contributions from unwanted echoes were eliminated using an 8-step phase-cycle, totalling 128 steps per measurement, with the refocused virtual echo (RVE) being detected. For all samples, at least two lengths of mixing block were recorded; a short reference mixing time (T_{ref}) and a long mixing time (T_{long}) to allow suppression and observation of the dipolar coupling, respectively. The I6H/N8H/K28R1 and I6R1/K28H/Q32H GB1 constructs were measured at 5 µM, with one equivalent of each Cu^{II}-chelate, and at a sample volume of 75 µL. The RIDME traces of the pseudo-titration performed at 0.5 µM I6R1/K28H/Q32H GB1 in presence of varying Cu^{II}-NTA concentrations were also recorded in the same manner, and at a sample volume of 75 µL.

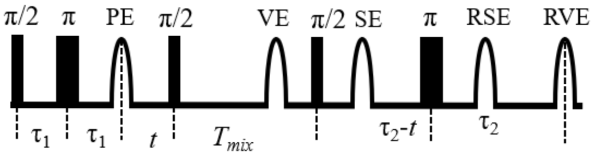

Figure S4. 5-pulse RIDME experiment. Here the refocused virtual echo (RVE) is observed, and is modulated by the electron-electron dipolar coupling as a function of *t*. This defines the position of the third and fourth pulses, which comprise a longitudinal mixing block, separated by the fixed interval T_{mix}. The dipolar coupling manifests from intrinsic electron spin longitudinal relaxation events (Δms) during the mixing block, after which dipolar evolution occurs during the interval τ_2. The remaining echoes generated by the sequence are indicated; primary echo (PE), stimulated echo (SE), virtual echo (VE) and refocused stimulated echo (RSE), separated by 2τ from the detected RVE.

1.9 Sensitivity Optimisation of RIDME and PELDOR Measurements:

It was necessary to optimise the sensitivity of the RIDME experiments for both Cu^{II}-Cu^{II} and Cu^{II}-nitroxide spin systems before performing the pseudo-titration series in figure 2d of the main text. An expression for the sensitivity in the 5-pulse RIDME experiment was adapted from the sensitivity expression for 4-pulse PELDOR[17] and is given below:

$$\frac{S}{N_{RIDME}} \sim \frac{1}{T} exp\left(\frac{-2t_{max}}{T_{m_A}}\right)\frac{1}{\sqrt{T_{1_A}}} exp\left(\frac{-T_{mix}}{T_{1_A}}\right)\left(\frac{\left(1 - exp\left(\frac{-T_{mix}}{T_{1_B}}\right)\right)}{2}\right) \tag{4}$$

Here, the subscripts A and B describe the detected and inverted (fast-relaxing) species respectively, T is the temperature in Kelvin, T_1 and T_m are the longitudinal relaxation and transverse dephasing times, respectively, t_{max} is the dipolar evolution time window,

and T_{mix} is the mixing block interval. It should be noted that the additional terms added to the literature expression (the Boltzmann, transverse dephasing and averaging terms are valid as in PELDOR) describe longitudinal relaxation and subsequent loss of detected spin magnetisation, and modulation depth arising from the longitudinal relaxation of non-resonant spins, both during the interval T_{mix}. Since actual electron spin echo decay data was fitted using a stretched exponential and inversion recovery data was best fitted biexponentially, both of these forms were subsequently incorporated into the sensitivity expression above:

$$\frac{S}{N_{RIDME}} \sim \frac{1}{T} exp\left(\left(\frac{-2t_{max}}{T_{2_A}}\right)^x\right)\frac{1}{\sqrt{T_{1_A}^{fast}}}\left(bexp\left(\frac{-T_{mix}}{T_{1_A}^{fast}}\right)+(1-b)exp\left(\frac{T_{mix}}{T_{1_A}^{slow}}\right)\right)\left(\frac{\left(1-\left(bexp\left(\frac{-T_{mix}}{T_{1_B}^{fast}}\right)+(1-b)exp\left(\frac{-T_{mix}}{T_{1_B}^{slow}}\right)\right)\right)}{2}\right) \quad (5)$$

It should be noted that for a homo-spin pair, the optimum mixing block interval (T_{mix}) can be determined by finding the maximum of the product of the longitudinal relaxation loss factor and the modulation depth build-up (since $T_{1A} = T_{1B}$):

$$f(T_{mix}) = exp\left(\frac{-T_{mix}}{T_{1_A}}\right)\left(\frac{\left(1-exp\left(\frac{-T_{mix}}{T_{1_B}}\right)\right)}{2}\right) \quad (6)$$

Here, maximum sensitivity is obtained when the ratio between T_{mix} and T_1 is approximately 0.7. Sensitivity estimates were also calculated for Cu^{II}-Cu^{II} PELDOR[1] with the addition of the stretched exponential in the transverse dephasing term, as is given in equation 7.

$$\frac{S}{N_{PELDOR}} \sim \frac{1}{T} exp\left(\left(\frac{-2t_{max}}{T_{2_A}}\right)^x\right)\frac{1}{\sqrt{T_{1_A}}} \quad (7)$$

Sensitivity contours for a systematic variation of multiple parameters are given and discussed in more depth in section 2.11.

1.10 RIDME Data Processing and Analysis:

Unless otherwise stated all RIDME traces were pre-processed deconvoluting imperfections by a division in the time domain of the long mixing time (T_{long}) trace by the short mixing time (T_{ref}) trace. This pre-processing subsequently allowed an adequate fit of a second-order polynomial background function and facilitated modulation depth quantitation of the deconvoluted traces in DeerAnalysis2015.[6] Within a limited range stretched exponential background functions can be reasonably well fitted by polynomials. To minimise the error manifesting from background correction, values for background start and cut-off were used that were robust against changes in modulation depth; that is, the modulation depth remained constant when background start and cut-off points were varied. As the division by the reference trace already eliminates some dipolar contribution to the time trace the modulation depths were then re-scaled according to equation 8, where ΔT_{long} and ΔT_{ref} are the long and short mixing-time calculated modulation depths, respectively.

$$\Delta_c = \left(1-\frac{1-\Delta_{Tlong}}{1-\Delta_{Tref}}\right) \quad (8)$$

The magnitude of Δ as a function of time T_{mix} (T_{long} or T_{ref}) and T_1 is given in eq. 1 of the main text.[7] The fractional occupancy of the double-histidine site is then given by the quotient: $\Delta_C \times \Delta_{Tmix}{}^{-1}$, which tends asymptotically towards 1, for increasing occupation of the double-histidine site and fixed mixing time; division by Δ_{Tmix} ideally removes the effect of T_1 varying between samples in the pseudo-titration series. A K_D value can be estimated by minimizing a univariate or bivariate error function to eq. 2 in the main text, in the case of the latter the asymptotic limit of Δ_{Tmix} is also allowed to vary; different approaches to fitting K_D are discussed further in section 2.14.

1.11 Mass Spectrometry:

All mass spectra were collected in-house using a Sciex Matrix Assisted Laser Desorption/Ionisation (MALDI) TOF/TOF 4800 mass-spectrometer, with samples crystallised using a matrix of α-cyano-4-hydroxycinnamic acid. Unlabelled and MTSL-labelled

I6H/N8H/K28C and I6C/K28H/Q32H GB1 samples were both prepared at 20 μM concentration in 20 mM Tris-HCl, 5 mM NaCl, pH 8.5 or 42.4 mM Na_2HPO_4, 7.6 mM KH_2PO_4, 150 mM NaCl, pH 7.4, respectively, and mass spectra were recorded in the absence of Cu^{II}.

1.12 Continuous-Wave EPR Sample Preparations:

All samples were measured at 100 μM protein concentration, using disposable 50 μL capacity micropipettes (BlauBrand®) and MTSL-labelling efficiency for each construct was determined by taking the double integral and numerically calibrating with free MTSL label of known concentration; in all cases measured labelling efficiency was ≥ 95%, and samples showed negligible residual free spin label contribution.

1.13 Circular Dichroism Spectroscopy and Thermal Denaturation Assays:

All measurements were performed using a MOS-500 circular dichroism spectrometer (BioLogic) with an ALX-300 Hg lamp in the far-UV range (180-260 nm). Samples were measured in a 0.1 mm quartz cuvette at a protein concentration of 50 μM, in the presence and absence of 150 μM Cu^{II}-IDA, all in 10 mM sodium phosphate, pH 7.4. Before and after each sample measurement, a blank was taken containing only 10 mM sodium phosphate, pH 7.4 (and 150 μM Cu^{II}-IDA where appropriate) to be subtracted from the raw data for baseline correction. All measurements were performed using an acquisition period of 0.5 seconds, a 0.25 nm step-size, at 298 K, and using a 2 nm bandwidth from 200-260 nm.

For the thermal denaturation curves, two wavelengths (220 and 260 nm) were monitored over the temperature range 20-98 ºC using a TCU250 Peltier temperature controller. The two wavelengths reported changes in the global minimum of the spectra, and in the background signal, respectively. The thermal profile used 39 intervals of 2 ºC in the range 20-98 ºC, and a 120 second equilibration interval at each temperature prior to measurement. As above, measurements were baseline corrected with 10 mM sodium phosphate, pH 7.4 (+ 150 μM Cu^{II}-IDA where appropriate). Finally, the melting curves were fitted using a two-state unfolding model to extract the melting temperatures (T_M).

1.14 Isothermal Titration Calorimetry Measurements:

All isothermal titration calorimetry experiments used a Malvern MicroCal ITC200 instrument, and were optimised and performed over 19 injections of 2 μL titrant, with an equilibration time of 150 seconds between injections, at 298 K. All samples were centrifuged immediately before measurement for degassing. All protein was measured at 75 μM concentration and titrant concentrations were either 1 or 2 mM, as specified in section 2.6; therefore blank conditions of buffer C and titrated against addition of 1 or 2 mM titrant were recorded, to be subtracted from the raw data and mitigate the heat of dilution; all data analyses were performed in MicroCal Origin 7 (OriginLab, Northampton, MA) and thermodynamic parameters were derived using a single-site fitting model. To this end eq. 9 and 10 were used, given below, where: *Q(i)* is the heat content of the solution at the point of the *ith* injection, *K* is the binding constant, *n* is the number of sites, *ΔH* is the molar heat of ligand binding, V_0 is the active cell volume, M_t is the total concentration of macromolecule contained in V_0, X_t is the total concentration of ligand contained in V_0 and *ΔQ(i)* is the change in heat content of the solution between the *(i-1)th* and the *ith* injection and *δVi* is the injection volume. Standard Marquardt methods are then applied to minimise the deviation between the estimated values of *n*, *ΔH* and *K* and the experimental values determined from *Q(1,2...i-1,i)*.

$$Q_{(i)} = \frac{nM_t \Delta H V_0}{2}\left[1 + \frac{X_t}{nM_t} + \frac{1}{nKM_t} - \sqrt{\left(1 + \frac{X_t}{nM_t} + \frac{1}{nKM_t}\right)^2 - \frac{4X_t}{nM_t}}\right] \tag{9}$$

$$\Delta Q_{(i)} = Q_{(i)} + \frac{\partial V_i}{V_0}\left[\frac{Q_{(i)} + Q_{(i-1)}}{2}\right] - Q_{(i-1)} \tag{10}$$

1.15 Molecular Dynamics Simulations and MMM:

All molecular dynamics simulations were performed in XPLOR-NIH.[8] Mutations were inserted in rotamer populations that minimised steric strain using the mutagenesis tool in PyMOL (The PyMOL Molecular Graphics System, Version 2.0 Schrödinger, LLC) before building of the MTSL and Cu^{II}-IDA label using a script written in-house. Models were then stabilised with iterative minimisation *via* Powell's conjugate direction method[9] (*n* = 50) and molecular motion was simulated using Verlet integration[10] at 200 K for 100 trials; between 103-105 structures were collected in the time interval of 25 ns, in steps of 0.25-2.5 ps. In all cases, a second order

harmonic constraint was applied to the protein backbone and a planarity constraint maintained the octahedral geometry of Cu^{II}-IDA coordinated to the respective δ-nitrogen atoms of the imidazole rings. The rotamer libraries generated from the molecular dynamics for the R1 label were also compared to those generated using the MTSSL Wizard tool in Pymol[11] and were found to be in reasonable agreement.

All MMM modelling was performed using MMM2018.[12] In the I6H/N8H/K28H/Q32H GB1 crystal structure (PDB ID: 4WH4[1]) histidine residues at positions 28 and 32 were replaced, respectively, with cysteine and glutamine residues, on chain A of the crystal structure. A Cu^{II}-IDA label (using the 'symmetric' mode) was introduced at position 6, along with an R1 label at position 28, to simulate the I6H/N8H/28R1 GB1 construct. The process was then repeated, instead with replacement of the histidine residues at positions 6 and 8 with cysteine and asparagine, respectively, and insertion of R1 at position 6 to simulate the 6R1/K28H/Q32H construct. PELDOR traces were simulated using the 'ambient' temperature option.

1.16 UV-visible Spectroscopy:

UV-visible spectroscopy was employed to quantify Cu^{II}-IDA and Cu^{II}-NTA concentrations. Spectra were recorded using a Varian 50 bio UV-visible spectrophotometer with a Xe flash lamp and a wavelength resolution of 1 nm, all measurements were performed in single beam mode, with a buffer blank being run prior to measuring experimental conditions. For quantification, Cu^{II}-IDA samples of concentrations 25.4, 12.7, 5.1, and 2.5 mM and Cu^{II}-NTA samples of concentrations 10.0, 7.5, 5.0, 2.5 and 1.0 mM were prepared as described in chapter 1.3, and were respectively blanked against milliQ H_2O and buffer C. All samples were measured 3-4 times in the range 300-800 nm in a plastic cuvette with path-length of 10 mm and spectra were analysed and plotted using the Cary software package.

Results and Discussion

2.1 SDS-PAGE Gel Electrophoresis:

Figure S5 shows SDS-PAGE gels of the expression profiles for I6C/K28H/Q32H and I6H/N8H/K28C GB1 constructs, in the left and right panels, respectively. Each expression in 1 L culture was duplicated, and so at each time-point samples are taken from both inocula.

For the I6C/K28H/Q32H construct, a band can be seen to appear between 10-15 kDa and between one- and four-hours post-induction using 500 µM IPTG (Formedium™); this corresponds to GB1, which runs on PAGE-gels as a ~13 kDa band. For the I6H/N8H/K28C construct the expression profile is similar, except the band corresponding to GB1 is more pronounced throughout the expression: being an intense feature even after one hour.

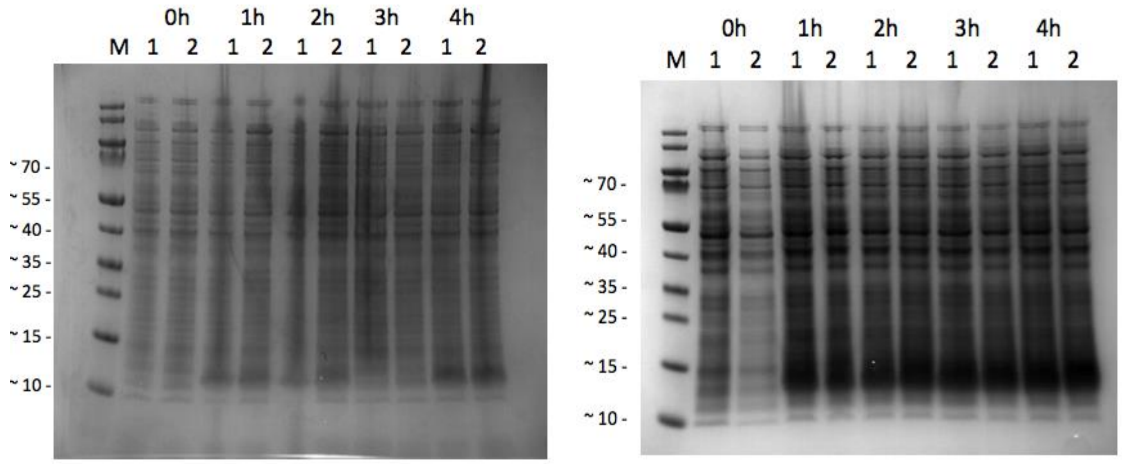

Figure S5. SDS-PAGE gels showing the expression profiles for the 6C/28H/32H and 6H/8H/28C constructs, shown left and right respectively.

Figure S6 shows the chromatograms and SDS-PAGE gels that correspond to the anionic-exchange step of protein purification. For the I6H/N8H/K28C construct, after the first HiTrap anionic-exchange column, there was significant protein present in both the flow-through- and wash-steps, indicated by the red circle on the SDS-PAGE gel shown in figure S6 (centre panel, right column). This could be explained by an insufficient volume of lysis buffer when re-solubilising the cell pellet, leading to a higher conductivity, and lower affinity for the HiTrap column. Therefore, this anionic-exchange step was repeated, ensuring that conductivity was sufficiently reduced by dilution with buffer A and avoiding any potential overloading by using two HiTrap columns in tandem; the chromatogram and SDS-PAGE gel of the repeated anionic-exchange step are shown in the bottom row of figure S6.

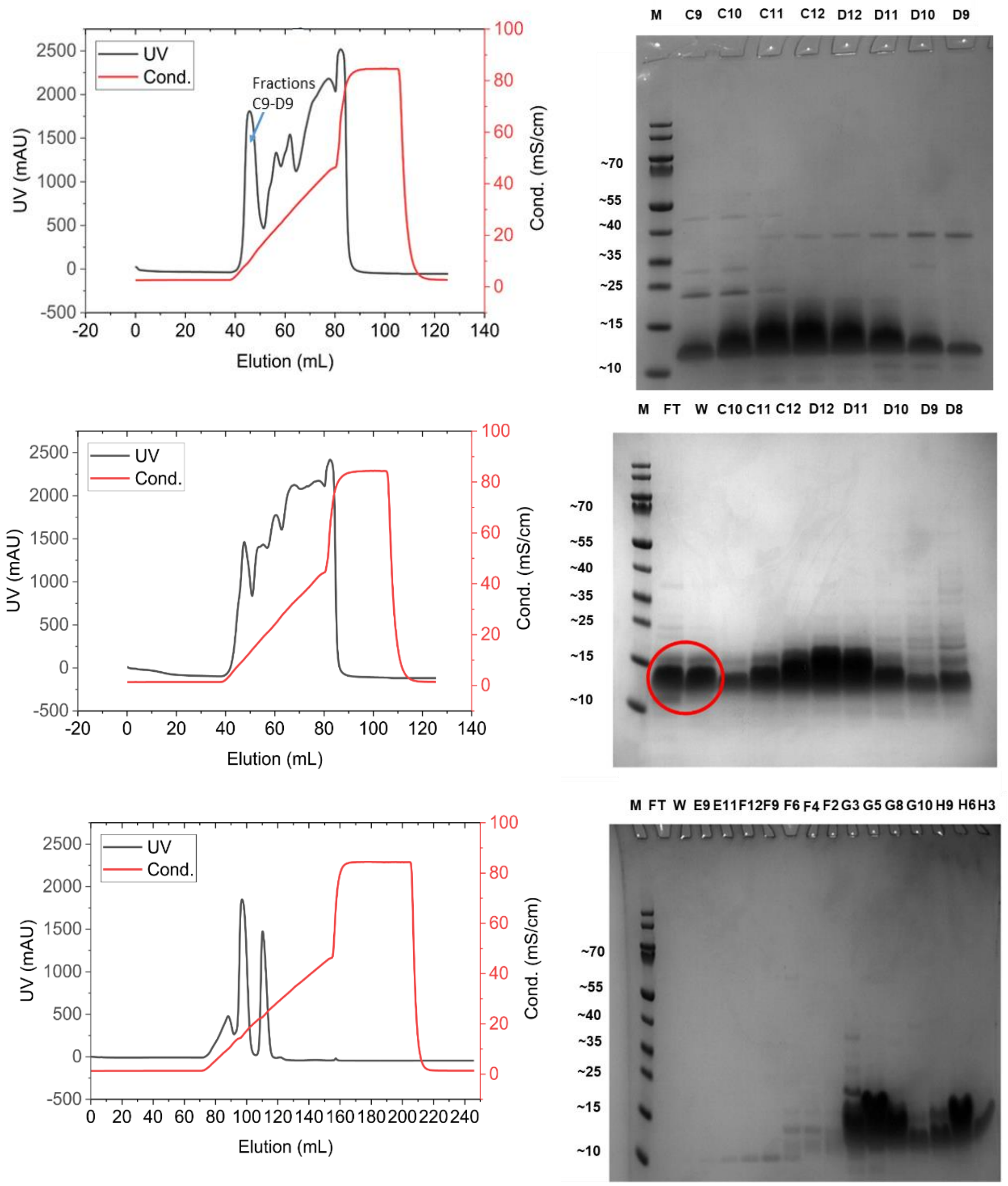

Figure S6. Chromatograms of the HiTrap anionic-exchange columns recorded at 280 nm, with absorbance shown in black and conductivity shown in red, and SDS-PAGE gels assessing fraction purity for the I6C/K28H/Q32H construct in the top row, and for the I6H/N8H/K28C construct in the centre and bottom rows, for the first and second HiTrap columns, respectively.

Figure S7 shows the chromatograms and SDS-PAGE gels that correspond to the size-exclusion chromatography step of protein purification. Particularly, given the low molecular weight of GB1, this proves to be an important step since the lack of any affinity tag on

the constructs means contaminant proteins can co-purify in the first stages of purification; for instance, in the SDS-PAGE gels shown in figure S7. This step also allowed for the removal of unbound spin label, and a more thorough buffer exchange before EPR sample preparation, with higher efficiency than a PD10 column. As can be seen, protein purity is sufficiently high to proceed with use in subsequent experiments, with only a single peak corresponding to GB1, circled in the chromatograms.

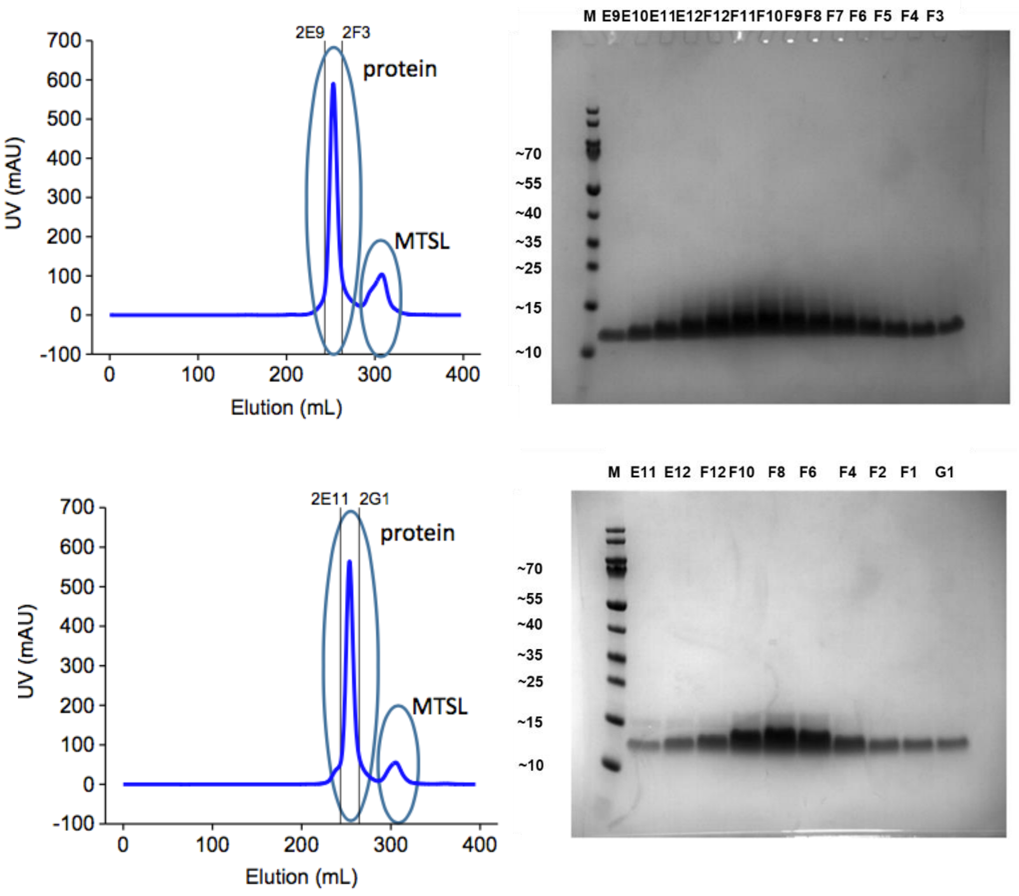

Figure S7. Chromatograms recorded at 280 nm, and SDS-PAGE gels assessing fraction purity for I6C/K28H/Q32H and I6H/N8H/K28C constructs, in the top and bottom rows, respectively.

All pooled fractions were subsequently confirmed to contain pure GB1 *via* mass spectrometry, as shown in figures S9-10 in section 2.3.

2.2 Continuous-Wave EPR Measurements:

The continuous-wave EPR spectra are shown in figure S8 for constructs I6R1/K28H/Q32H and I6H/N8H/K28R1 GB1 and indicate an absence of free-label, lacking high mobility components in the high-field peaks. For the quantification, all samples were acquired for 10 scans; prior to integration steps, data were baseline corrected using cubic polynomial functions, and the double integrals (DI) were calculated using the Bruker™ WIN-EPR software package. The corresponding labelling efficiencies are given in table S1 below and indicate quantitative labelling of both constructs.

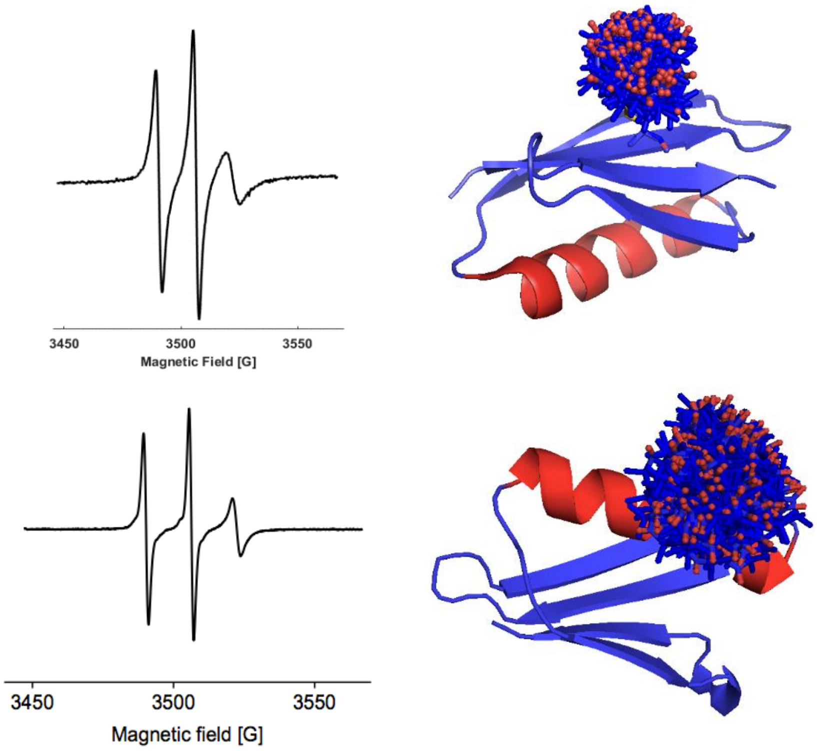

Figure S8. Continuous-wave EPR spectra of I6R1/K28H/Q32H and I6H/N8H/K28R1 constructs in the top and bottom left, respectively. Each construct is shown diagrammatically with the MTSL rotamer library shown at the relevant positions (top and bottom right), respectively.

Sample	Labelling Efficiency (%)
I6R1/K28H/Q32H	95±5
I6H/N8H/K28R1	97±5

Table S1. Labelling efficiencies for each of the GB1 constructs.

2.3 Mass Spectrometry:

Spectra of unlabelled and MTSL-labelled material of 6H/8H/28C and 6C/28H/32H are shown in figures S9-10, in the left and right panels respectively. All samples show high purity, and the predicted masses agree to within ±3 Da of the experimental values, given in table S2. Results also qualitatively suggest a high R1-labelling efficiency, as was confirmed by quantitative analysis of labelling efficiency with continuous-wave EPR, shown in figure S8 of section 2.2.

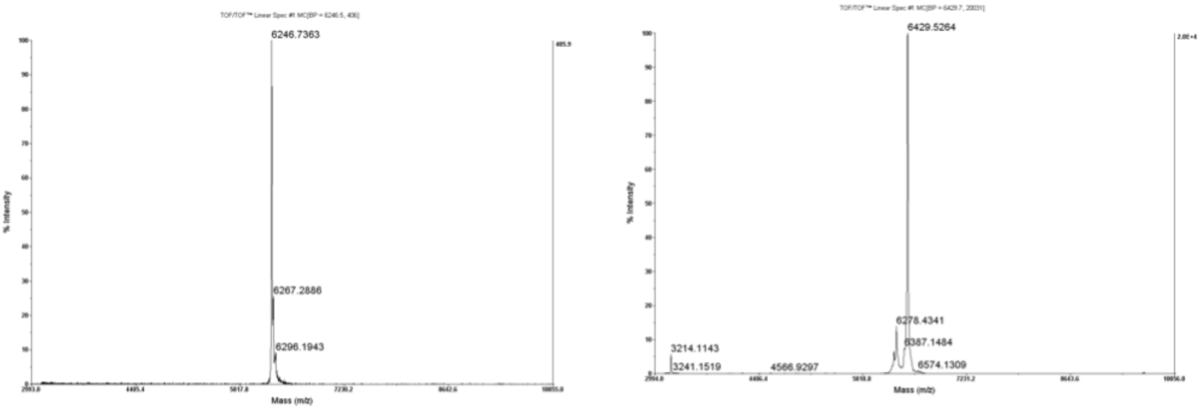

Figure S9. MALDI-TOF mass spectra of 6H/8H/28C and 6H/8H/28R1 shown in the left and right panels respectively.

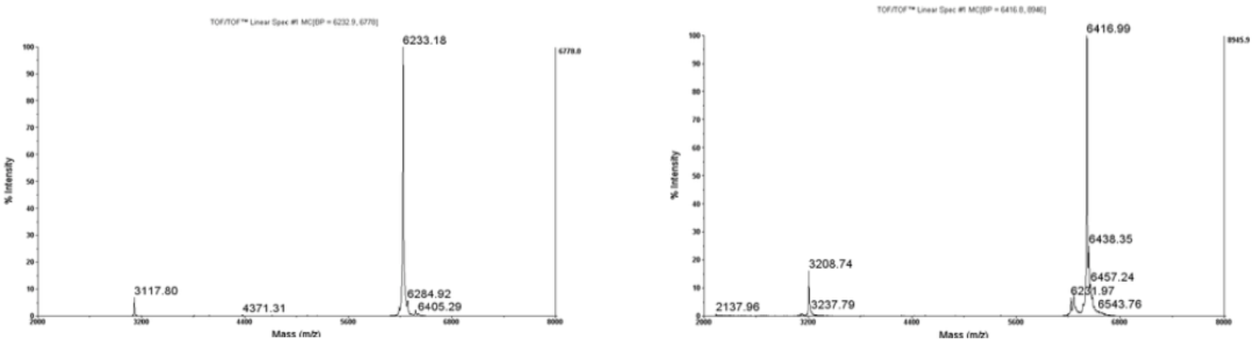

Figure S10. MALDI-TOF mass spectra of 6C/28H/32H and 6R1/28H/32H shown in the left and right panels respectively.

Construct	Predicted Mass [Da]	Observed Mass [Da]
I6H/N8H/K28C	6245	6247
I6H/N8H/K28R1	6432	6430
I6C/K28H/Q32H	6231	6233
I6R1/K28H/Q32H	6416	6417

Table S2. The predicted and observed masses corresponding to the spectra given in figures S9 and S10.

2.4 Cu^{II}-label Concentration Quantification by UV-visible Spectroscopy:

The concentrations of the Cu^{II}-chelate solutions were quantified using UV-visible spectrophotometry. For Cu^{II}-IDA an extinction coefficient (ε) for A_{726nm} of 62 $M^{-1}cm^{-1}$ was used.[13] Solutions of 25.4, 12.7, 5.1 and 2.5 mM Cu^{II}-IDA were prepared for quantification, as described in section 1.3; measurement of the Cu^{II}-IDA dilution-series was repeated 4 times, and spectra are overlaid in the first row of figure S11.

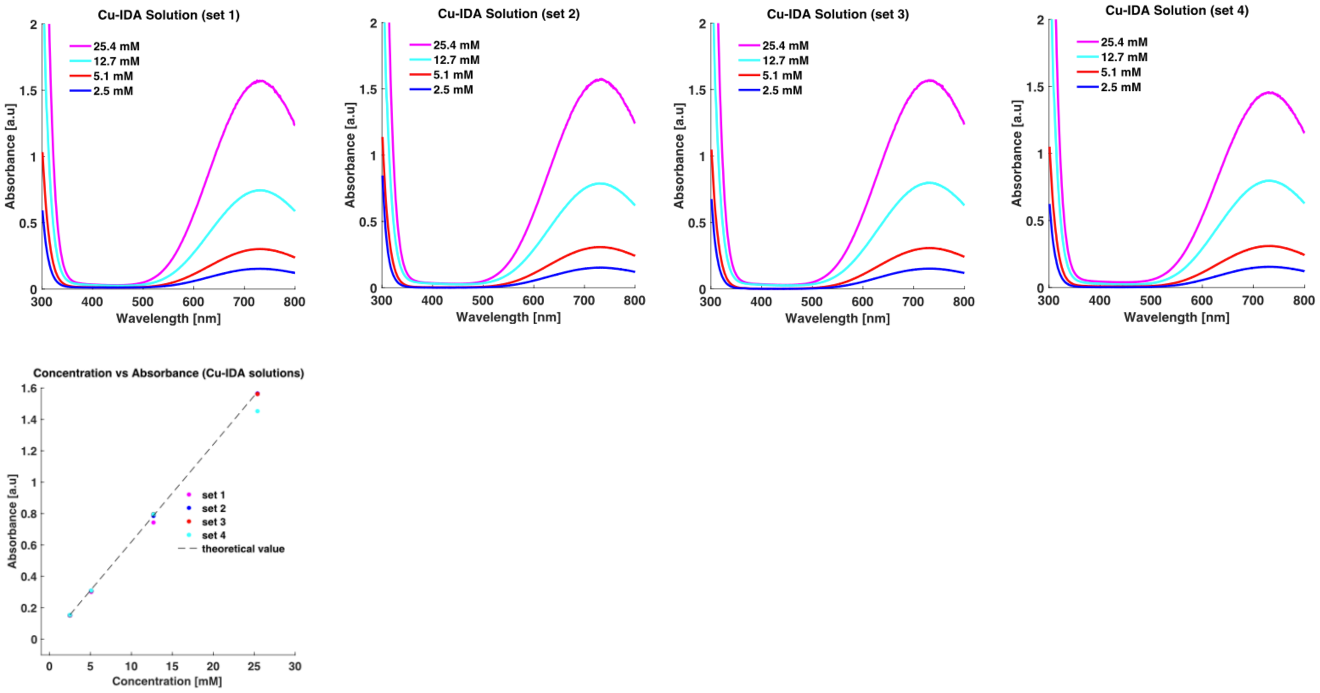

Figure S11. Absorbance spectra recorded for a Cu^{II}-IDA dilution series and repeated in 4 sets. In each set, the 25.4 mM stock solution is shown in magenta, the 2-fold dilution is shown in cyan, the 5-fold dilution is shown in red and the 10-fold dilution is shown in blue.

Sample	Predicted A_{726nm}	Observed A_{726nm}				Concentration [mM]			
Repeat		1	2	3	4	1	2	3	4
25.4 mM Cu^{II}-IDA	1.575	1.565	1.564	1.561	1.452	25.2	25.2	25.2	25.2
12.7 mM Cu^{II}-IDA	0.787	0.743	0.785	0.797	0.796	12.0	12.7	12.9	12.8
5.1 mM Cu^{II}-IDA	0.316	0.301	0.307	0.307	0.309	4.9	5.0	5.0	5.0
2.5 mM Cu^{II}-IDA	0.155	0.152	0.151	0.152	0.154	2.5	2.4	2.5	2.5

Table S3. The predicted and observed absorbance for the initial Cu^{II}-IDA dilution series, along with the calculated Cu^{II}-IDA concentrations, taken from the spectra shown in figure S11. The predicted absorbance is estimated using the literature extinction coefficient of 62 $M^{-1}cm^{-1}$.

To confirm the reported extinction coefficient, A_{726nm} was plotted as a function of Cu^{II}-IDA concentration and found to be approximately linear, shown in the first panel of the second row of figure S11. Calculated Cu^{II}-IDA concentrations are given above in table S3. A stock solution of Cu^{II}-IDA was freeze-dried and re-dissolved before repeating the dilution series as above, to check no change in concentration owing to the process; results are shown in figure S12 and indicate that freeze-drying is lossless as there is no change to Cu^{II}-IDA concentration; and calculated concentrations are given in table S4.

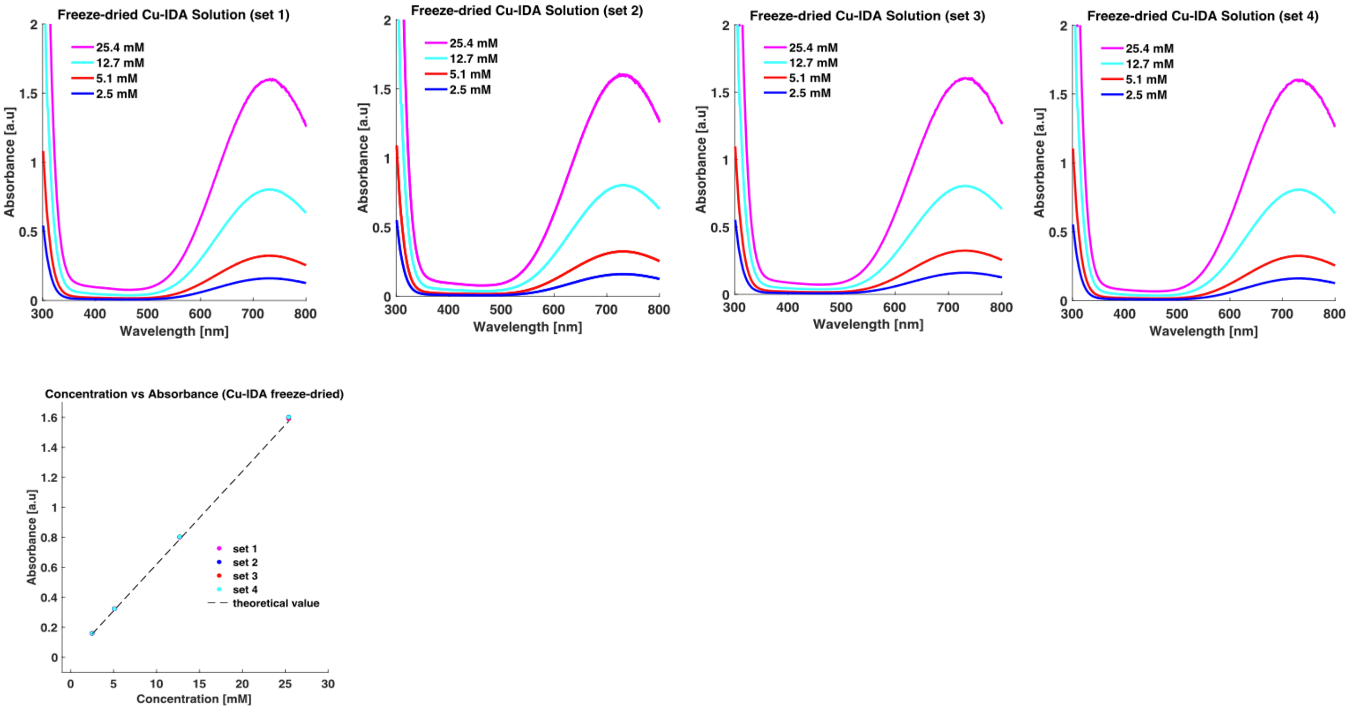

Figure S12. Absorbance spectra recorded for the freeze-dried Cu^{II}-IDA dilution series and repeated in 4 sets; the colour scheme is consistent with that used in figure S11.

Sample	Predicted A_{726nm}	Observed A_{726nm}				Concentration [mM]			
Repeat		1	2	3	4	1	2	3	4
25.4 mM Cu^{II}-IDA	1.575	1.591	1.603	1.595	1.602	25.7	25.9	25.7	25.8
12.7 mM Cu^{II}-IDA	0.787	0.802	0.802	0.803	0.804	12.9	12.9	13.0	13.0
5.1 mM Cu^{II}-IDA	0.316	0.323	0.324	0.325	0.324	5.2	5.2	5.2	5.2
2.5 mM Cu^{II}-IDA	0.155	0.160	0.160	0.161	0.161	2.6	2.6	2.6	2.6

Table S4. The predicted and observed absorbance for the freeze-dried Cu^{II}-IDA dilution series, along with the calculated Cu^{II}-IDA concentrations, taken from the spectra shown in figure S12.

For the quantification of Cu^{II}-NTA concentration, a stock solution of nominally 10 mM was prepared as described in section 1.3; 7.5, 5.0, 2.5 and 1.0 mM solutions were prepared as a dilution series. No extinction coefficient could be found for Cu^{II}-NTA in published literature, so instead ε was calculated empirically using A_{800nm}, and determined to be 63 $M^{-1}cm^{-1}$. Experimental spectra were recorded for two independent preparations, shown in panels of figure S13, along with the determination of the extinction coefficient at A_{768nm}.

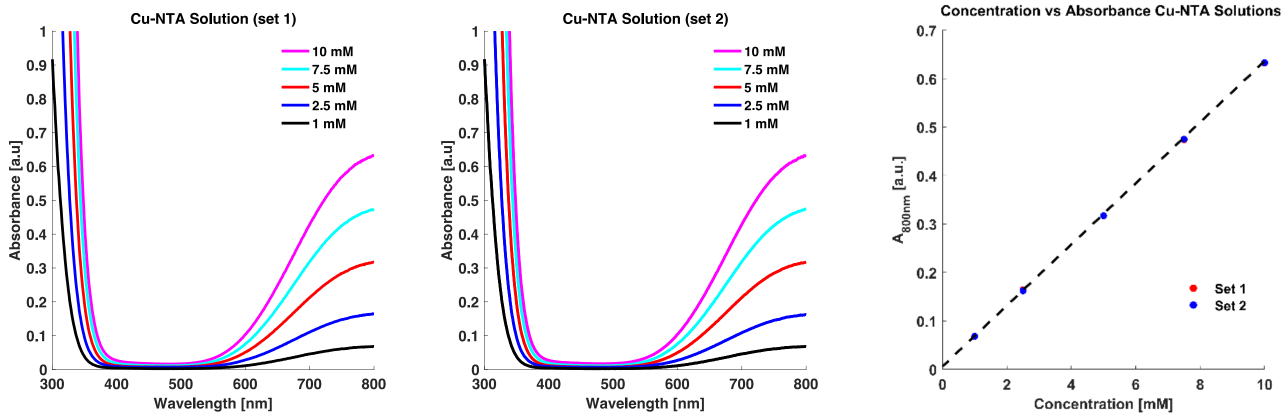

Figure S13. Absorbance spectra recorded for the Cu^{II}-NTA dilution series and repeated in 2 sets; the colour scheme is consistent with that used in figure S11, with the addition of 1 mM Cu^{II}-NTA shown in black. The theoretical value shown in dotted black in the right-most panel is calculated using an extinction coefficient of 63 $M^{-1}cm^{-1}$.

Sample	Observed A_{800nm}	
Repeat	1	2
10 mM Cu^{II}-NTA	0.632	0.633
7.5 mM Cu^{II}-NTA	0.474	0.475
5.0 mM Cu^{II}-NTA	0.317	0.317
2.5 mM Cu^{II}-NTA	0.164	0.162
1.0 mM Cu^{II}-NTA	0.068	0.068

Table S5. The observed absorbance for each of the Cu^{II}-NTA dilution series taken from the spectra shown in figure S13.

2.5 Circular Dichroism Spectra and Thermal Denaturation Assays:

The far UV CD spectra for 6H/8H/28C and 6C/28H/32H GB1 are shown in figure S14, in presence and absence of 3 equivalents Cu^{II}-IDA, in blue and red respectively. Since GB1 contains both α-helical and β-sheet secondary structural elements, components of both should be present in the corresponding spectra; α-helices typically have double-lobed minima in the region 205-225 nm, while β-sheets give rise to shallower, singularly-lobed minima around 220 nm.[14] In all cases, samples have a singular minimum ~220 nm and indication of a turning point around 200 nm (clearly visible for the 6C/28H/32H GB1 w/o Cu^{II}-IDA sample), which is typical of β-sheet motifs.

Interestingly, results suggest that binding of the Cu^{II}-IDA to the helical double-histidine motif at positions 28H/32H has a stabilizing effect on the structure of the helix indicated by the increased magnitude of the global minimum at ~220 nm, and the sharpening of the trough; this has also been reported in previous literature1] and furthermore supports the hypothesis that a binding event at the helix-site incurs an entropic cost. On the other-hand, for the β-sheet double-histidine site, the effect of Cu^{II}-IDA binding on the protein secondary structure appears to be almost negligible; this would be consistent with a sterically restricted binding site, with little plasticity – as is predicted for the sheet-site. All data agrees with circular dichroism spectra previously published[1] on both WT and 6H/8H/28H/32H GB1, suggesting there is little structural perturbation introduced by the mutations.

Thermal denaturation studies for each construct at 220 nm are shown in the main body of the left panel of figure S15, while the melting curves at 260 nm for each construct are shown in the inset. In each case the two-state unfolding fits are shown in black; residuals are shown for each fit in the right panel of figure S15, with the corresponding melting temperatures T_M given in table S6.

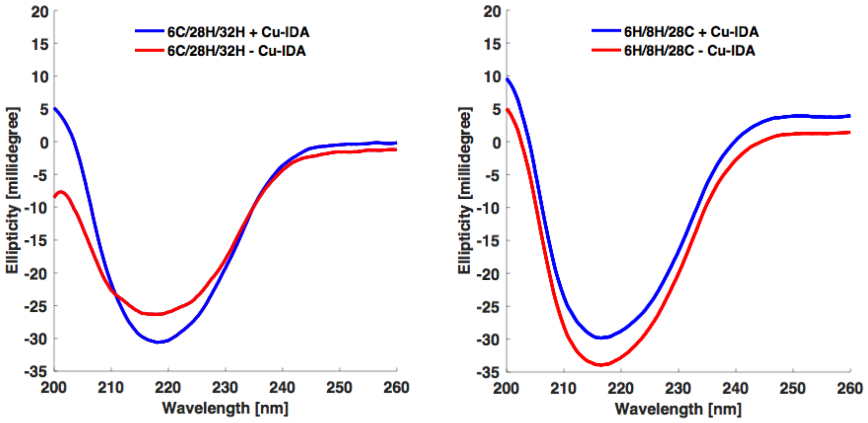

Figure S14: Circular dichroism spectra for 6C/28H/32H and 6H/8H/28C GB1 shown in left and right panels respectively, in presence and absence of 3 equivalents of Cu^{II}-IDA.

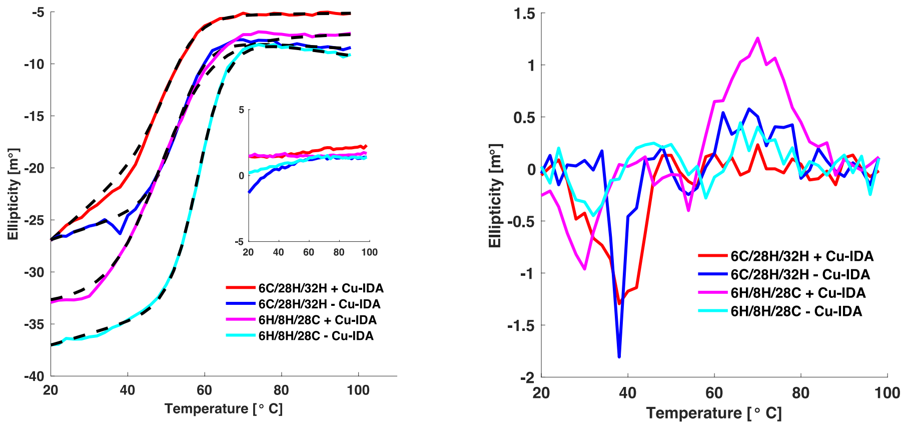

Figure S15: Thermal denaturation curves of GB1 constructs in presence and absence of 3 equivalents Cu^{II}-IDA, shown in the left panel. T_M fits are shown in dotted black, with 6H/8H/28C ± Cu^{II}-IDA shown in magenta and cyan respectively, and 6C/28H/32H ± Cu^{II}-IDA shown in red and blue, respectively. In the right panel, the residuals of each of the fits to the raw data are given, with the same colour scheme.

Sample Condition	T_M [ºC]
6H/8H/28C – Cu^{II}-IDA	55
6H/8H/28C + Cu^{II}-IDA	45
6C/28H/32H – Cu^{II}-IDA	51
6C/28H/32H + Cu^{II}-IDA	48

Table S6: Melting temperatures for each of the sample conditions, extracted from the raw data shown in the left panel of figure S15.

The thermal melting curves suggest that the binding of Cu^{II}-IDA at both positions has a destabilising effect, although the magnitude of this is vastly different between the two constructs. In the case of 6C/28H/32H the melting temperature is predicted to be 51 ºC and 48 ºC in the absence and presence of Cu^{II}-IDA, respectively. Interestingly, the 6H/8H/28C construct shows the highest stability in absence of Cu^{II}-IDA, with a melting temperature predicted to be 55 ºC; but upon Cu^{II}-IDA addition this reduces to 45 ºC, the lowest melting temperature of all conditions, suggesting the addition of Cu^{II}-IDA to the helix double-histidine motif (I6C/K28H/Q32H) is less de-stabilising than corresponding addition to the sheet double-histidine motif (I6H/N8H/K28C).

2.6 Isothermal Titration Calorimetry:

For measurement of the I6R1/K28H/Q32H construct, 1000 µM Cu^{II}-NTA ligand, and 2000 µM Cu^{II}-IDA ligand, respectively, were titrated into 75 µM protein solutions, all in filtered buffer C. The resulting isotherms, raw data and fits are shown in figure S16. The calculated K_D values based on these fits are 5.0 ± 0.3 and 27.0 ± 1.8 µM for Cu^{II}-NTA and Cu^{II}-IDA respectively, which follows the expected trend for binding to a double-histidine motif on an α-helix, albeit with an affinity two orders of magnitude greater than previously reported.[15] For measurement of the I6H/N8H/K28R1 construct, 2000 µM Cu^{II}-NTA ligand, and 1000 µM Cu^{II}-IDA ligand, respectively, were titrated into 75 µM protein solutions, all in filtered buffer C; as above. The resulting isotherms, raw data and fits are shown in figure S17. The determined K_D values for Cu^{II}-NTA and Cu^{II}-IDA are 42 ± 2 µM and 7 ± 1 µM, respectively; this is consistent with a lower affinity posited previously.[1]

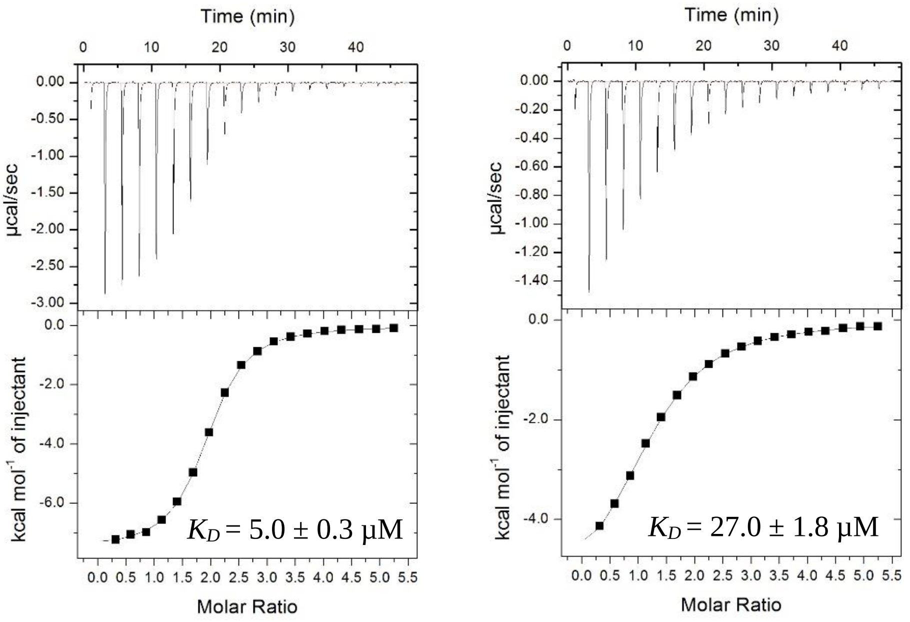

Figure S16. Isothermal titration calorimetry data for the I6R1/K28H/Q32H construct, titrated against Cu^{II}-NTA and Cu^{II}-IDA, shown in the left and right panels respectively, with the raw ITC traces shown at the top, and the binding isotherm at the bottom in each case

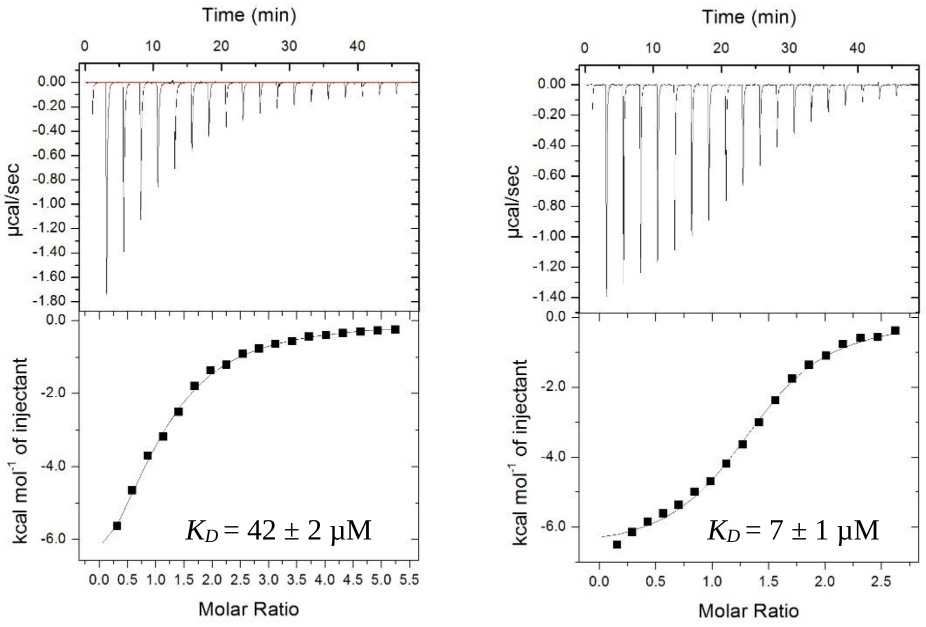

Figure S17. Isothermal titration calorimetry data for the I6H/N8H/K28R1 construct, titrated against Cu^{II}-NTA and Cu^{II}-IDA and shown in the left and right panels respectively, with the raw ITC traces shown at the top, and the binding isotherm at the bottom in each case.

From the ITC data shown above, the coordination of each Cu^{II}-chelate to dH-motifs is shown to be an exothermic reaction, and the empirical enthalpy changes calculated for each construct/chelator permutation are given in table S7 below.

The integrated form of van't Hoff equation is given as:

$$\ln\frac{K_2}{K_1} = \frac{-\Delta H^{\emptyset}}{R}\left(\frac{1}{T_2} - \frac{1}{T_1}\right) \quad (11)$$

It is apparent that for an exothermic reaction, as the temperature is decreased the affinity of binding increases, and the RIDME data shown in figure S26 suggest that K_D values estimated for both Cu^{II}-NTA and Cu^{II}-IDA for both constructs are considerably lower, than the value from ITC at room temperature. This is reconciled by extrapolation of K_D values to low temperature and minimising the RMSD

between the four RIDME-determined K_Ds and the ITC extrapolation as a function of temperature. Interestingly, the deviation is minimal at 239 K. This means that the RIDME binding constant is reflective of the ITC derived thermodynamic parameters at 239 K. Coincidentally, this is very close to the melting point of a 1:1 water-ethylene glycol mixture at about 235 K.[18] It appears that below this temperature the decreasing diffusional motion and increasing viscosity allow no further equilibration and the ions will be trapped in the solid matrix whether bound or unbound.

Therefore, the observed numerical difference between the ITC-determined and RIDME determined K_D values seems to manifest because of measurement under two distinct temperature regimes: room-temperature for the ITC data, and at the freezing point for the RIDME pseudo-titration data. Furthermore, we can conclude that due to the exothermic nature of binding, this leads to much stronger binding under PDEPR conditions, as suggested by data shown in figure S26.This is discussed with respect to the RIDME pseudo-titrations in section 2.13 of the SI.

ITC sample	Empirical ΔH [kcal.mol^{-1}]	Empirical ΔS [cal.mol^{-1}.deg^{-1}]	Wiseman Factor (*c*)
75 µM I6R1/K28H/Q32H GB1 + Cu^{II}-NTA	-7.54 ± 0.02	-1.0	15.0
75 µM I6R1/K28H/Q32H GB1 + Cu^{II}-IDA	-5.75 ± 0.05	-1.6	2.8
75 µM I6H/N8H/K28R1 GB1 + Cu^{II}-NTA	-9.68 ± 0.52	-12.5	1.8
75 µM I6H/N8H/K28R1 GB1 + Cu^{II}-IDA	-3.37 ± 0.05	10.8	10.7

Table S7: The empirical ΔH and ΔS values calculated from each ITC measurement, and the associated Wiseman factor *c*.

2.7 *Inversion Recovery:*

The raw inversion recovery traces and the corresponding mono-exponential and bi-exponential fits are shown for the 0.5 µM pseudo-titration series, in figure S18. Inversion recovery traces for the 25 and 75 µM pseudo-titrations will be deposited as part of the underpinning data, but the estimated T_1 values from the mono- and bi-exponential fits are given in tables S9-12 for I6H/N8H/K28R1 and I6R1/K28H/Q32H in presence of Cu^{II}-NTA and Cu^{II}-IDA, respectively. All mono-exponential functions fit the raw data well and do not appear to show significant contribution from spectral diffusion to the inversion recovery measurement that could lead to under-estimating the T_1. Spectral diffusion can manifest as a fast component of the inversion recovery data, and subsequently cause mono-exponential fits to under-represent longitudinal relaxation time.

Although not performed during the present work spectral diffusion can be reduced in future measurements of T_1 by instead using the saturation recovery (SR) experiment; a train of inversion pulses will saturate both resonant electron spin transitions within the bandwidth of the detection pulse, and the transitions which can undergo magnetization exchange with this region of the spectrum, otherwise contributing to spectral diffusion. The T_1 values estimated from the mono- and bi-exponential fits of the raw data shown in figure S18, along with the 1/e time (the time taken for ~63% of the electron spin magnetisation to return to thermal equilibrium) are given in table S8. The relative contributions of the two decay time constants in the bi-exponential fits are given as the parameter *b* in equation 2 in section 1.5.

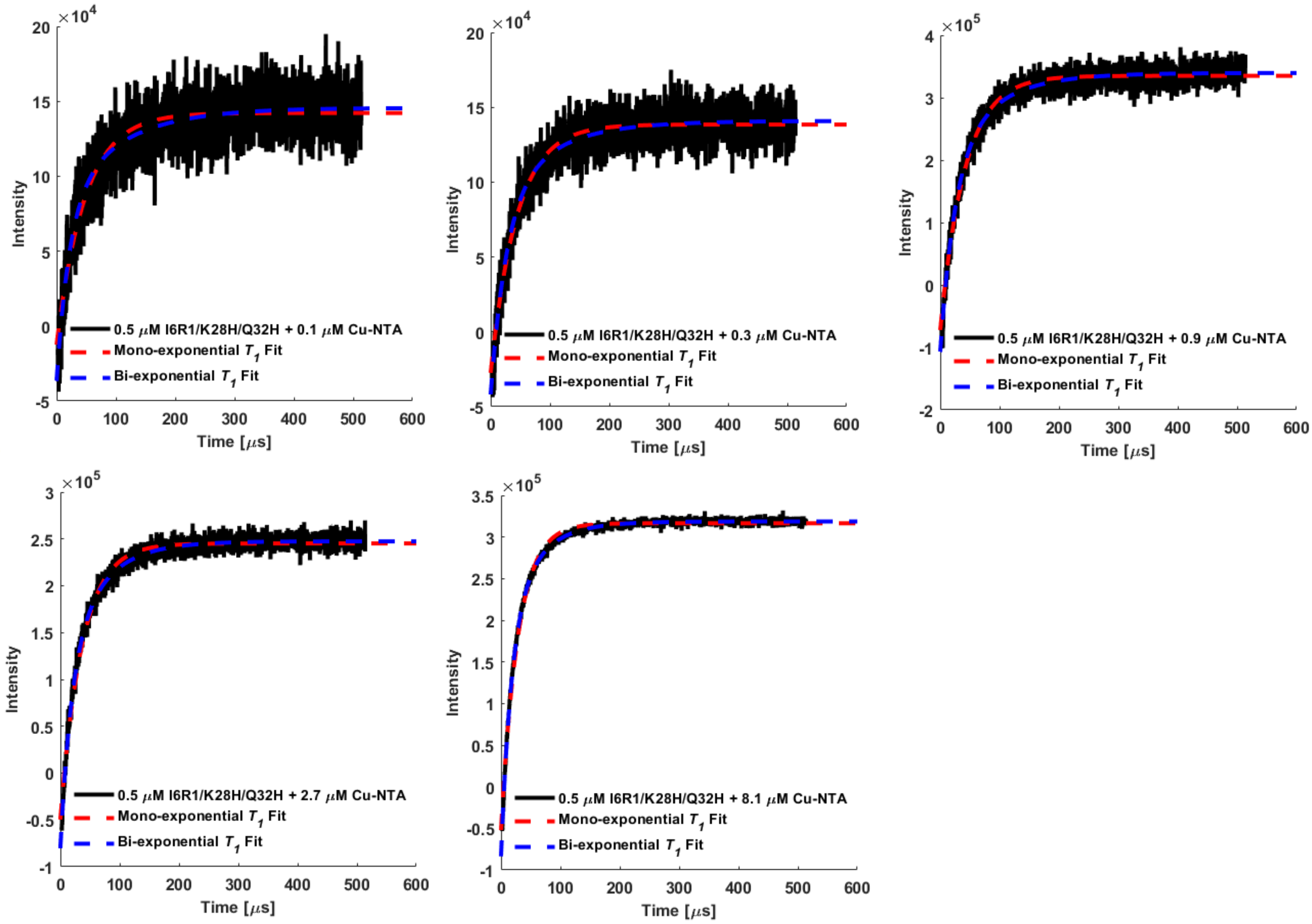

Figure S18. Inversion recovery data for 0.5 μM I6R1/K28H/Q32H, in presence of 0.1, 0.3, 0.9, 2.7 and 8.1 μM Cu^{II}-NTA, shown left-to-right, and top-to-bottom, respectively. The experimental data is shown in black, with the mono-exponential and bi-exponential fits shown as red and blue dotted lines respectively.

Sample	Mono-exponential T_1 [μs]	Bi-exponential T_{1A} / T_{1B} [μs]	Relative Contributions	1/e time [μs]
I6R1/K28H/Q32H + 0.1 μM Cu^{II}-NTA	47.8	21.7 / 98.9	0.63 : 0.37	63.4
I6R1/K28H/Q32H + 0.3 μM Cu^{II}-NTA	44.3	26.6 / 92.0	0.68 : 0.32	59.4
I6R1/K28H/Q32H + 0.9 μM Cu^{II}-NTA	41.5	24.9 / 81.1	0.67 : 0.33	56.6
I6R1/K28H/Q32H + 2.7 μM Cu^{II}-NTA	36.6	18.9 / 60.6	0.57 : 0.43	48.8
I6R1/K28H/Q32H + 8.1 μM Cu^{II}-NTA	28.6	17.9 / 54.3	0.69 : 0.31	43.8

Table S8: Mono- and bi-exponential T1 estimates, and 1/e times for each of the RIDME 0.5 μM I6R1/K28H/Q32H pseudo-titration samples, from the inversion recovery traces shown in figure S18 above.

Sample	Mono-exponential T_1 [μs]	Bi-exponential T_1 [μs]	1/e time [μs]
I6H/N8H/K28R1 + 10 μM Cu^{II}-NTA	46.3	31.7 (0.67) / 80.1 (0.33)	56.4
I6H/N8H/K28R1 + 20 μM Cu^{II}-NTA	44.5	30.5 (0.65) / 74.1 (0.35)	55.0
I6H/N8H/K28R1 + 40 μM Cu^{II}-NTA	38.9	25.5 (0.68) / 70.9 (0.32)	47.0
I6H/N8H/K28R1 + 90 μM Cu^{II}-NTA	30.9	18.9 (0.66) / 55.5 (0.34)	35.8
I6H/N8H/K28R1 + 600 μM Cu^{II}-NTA	25.5	16.6 (0.72) / 49.9 (0.28)	29.6

Table S9. Mono- and bi-exponential T_1 estimates, and 1/e time for each sample of the 25 μM I6H/N8H/K28R1 + Cu^{II}-NTA RIDME pseudo-titration series.

Sample	Mono-exponential T_1 [μs]	Bi-exponential T_1 [μs]	1/e time [μs]
I6H/N8H/K28R1 + 10 μM Cu^{II}-IDA	54.1	36.0 (0.64) / 93.8 (0.36)	63.4
I6H/N8H/K28R1 + 20 μM Cu^{II}-IDA	48.7	32.3 (0.64) / 82.1 (0.36)	57.8
I6H/N8H/K28R1 + 35 μM Cu^{II}-IDA	46.3	30.3 (0.66) / 82.9 (0.34)	55.8
I6H/N8H/K28R1 + 75 μM Cu^{II}-IDA	39.3	26.0 (0.67) / 69.4 (0.33)	49.0
I6H/N8H/K28R1 + 450 μM Cu^{II}-IDA	36.2	24.2 (0.68) / 64.8 (0.32)	43.6

Table S10. Mono- and bi-exponential T_1 estimates, and 1/e time for each sample of the 25 μM I6H/N8H/K28R1 + Cu^{II}-IDA RIDME pseudo-titration series.

Sample	Mono-exponential T_1 [μs]	Bi-exponential T_1 [μs]	1/e time [μs]
I6R1/K28H/Q32H + 15 μM Cu^{II}-NTA	51.6	35.5 (0.65) / 86.0 (0.35)	62.8
I6R1/K28H/Q32H + 30 μM Cu^{II}-NTA	48.7	32.3 (0.66) / 85.4 (0.34)	59.2
I6R1/K28H/Q32H + 60 μM Cu^{II}-NTA	36.5	21.3 (0.62) / 63.2 (0.38)	42.0
I6R1/K28H/Q32H + 135 μM Cu^{II}-NTA	29.1	16.9 (0.63) / 50.1 (0.37)	33.0
I6R1/K28H/Q32H + 960 μM Cu^{II}-NTA	25.1	15.7 (0.68) / 46.0 (0.32)	29.0

Table S11. Mono- and bi-exponential T_1 estimates, and 1/e time for each sample of the 25 μM I6R1/K28H/Q32H + Cu^{II}-NTA RIDME pseudo-titration series.

Sample	Mono-exponential T_1 [μs]	Bi-exponential T_1 [μs]	1/e time [μs]
I6R1/K28H/Q32H + 45 μM Cu^{II}-IDA	59.7	42.0 (0.66) / 102.9 (0.34)	73.4
I6R1/K28H/Q32H + 100 μM Cu^{II}-IDA	49.9	32.1 (0.62) / 82.5 (0.38)	61.6
I6R1/K28H/Q32H + 185 μM Cu^{II}-IDA	44.2	28.1 (0.65) / 79.1 (0.35)	52.8
I6R1/K28H/Q32H + 350 μM Cu^{II}-IDA	40.7	26.1 (0.67) / 74.8 (0.33)	48.0
I6R1/K28H/Q32H + 600 μM Cu^{II}-IDA	39.5	25.6 (0.66) / 70.1 (0.34)	46.8
I6R1/K28H/Q32H + 1750 μM Cu^{II}-IDA	36.4	24.1 (0.70) / 68.2 (0.30)	45.2

Table S12: Mono- and bi-exponential T_1 estimates, and 1/e time for each sample of the 75 μM I6R1/K28H/Q32H + Cu^{II}-IDA RIDME pseudo-titration series.

It was also necessary to perform inversion-recovery measurements at different temperatures to allow estimating the maximum sensitivity as a function of temperature for both Cu^{II}-nitroxide RIDME, and Cu^{II}-Cu^{II} RIDME and PELDOR, discussed in section 2.11. Therefore inversion recovery measurements were performed on the maximum of the Cu^{II}-IDA spectrum (and the R1 nitroxide spectrum for the I6H/N8H/K28R1 construct) in the temperature range 10-50 K on samples of 75 μM I6H/N8H/K28H/Q32H GB1 in presence of 250 μM Cu^{II}-IDA, and 25 μM I6H/N8H/K28R1 GB1 in presence of 100 μM Cu^{II}-IDA. The raw inversion recovery traces and corresponding mono-exponential and bi-exponential fits are shown in figures S19-S21 respectively, and the estimates of T_1 are given in tables S13-S15.

I6H/N8H/K28H/Q32H GB1:

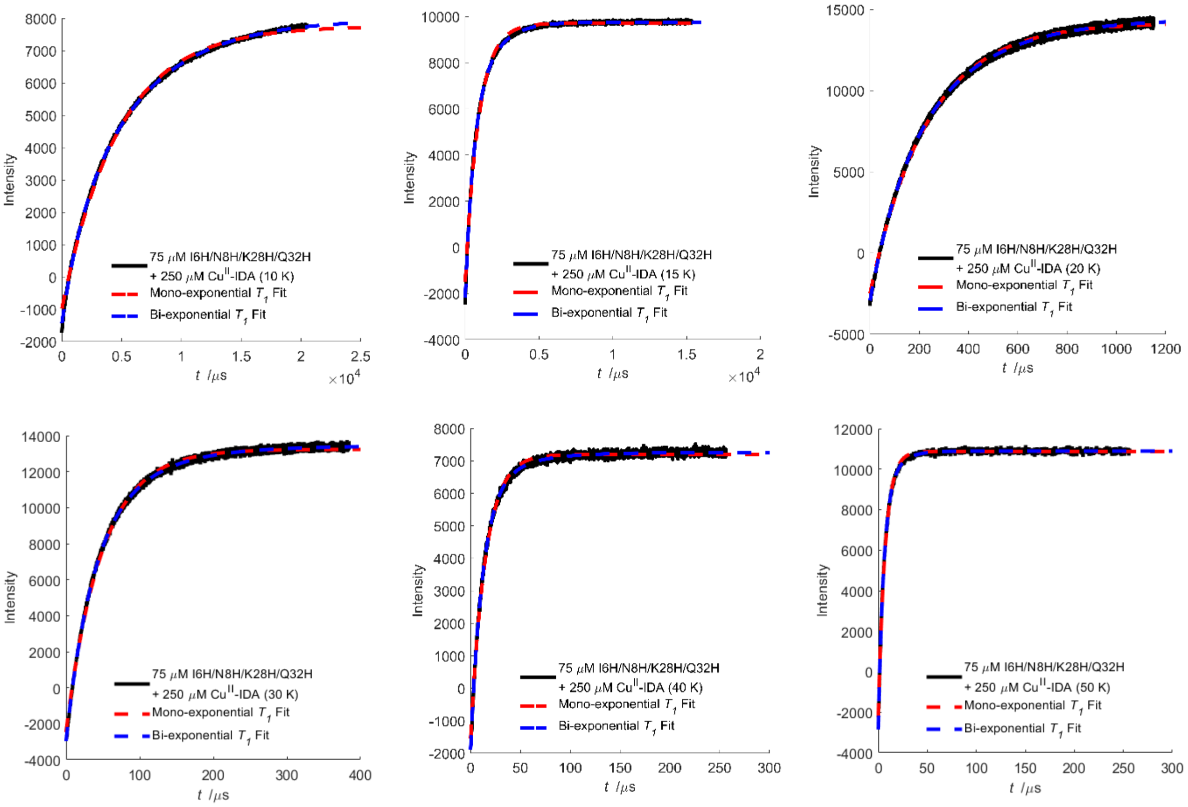

Figure S19. Inversion recovery traces for 75 μM I6H/N8H/K28H/Q32H GB1 + 250 μM Cu^{II}-IDA at various temperatures shown in black, with mono- and bi-exponential fits shown in red and blue, respectively. The corresponding estimates for T_1 are given in table S13 below.

Temperature [K]	Mono-exponential T_1 [μs]	Bi-exponential T_1 [μs]	1/e time [μs]
10	4800	1500 (0.23) / 6130 (0.77)	5201
20	234.0	134.3 (0.44) / 334.3 (0.56)	301.0
30	52.8	31.0 (0.66) / 97.8 (0.34)	67.6
40	15.6	11.2 (0.68) / 25.3 (0.32)	19.6
50	7.6	5.6 (0.81) / 15.6 (0.19)	8.9

Table S13: Mono- and bi-exponential T_1 estimates, and 1/e time for each sample of the 75 μM I6H/N8H/K28H/Q32H GB1 + 250 μM Cu^{II}-IDA temperature series.

I6H/N8H/K28R1 GB1 (Cu^{II}-IDA):

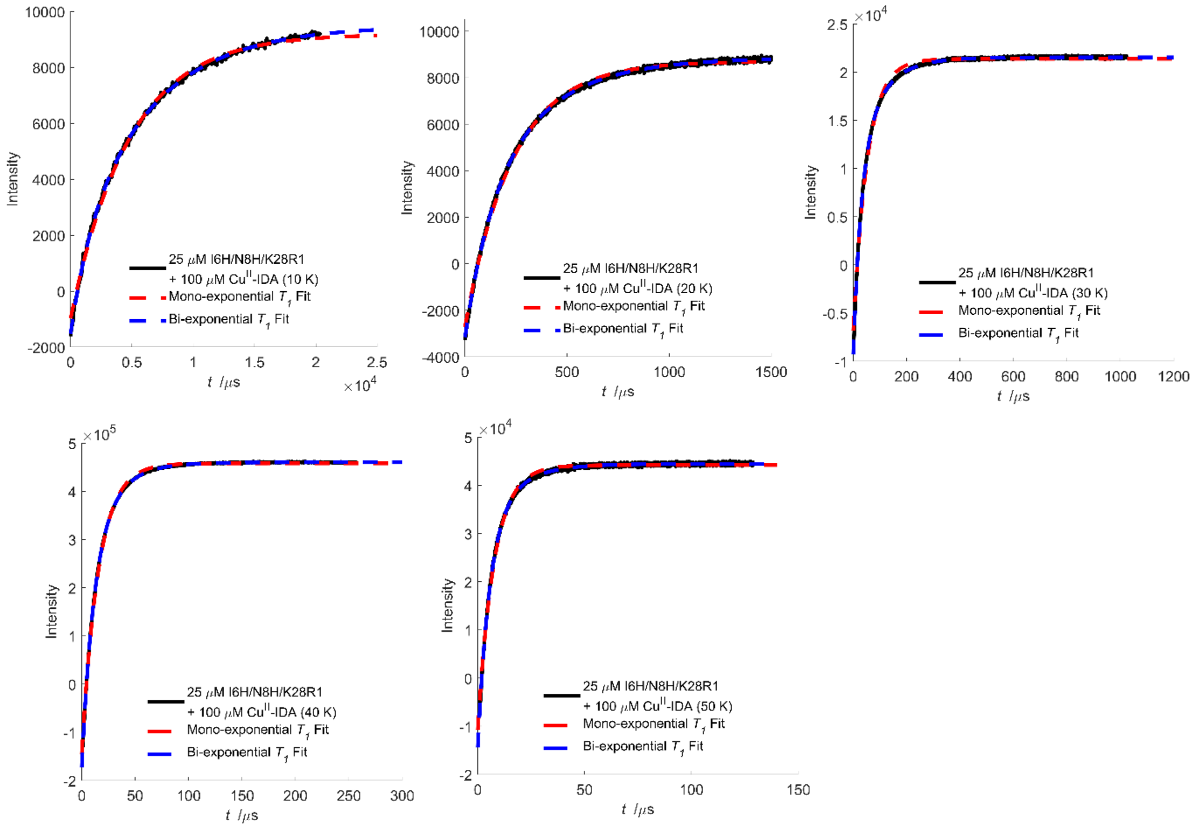

Figure S20. Inversion recovery traces of Cu^{II}-IDA for 25 μM I6H/N8H/K28R1 GB1 + 100 μM Cu^{II}-IDA at various temperatures shown in black, with mono- and bi-exponential fits shown in red and blue, respectively. The corresponding estimates for T_1 are given in table S14 below.

Temperature [K]	Mono-exponential T_1 [μs]	Bi-exponential T_1 [μs]	1/e time [μs]
10	4769	1510 (0.22) / 5.969 (0.78)	5601
15	912.1	521.8 (0.56) / 1441 (0.44)	996.5
20	232.1	128.7 (0.34) / 302.7 (0.66)	256.6
30	48.3	33.9 (0.63) / 79.6 (0.37)	58.3
40	14.8	12.0 (0.86) / 39.5 (0.14)	18.1
50	7.0	5.4 (0.80) / 14.2 (0.20)	8.2

Table S14: Mono- and bi-exponential T_1 estimates, and 1/e time for Cu^{II}-IDA of the 25 μM I6H/N8H/K28R1 GB1 + 100 μM Cu^{II}-IDA temperature series.

I6H/N8H/K28R1 GB1 (R1 nitroxide):

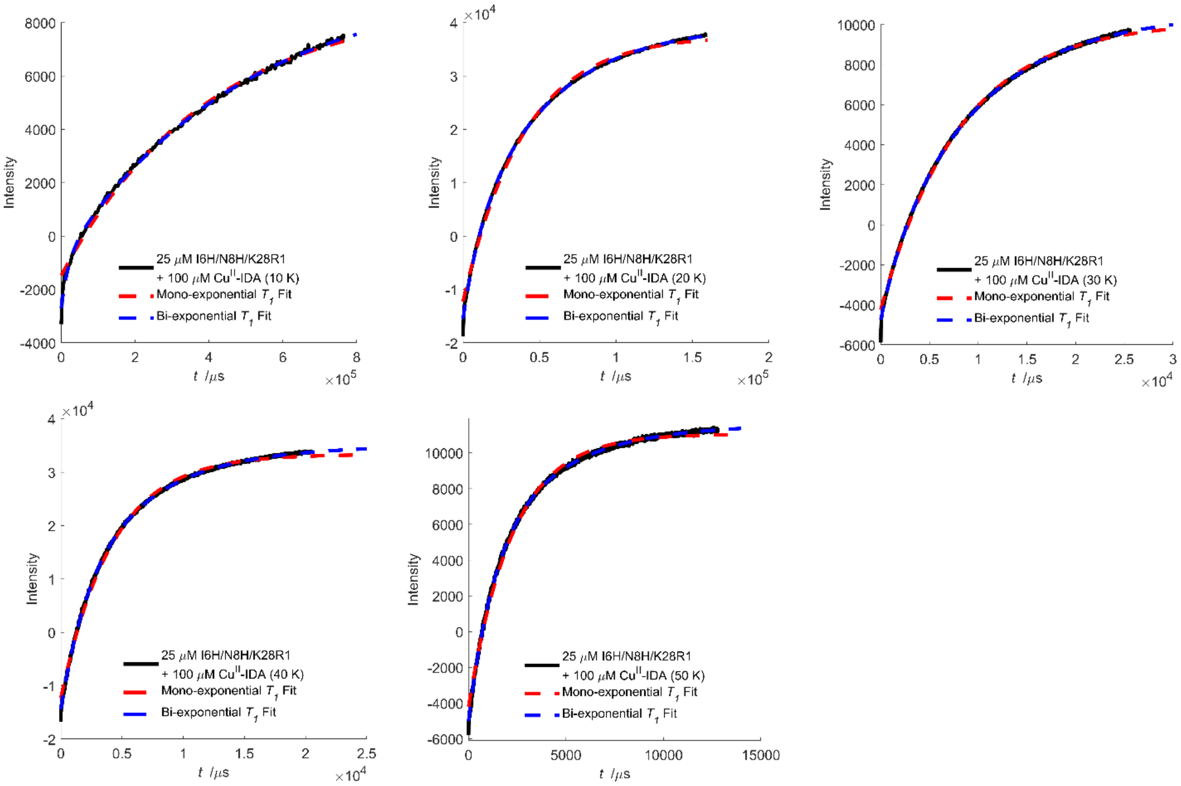

Figure S21. Inversion recovery traces of R1 nitroxide for 25 μM I6H/N8H/K28R1 GB1 + 100 μM Cu^{II}-IDA at various temperatures shown in black, with mono- and bi-exponential fits shown in red and blue, respectively. The corresponding estimates for T_1 are given in table S15 below.

Temperature [K]	Mono-exponential T_1 [ms]	Bi-exponential T_1 [ms]	1/e time [ms]
10	397	196 (0.14) / 486 (0.86)	387
20	39.8	11.7 (0.25) / 53.5 (0.75)	52.0
30	8.1	2.1 (0.13) / 9.6 (0.87)	10.4
40	4.2	2.1 (0.42) / 6.5 (0.58)	5.7
50	2.2	1.2 (0.52) / 3.9 (0.48)	3.1

Table S15: Mono- and bi-exponential T_1 estimates, and 1/e time for R1 nitroxide of the 25 μM I6H/N8H/K28R1 GB1 + 100 μM Cu^{II}-IDA temperature series.

2.8 Electron-spin Echo Decay Measurements:

To allow estimation of sensitivity as a function of temperature for both Cu^{II}-nitroxide RIDME, and Cu^{II}-Cu^{II} RIDME and PELDOR, discussed in section 2.11, it was necessary to estimate the transverse dephasing times T_m for both Cu^{II}-IDA and nitroxide. Therefore electron spin echo decay measurements were performed on the maximum of the Cu^{II}-IDA spectrum (and the R1 nitroxide spectrum for the I6H/N8H/K28R1 construct) in the temperature range 10-50 K on samples of 75 μM I6H/N8H/K28H/Q32H GB1 in presence of 250 μM Cu^{II}-IDA, and 25 μM I6H/N8H/K28R1 GB1 in presence of 100 μM Cu^{II}-IDA. The raw electron spin echo decay traces and corresponding stretched exponential fits are shown in figures S22-S24 respectively, and the estimates of T_m are given in tables S16-S18, fitted according to equation 3 in section 1.6.

I6H/N8H/K28H/Q32H GB1:

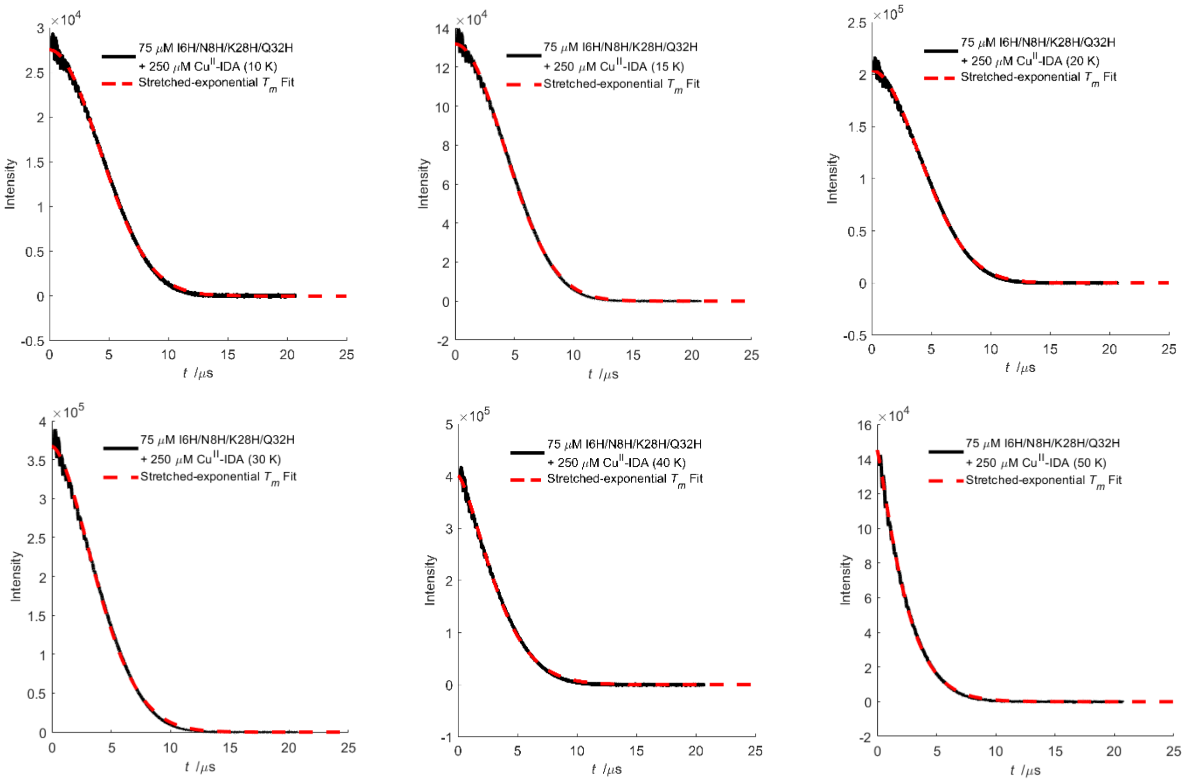

Figure S22. Two pulse electron spin echo traces for 75 μM I6H/N8H/K28H/Q32H GB1 + 250 μM Cu^{II}-IDA at various temperatures shown in black, with stretched exponential fits shown in red. The corresponding estimates for T_m are given in table S16 below.

Temperature [K]	T_m Estimate [μs]	Stretch Exponent
10	5.8	2.00
15	5.8	2.00
20	5.7	1.95
30	4.9	1.72
40	3.8	1.45
50	2.4	1.07

Table S16: Stretched exponential T_m estimates for the 75 μM I6H/N8H/K28H/Q32H GB1 + 250 μM Cu^{II}-IDA temperature series.

I6H/N8H/K28R1 GB1 (Cu^{II}-IDA):

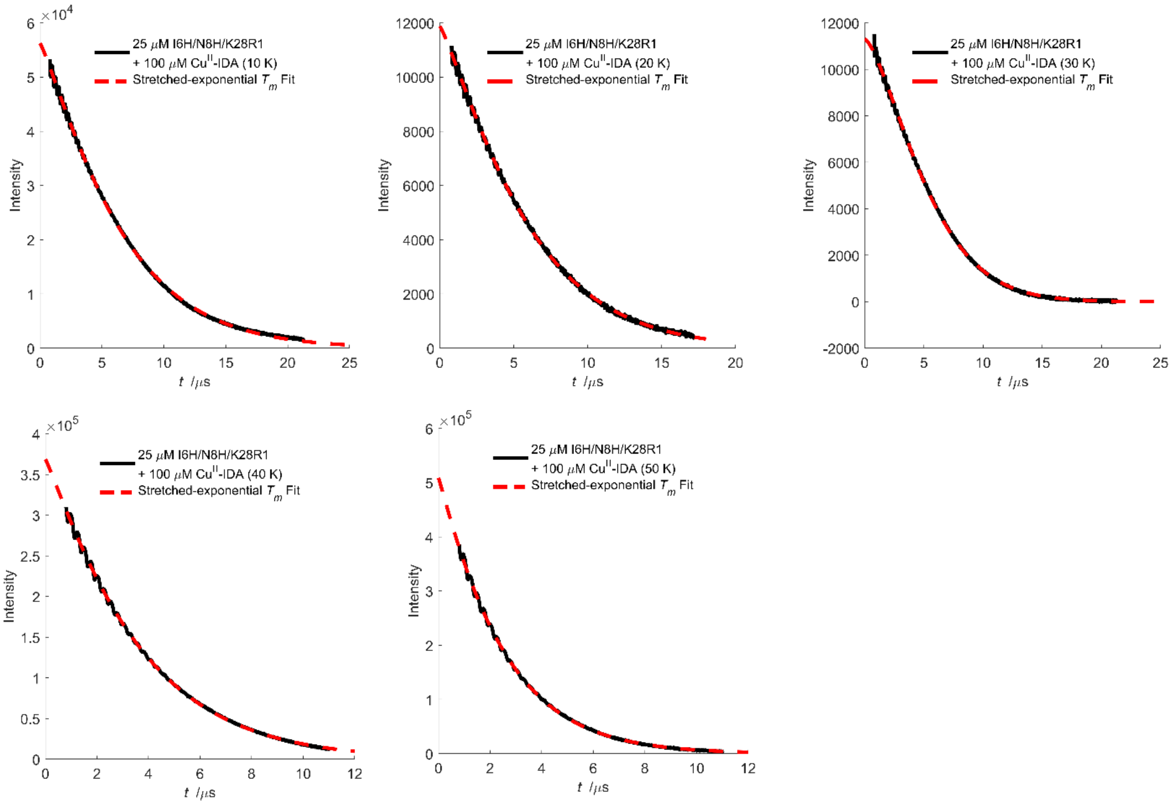

Figure S23. Two pulse electron spin echo traces of Cu^{II}-IDA for 25 μM I6H/N8H/K28R1 GB1 + 100 μM Cu^{II}-IDA at various temperatures shown in black, with stretched exponential fits shown in red. The corresponding estimates for T_m are given in table S17 below.

Temperature [K]	T_m Estimate [μs]	Stretch Exponent
10	6.8	1.17
20	6.2	1.19
30	5.9	1.45
40	3.7	1.10
50	2.6	1.07

Table S17: Stretched exponential T_m estimates for the 25 μM I6H/N8H/K28R1 GB1 + 100 μM Cu^{II}-IDA temperature series.

I6H/N8H/K28R1 GB1 (R1 nitroxide):

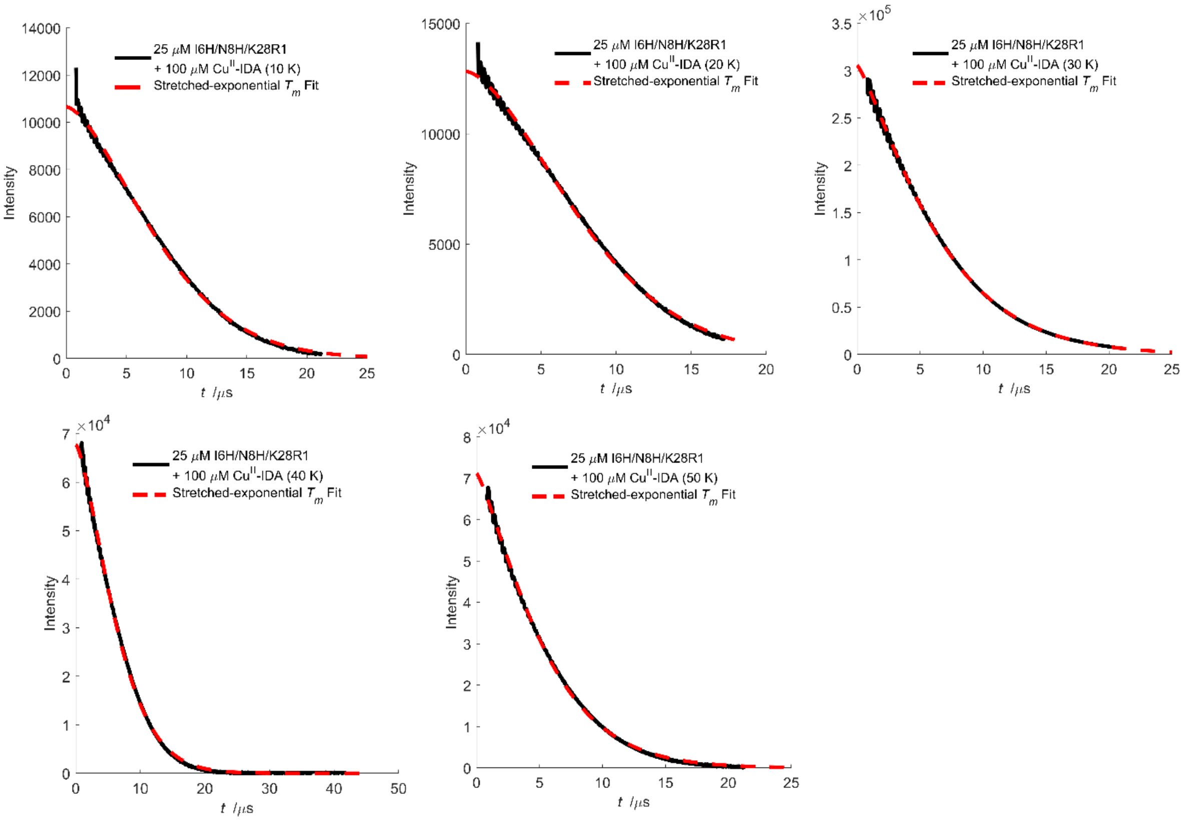

Figure S24. Two-pulse electron spin echo decay data of nitroxide for 25 μM I6H/N8H/K28R1 GB1 + 100 μM Cu^{II}-IDA at various temperatures shown in black, with stretched exponential fits shown in red. The corresponding estimates for T_m are given in table S18 below.

Temperature [K]	T_m Estimate [μs]	Stretch Exponent
10	9.1	1.58
20	9.2	1.63
30	7.0	1.23
40	7.3	1.45
50	5.8	1.26

Table S18: Stretched exponential T_m estimates of the nitroxide for the 25 μM I6H/N8H/K28R1 GB1 + 100 μM Cu^{II}-IDA temperature series.

2.9 Q-band RIDME:

The 5-pulse RIDME traces are shown for I6R1/K28H/Q32H and I6H/N8H/K28R1, in figures S25 and S26, respectively; the data is deconvoluted as described in section 1.10 and the modulation depth values (adjusted for deconvolution using equation 8) are given in table S19.

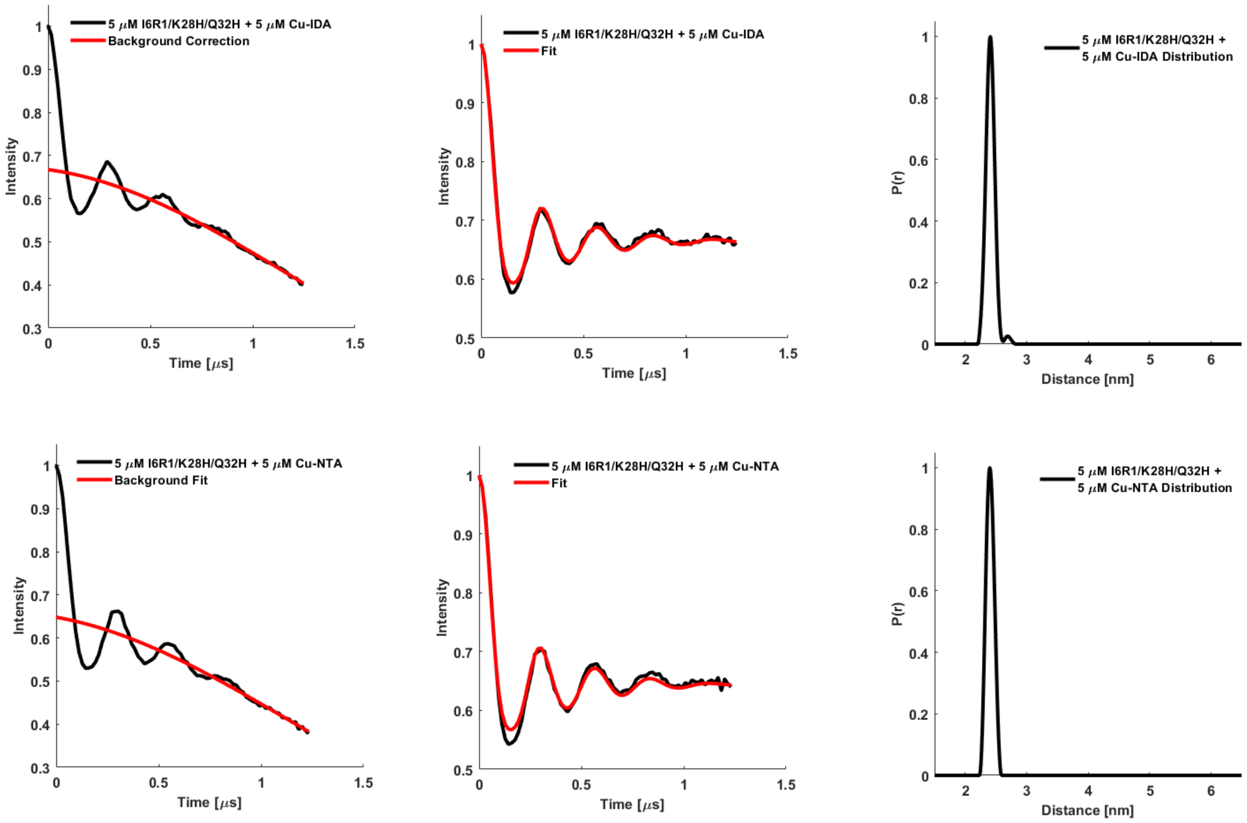

Figure S25. Deconvoluted RIDME data for 5 µM I6R1/K28H/Q32H, in presence of one equivalent Cu^{II}-IDA and Cu^{II}-NTA, shown in the top and bottom rows, respectively. The raw data, background corrected dipolar evolution function, and corresponding distance probability distributions *P*(*r*) are shown left-to-right. The experimental data is shown in black, with the respective background correction and fit to the dipolar evolution function shown in red.

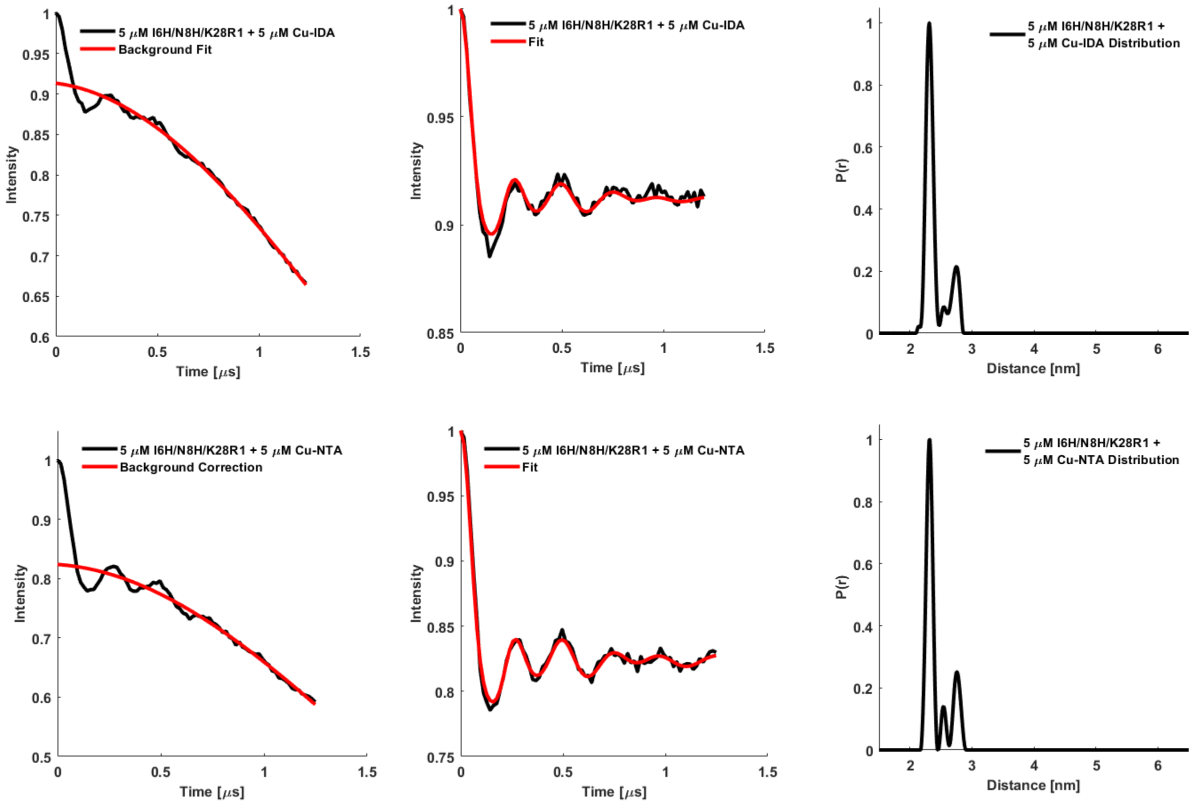

Figure S26. Deconvoluted RIDME data for 5 µM I6H/N8H/K28R1, in presence of one equivalent Cu^{II}-IDA and Cu^{II}-NTA, shown in the top and bottom rows, respectively. The raw data, dipolar evolution functions, and corresponding *P*(*r*) are shown left-to-right. The experimental data is shown in black, with the respective background correction and fit to the dipolar evolution function shown in red.

For the I6R1/K28H/Q32H construct, distance distributions are mono-modal at 2.40 nm and 2.41 nm for Cu^{II}-NTA and Cu^{II}-IDA, respectively. This is relatively consistent with the 2.45 nm distance reported between Cu^{II}-IDA labels in the tetra-histidine I6H/N8H/K28H/Q32H GB1 construct by Cunningham *et al.*,[1] and slight variation is to be anticipated given the R1-linker has a greater conformational freedom than the Cu^{II}-label.

Interestingly, the RIDME data for the I6H/N8H/K28R1 construct shows a bi-modal distance distribution; with a major peak at ~2.3 nm, and a smaller shoulder peak at ~2.7 nm, possibly indicating that the nitroxide is occupying distinct conformations on the helix. It is also feasible to suggest that the emergence of a shoulder peak indicates another Cu^{II}-label binding site, though at one equivalent of Cu^{II}-label this is unlikely to arise from non-specific interactions. To clarify that a second Cu^{II}-binding site was not present, a Cu^{II}-Cu^{II} RIDME measurement was performed, and the raw data is shown in figure S34 indicating no dipolar modulation. A further indication that this shoulder peak at 2.7 nm is a real feature, and not the result of improper background correction is that the dipolar evolution functions in figure S26 both distinctly show modulation by a second dipolar frequency. To further investigate this, X-band PELDOR traces were recorded for both constructs in presence of 1.5 equivalents of each Cu^{II}-chelate; shown in figures S35 and S36 and further discussed in section 2.10.

Importantly, the dipolar evolution functions in figure S25 and S26 indicate high affinity for both constructs in combination with both Cu^{II}-labels. These measurements directly contradict the high K_D values previously estimated for Cu^{II}-IDA[1] and Cu^{II}-NTA.[15] This could be explained by the significantly increased concentrations (approximately six-fold or more) of both protein and Cu^{II}-labels used in previous literature, relative to this work and by the different methods used for quantifying bound *versus* unbound Cu^{II} ions.

Sample	Modulation depth (Δ)
5 µM I6R1/K28H/Q32H + 5 µM Cu^{II}-IDA	0.35±0.01
5 µM I6R1/K28H/Q32H + 5 µM Cu^{II}-NTA	0.37±0.01
5 µM I6H/N8H/K28R1 + 5 µM Cu^{II}-IDA	0.09±0.01
5 µM I6H/N8H/K28R1 + 5 µM Cu^{II}-NTA	0.18±0.01

Table S19: The modulation depths of the deconvoluted RIDME traces shown above in figures S25 and S26, as adjusted according to equation 8, given above.

The 5-pulse RIDME traces for the 0.5 µM I6R1/K28H/Q32H Cu^{II}-NTA pseudo-titration series in the main text, are shown in figures S27-31, for 0.1, 0.3, 0.9, 2.7 and 8.1 µM Cu^{II}-NTA respectively. This data is not deconvoluted. This is empirically found to lead to a pronounced first minimum of the oscillation that is not reproduced by the fitting. This artefact is reduced by deconvolution. The associated modulation depths are given in table S20.

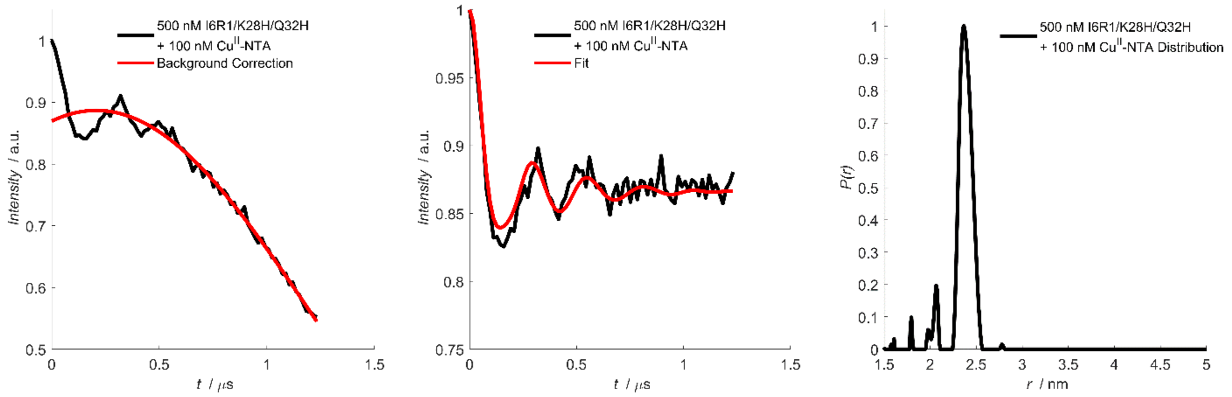

Figure S27. Non-deconvoluted RIDME data for 0.5 µM I6R1/K28H/Q32H, in presence of 0.1 µM Cu^{II}-NTA. The raw data, dipolar evolution function, and corresponding distance distribution are shown left-to-right. The experimental data is shown in black, with the respective background correction and fit to the dipolar evolution function shown in red.

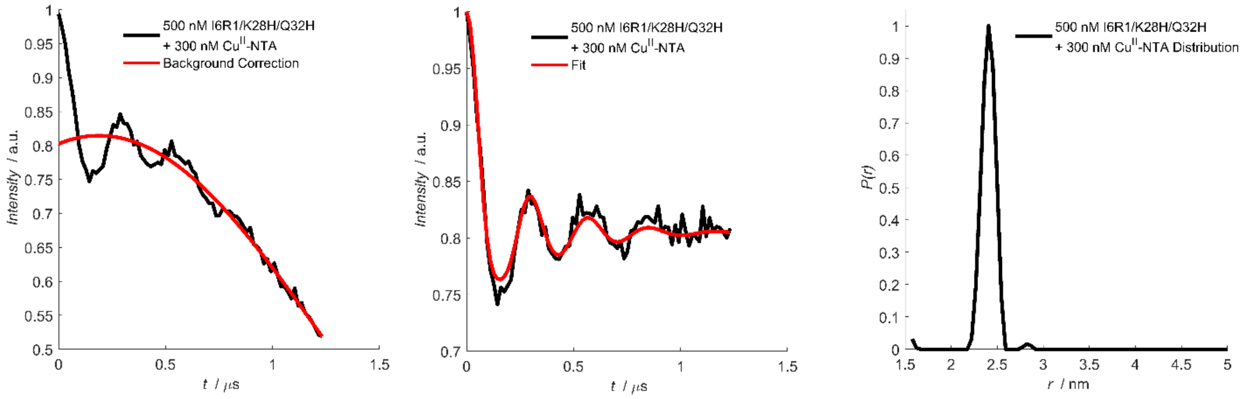

Figure S28. Non-deconvoluted RIDME data for 0.5 µM I6R1/K28H/Q32H, in presence of 0.3 µM Cu^{II}-NTA. The raw data, dipolar evolution function, and corresponding distance distribution are shown left-to-right. The experimental data is shown in black, with the respective background correction and fit to the dipolar evolution function shown in red.

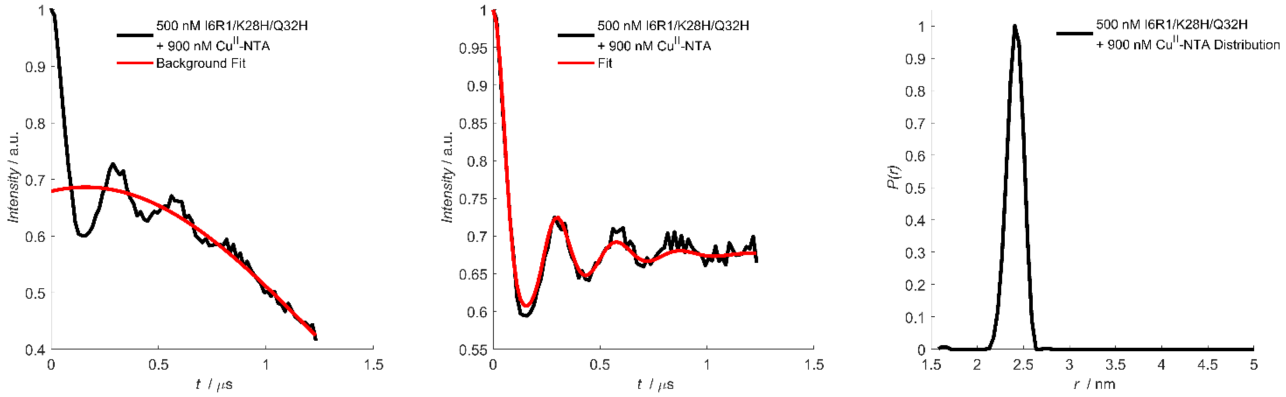

Figure S29. Non-deconvoluted RIDME data for 0.5 µM I6R1/K28H/Q32H, in presence of 0.9 µM Cu^{II}-NTA. The raw data, dipolar evolution function, and corresponding distance distribution are shown left-to-right. The experimental data is shown in black, with the respective background correction and fit to the dipolar evolution function shown in red.

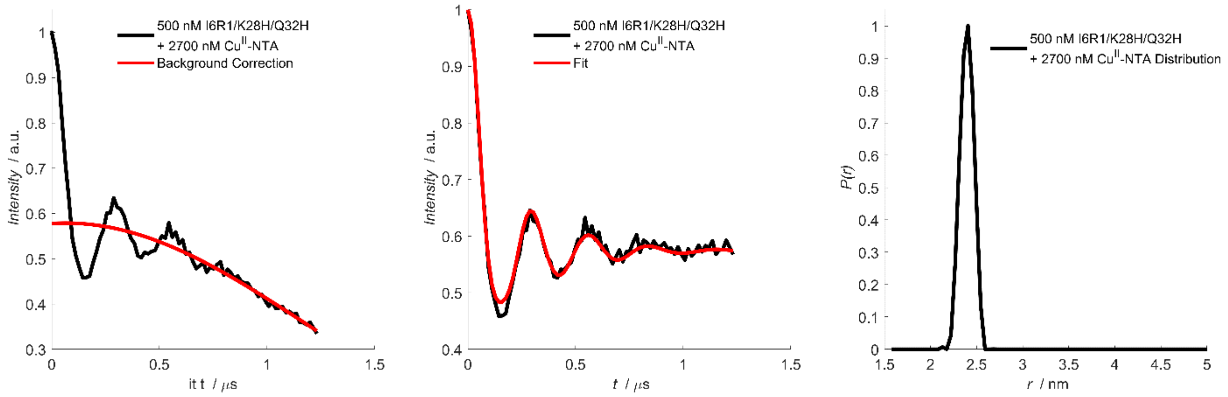

Figure S30. Non-deconvoluted RIDME data for 0.5 µM I6R1/K28H/Q32H, in presence of 2.7 µM Cu^{II}-NTA. The raw data, dipolar evolution function, and corresponding distance distribution are shown left-to-right. The experimental data is shown in black, with the respective background correction and fit to the dipolar evolution function shown in red.

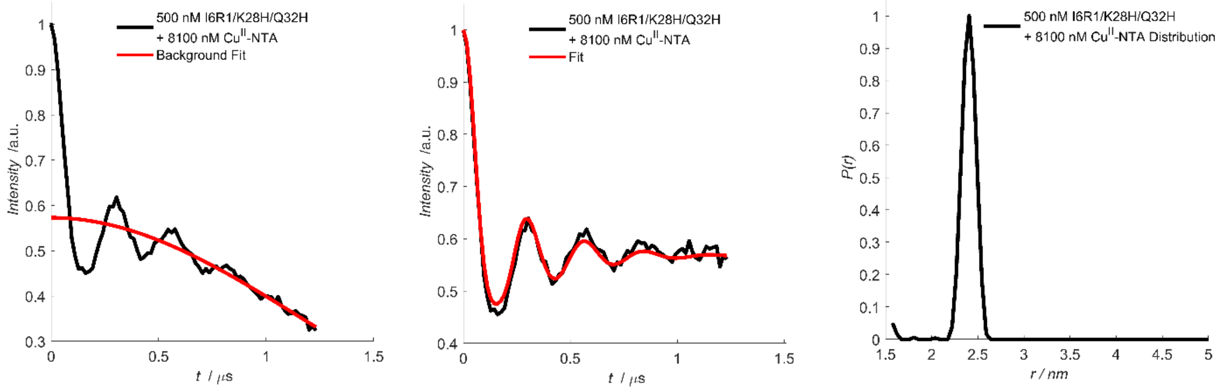

Figure S31. Non-deconvoluted RIDME data for 0.5 µM I6R1/K28H/Q32H, in presence of 8.1 µM Cu^{II}-NTA. The raw data, dipolar evolution function, and corresponding distance distribution are shown left-to-right. The experimental data is shown in black, with the respective background correction and fit to the dipolar evolution function shown in red.

Sample	Modulation depth (Δ)
I6R1/K28H/Q32H + 0.1 µM Cu^{II}-NTA	0.13
I6R1/K28H/Q32H + 0.3 µM Cu^{II}-NTA	0.20
I6R1/K28H/Q32H + 0.9 µM Cu^{II}-NTA	0.32
I6R1/K28H/Q32H + 2.7 µM Cu^{II}-NTA	0.42
I6R1/K28H/Q32H + 8.1 µM Cu^{II}-NTA	0.43

Table S20. The modulation depths of the non-deconvoluted RIDME traces shown in figures S27-31, given above.

Furthermore, the non-deconvoluted modulation depths for the pseudo-titration series shown in section 2.13, are given in tables S21-24 for 25 µM and 75 µM I6R1/K28H/Q32H in presence of Cu^{II}-NTA and Cu^{II}-IDA, respectively, and 25 µM I6H/N8H/K28R1 in presence of Cu^{II}-NTA and Cu^{II}-IDA. The raw and processed RIDME traces will be published with the underpinning data.

Sample	Modulation depth (Δ)
25 µM I6R1/K28H/Q32H + 15 µM Cu^{II}-NTA	0.26
25 µM I6R1/K28H/Q32H + 30 µM Cu^{II}-NTA	0.39
25 µM I6R1/K28H/Q32H + 60 µM Cu^{II}-NTA	0.39
25 µM I6R1/K28H/Q32H + 135 µM Cu^{II}-NTA	0.39
25 µM I6R1/K28H/Q32H + 960 µM Cu^{II}-NTA	0.40

Table S21. The modulation depths of the non-deconvoluted 25 µM I6R1/K28H/Q32H RIDME pseudo-titration in presence of Cu^{II}-NTA.

Sample	Modulation depth (Δ)
75 µM I6R1/K28H/Q32H + 45 µM Cu^{II}-IDA	0.27
75 µM I6R1/K28H/Q32H + 100 µM Cu^{II}-IDA	0.40
75 µM I6R1/K28H/Q32H + 185 µM Cu^{II}-IDA	0.42
75 µM I6R1/K28H/Q32H + 350 µM Cu^{II}-IDA	0.43
75 µM I6R1/K28H/Q32H + 600 µM Cu^{II}-IDA	0.43
75 µM I6R1/K28H/Q32H + 1750 µM Cu^{II}-IDA	0.46

Table S22. The modulation depths of the non-deconvoluted 75 µM I6R1/K28H/Q32H RIDME pseudo-titration in presence of Cu^{II}-IDA.

Sample	Modulation depth (Δ)
25 µM I6H/N8H/K28R1+ 10 µM Cu^{II}-NTA	0.15
25 µM I6H/N8H/K28R1+ 20 µM Cu^{II}-NTA	0.31
25 µM I6H/N8H/K28R1+ 40 µM Cu^{II}-NTA	0.39
25 µM I6H/N8H/K28R1+ 90 µM Cu^{II}-NTA	0.42
25 µM I6H/N8H/K28R1+ 600 µM Cu^{II}-NTA	0.43

Table S23. The modulation depths of the non-deconvoluted 25 µM I6H/N8H/K28R1 RIDME pseudo-titration in presence of Cu^{II}-NTA.

Sample	Modulation depth (Δ)
25 µM I6H/N8H/K28R1+ 10 µM Cu^{II}-IDA	0.29
25 µM I6H/N8H/K28R1+ 20 µM Cu^{II}-IDA	0.30
25 µM I6H/N8H/K28R1+ 35 µM Cu^{II}-IDA	0.38
25 µM I6H/N8H/K28R1+ 75 µM Cu^{II}-IDA	0.42
25 µM I6H/N8H/K28R1+ 450 µM Cu^{II}-IDA	0.45

Table S24. The modulation depths of the non-deconvoluted 25 µM I6H/N8H/K28R1 RIDME pseudo-titration in presence of Cu^{II}-IDA.

The 5-pulse RIDME traces for the buffer and cryoprotectant controls are shown below for 50 mM phosphate or N-ethyl morpholine (NEM) buffer conditions,[1] and 50% (v/v) *d-6* ethylene glycol or 20% (v/v) *d-8* glycerol[1] as cryo-protectants, in figures S32 and S33 respectively. The data is deconvoluted and the associated modulation depths are given in table S25. There is ~15% variation in the Δ between the four permutations, and an apparent decreasing trend as conditions change from 50% ethylene glycol cryoprotectant and 50 mM phosphate buffer to 20% glycerol cryoprotectant and 50 mM NEM buffer. This is speculated to occur for two reasons; the first is that a reduction in cryoprotectant will correspondingly reduce the formation of an adequate glass, and therefore contribute to regions of high local concentrations within the sample. The second reason is associated with the chosen buffer conditions; NEM can compete with the double histidine motif to co-ordinate Cu^{II}-IDA. In any case, a 15% variation in the values of Δ does not account for the apparent 1000- fold increase in affinity, and therefore disparities between buffer and cryoprotectant conditions used in this work and previous literature are not the source of this observation.

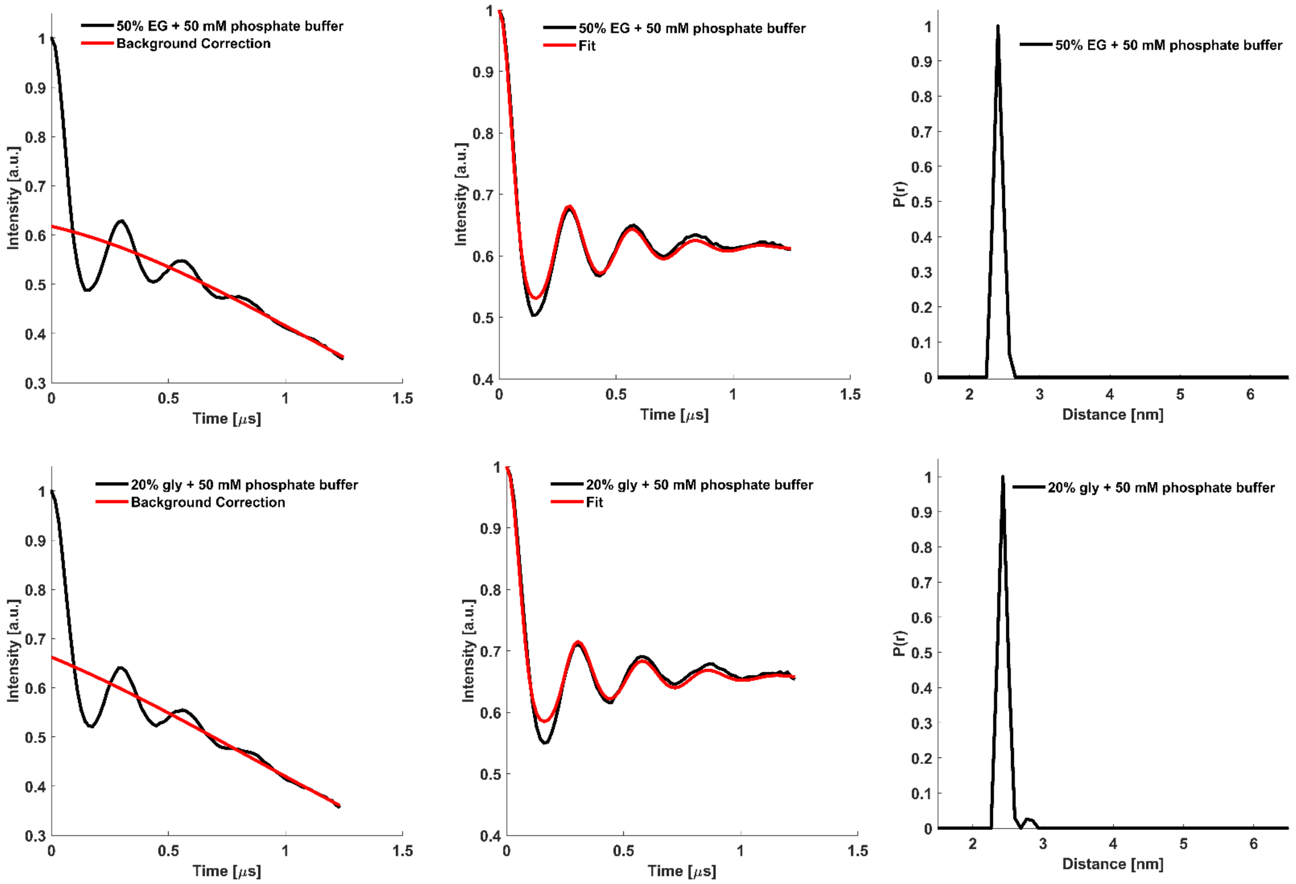

Figure S32. Deconvoluted RIDME data for 75 µM I6R1/K28H/Q32H, in presence of 75 µM Cu^{II}-IDA and 50 mM sodium phosphate buffer condition. The different cryoprotectant conditions, 50% ethylene glycol and 20% glycerol are shown in the top and bottom row respectively. The raw data, dipolar evolution function, and corresponding distance distribution are shown left-to-right. The experimental data is shown in black, with the respective background correction and fit to the dipolar evolution function shown in red.

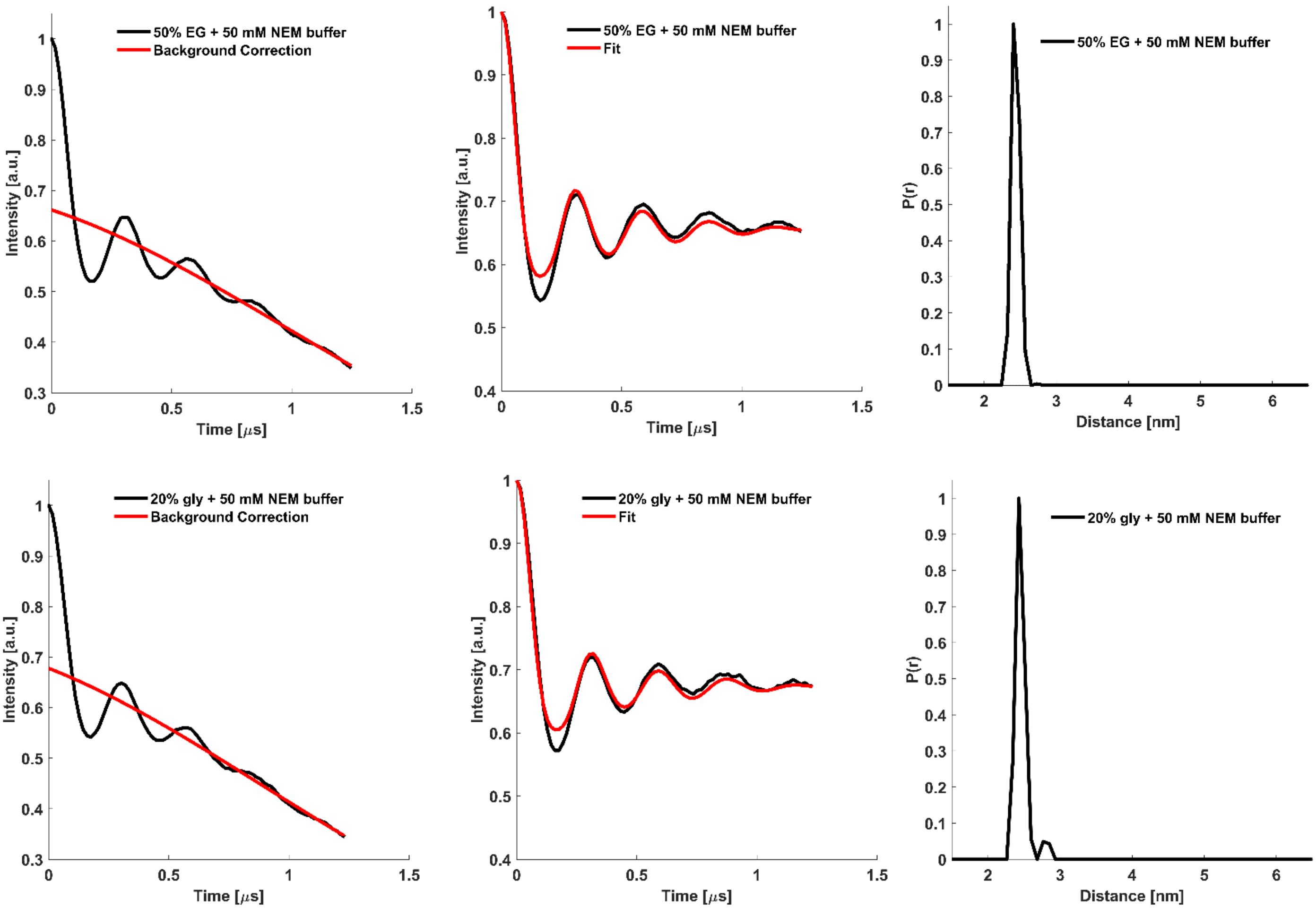

Figure S33. Deconvoluted RIDME data for 75 μM I6R1/K28H/Q32H, in presence of 75 μM Cu^{II}-IDA and 50 mM N-ethyl morpholine buffer condition. The different cryoprotectant conditions, 50% ethylene glycol and 20% glycerol are shown in the top and bottom row respectively. The raw data, dipolar evolution function, and corresponding distance distribution are shown left-to-right. The experimental data is shown in black, with the respective background correction and fit to the dipolar evolution function shown in red.

Sample	Modulation depth (Δ)
50 mM phosphate buffer + 50% ethylene glycol	0.38±0.01
50 mM phosphate buffer + 20% glycerol	0.34±0.01
50 mM N-ethyl morpholine + 50% ethylene glycol	0.34±0.01
50 mM N-ethyl morpholine + 20% glycerol	0.32±0.01

Table S25. The modulation depths of the deconvoluted RIDME traces shown in figures S32-33, given above.

Since the I6H/N8H/K28R1 construct gave a bi-modal distance distribution when measured with 5-pulse RIDME, it was necessary to determine that there was only one Cu^{II}-binding site, as expected; this was most relevant because the model used to fit the experimental data and estimate the K_D values assumed a single binding-site. Therefore, a sample of I6H/N8H/K28R1 in presence of Cu^{II}-NTA was measured by 5-pulse RIDME, with the detection pulses positioned at the maximum of the Cu^{II}-NTA spectrum and using a mixing block of ~0.7×T_1. The raw trace is shown in figure S34, and as can be seen there is no dipolar modulation, indicating that there are no secondary Cu^{II}-NTA binding sites.

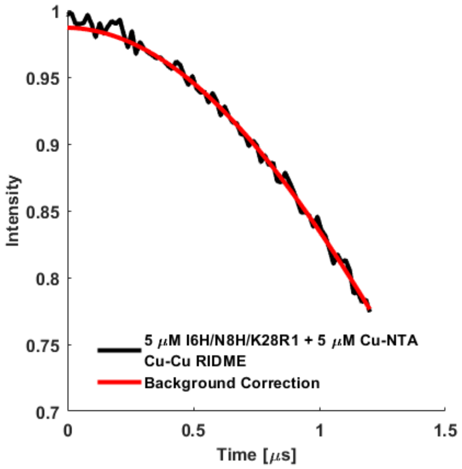

Figure S34. A Cu^{II}-Cu^{II} RIDME trace, measured on the I6H/N8H/K28R1 + Cu^{II}-NTA sample. Data was acquired overnight (~16 hours), using an SRT of 2 ms and a mixing-time of 30 µs, observing at the maximum field position of the Cu^{II}-NTA spectrum. The parameters of background correction are given in table S26.

Sample	Zero-time [ns]	Background Start [ns]	Background Cut-off [ns]
Cu^{II}-Cu^{II} RIDME I6H/N8H/K28R1 + Cu^{II}-NTA	249	236	1200

Table S26. Parameters of the background correction of the Cu^{II}-Cu^{II} RIDME data shown in figure S34.

2.10 X-band PELDOR:

For the I6R1/K28H/Q32H construct, 5-pulse RIDME measurements gave a mono-modal distance distribution that agreed reasonably with that expected from the molecular dynamics simulation; the MMM simulation of I6R1/K28H/Q32H also reproduced the experimental distributions, albeit slightly shifted to a longer modal distance. However, for the I6H/N8H/K28R1 construct, there was a shift of the major distance peak, from 2.40 to 2.33 nm, and the distribution had a second minor peak in the region 2.7-2.9 nm. The MMM simulation produces a bimodal distribution shifted to significantly shorter distances than empirically observed. Therefore, the purpose of measuring at X-band frequency using the 4-pulse PELDOR experiment was to affirm the validity of the distance distribution measured *via* 5-pulse RIDME for the I6H/N8H/K28R1 construct.

Results of the 4-pulse PELDOR measurements are shown below for I6R1/K28H/Q32H and I6H/N8H/K28R1 in figures S35 and S36, and in presence of Cu^{II}-IDA and Cu^{II}-NTA, shown in blue and red respectively. The 5-pulse Q-band RIDME measurements for Cu^{II}-IDA and Cu^{II}-NTA, molecular dynamics simulated distributions, and the corresponding distributions simulated in MMM are also overlaid and compared for each construct, shown in magenta, cyan, black and green respectively. The X-band PELDOR measurements reproduce the distance distributions seen in the RIDME experiment for all construct-chelate permutations. It is interesting to note that for the I6H/N8H/K28R1 construct the molecular dynamics simulation in XPLOR significantly outperforms MMM and agrees well with all empirical data. However, for the I6R1/K28H/Q32H construct, the molecular dynamics simulation shifts the modal distance ~0.3 nm higher than observed and fails to reproduce the exact distribution-shape, while MMM predicts both the modal distance and distribution-width more accurately. Note that these initial samples had issues with the determination of the absolute protein concentration leading to significant errors in the absolute modulation depths. Nonetheless, these measurements allow relating the distance distributions obtained by RIDME to results from the more established PELDOR technique.

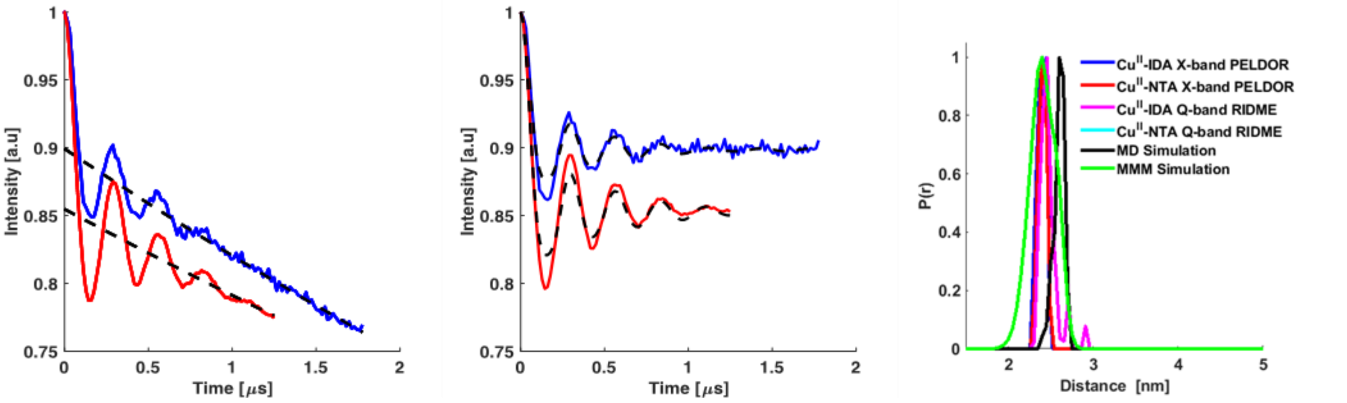

Figure S35. X-band PELDOR raw and processed data for I6R1/K28H/Q32H GB1 in presence of Cu^{II}-IDA and Cu^{II}-NTA shown in blue and red respectively, with the background correction, and corresponding fits in black. In the right-most panel, distance distributions are overlaid with those obtained for Q-band RIDME, in presence of Cu^{II}-IDA and Cu^{II}-NTA, and with the distribution predicted by molecular dynamics, and MMM2018 shown in magenta, cyan, black and green respectively.

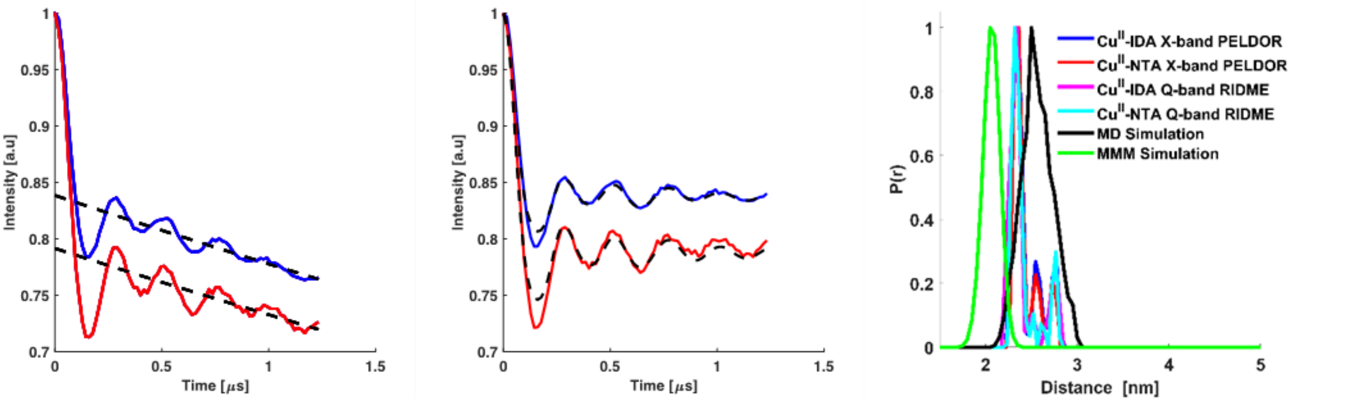

Figure S36. X-band PELDOR raw and processed data for I6H/N8H/K28R1 GB1 in presence of Cu^{II}-IDA and Cu^{II}-NTA shown in blue and red respectively, the corresponding background correction and fit are shown in dotted black. In the right-most panel, distance distributions are overlaid with those obtained for Q-band RIDME, in presence of Cu^{II}-IDA and Cu^{II}-NTA, and with the distribution predicted by molecular dynamics, and MMM2018, shown in magenta, cyan, black and green respectively.

2.11 Sensitivity Optimisation of Cu^{II}-Cu^{II} RIDME and PELDOR Measurements:

A sensitivity profile for Cu^{II}-Cu^{II} RIDME was numerically simulated using T_1 and T_m estimates from 75 µM I6H/N8H/K28H/Q32H GB1 + 250 µM Cu^{II}-IDA, according to equations 4 and 5 given in section 1.9. The contour plots are shown in figure S37 below.

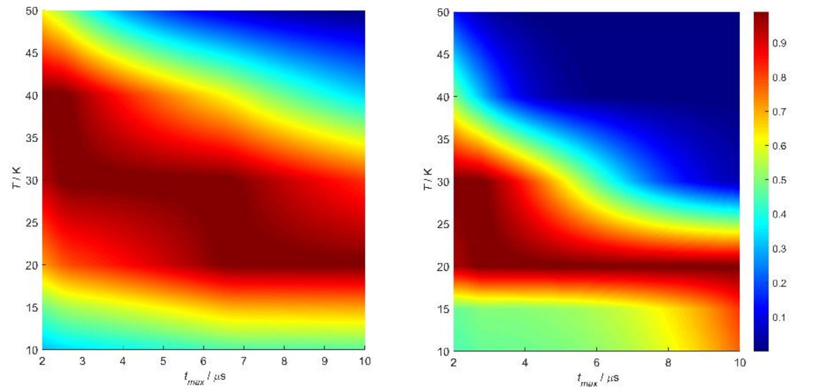

Figure S37. Sensitivity contour plots of the 5-pulse Cu^{II}-Cu^{II} RIDME experiment, for the 75 µM I6H/N8H/K28H/Q32H + 250 µM Cu^{II}-IDA sample, numerically simulated from equations 4 (left) and 5 (right) to investigate the influence of using a bi-exponential and stretched exponential approximation to describe the longitudinal and transverse relaxation times, respectively.

The general sensitivity profile is conserved when using a bi-exponential approximation to describe longitudinal relaxation, however the transition from 30 K to 20 K shifts to shorter t_{max} values, though for our measurements 30 K is still the optimal temperature. It is observed from the contour plots in figure S38 that regardless of the model used for simulation, sensitivity is maximised for Cu^{II}-Cu^{II} RIDME by measuring at 30 K for $t_{max} < 4$ µs.

It was also necessary to optimise parameters for the 4-pulse Cu^{II}-Cu^{II} PELDOR measurements. Here, the sensitivity contour was numerically simulated according to equation 7, in the temperature range from 10 to 50 K and for t_{max} in the range from 2 to 10 µs, and is shown in figure S38. It is important to note that the normalised contour plots should appear numerically identical to that of the Cu^{II}-Cu^{II} RIDME contour; this is because for a homo-spin pair the additional exponential terms in the RIDME expression are constant, under the assumption that $T_{mix} \sim 0.7 \times T_1$.

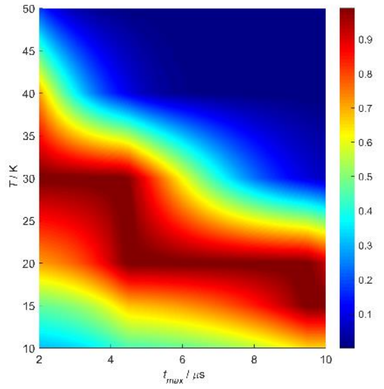

Figure S38. Sensitivity contour plots of the 4-pulse Cu^{II}-Cu^{II} PELDOR experiment, using experimental values for the 75 µM I6H/N8H/K28H/Q32H + 250 µM Cu^{II}-IDA sample, numerically simulated from equation 7.

Data suggest that using a mono-exponential approximation to estimate T_1 and T_m relaxation times leads to an over-estimation of the relative sensitivity at higher temperatures as seen in figure S38. The use of a bi-exponential approximation to estimate T_1 leads to a reduction in the optimum temperature, as shown in the right panel of figure S37. It is to be expected that at longer t_{max} the optimum temperature reduces, and in this case for $t_{max} > 4$ µs measuring at 20 K is likely to be optimal. In this vein, the bi-exponential approximation and stretched exponential approximation, for estimation of T_1 and T_m respectively, are used to determine sensitivity contours, using equation 5.

For the Cu^{II}-nitroxide RIDME, sensitivity contour plots simulated using equations 4 and 5, for temperatures from 10 to 50 K and in the t_{max} range from 2 to 10 µs are shown in figure S39 below. It is seen that under the mono-exponential approximation there is a normalised sensitivity of > 0.90 at 30 K for $t_{max} \leq 4$ µs, while under the bi-exponential approximation the sensitivity profile is noticeably changed and a splitting of the sensitivity maximum is observed at both 20 and 40 K. It is important to note that this is likely artificial owing to imperfect fits of experimental ESE decay and IR data at low temperature, and particularly the behaviour of the stretching exponent in the transverse dephasing term, which does not decrease significantly with lower temperatures, as expected. Under the bi-exponential approximation normalised sensitivity at 30 K is ≥ 0.55 at $t_{max} \leq 4$ µs.

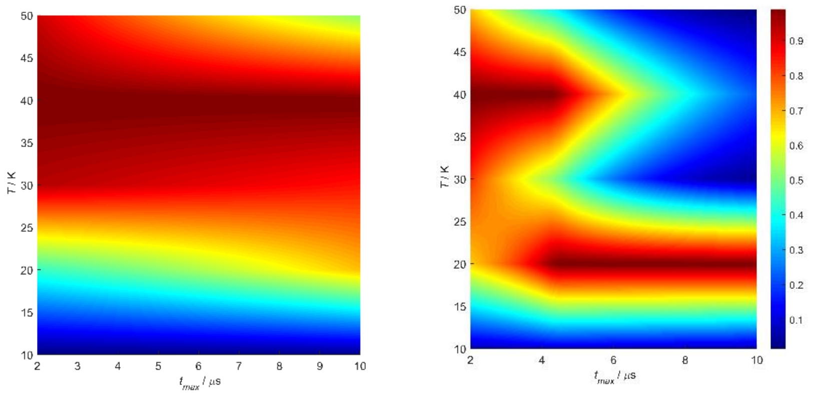

Figure S39. Contour plot of sensitivity calculated from equations 4 (left) and 5 (right) in the 5-pulse Cu^{II}-NO^{II} RIDME experiment, using experimental values for the 25 µM I6H/N8H/K28R1 + 100 µM Cu^{II}-IDA sample, for temperatures in the range 10-50 K, numerically simulating t_{max} in the range 2-10 µs.

To optimise the experimental T_{mix} used for Cu^{II}-nitroxide RIDME measurements, sensitivity contour plots were numerically simulated from equations 4 and 5 by varying the mixing block interval (T_{mix}) in the range 0-1000 µs for t_{max} = 1.5 µs and 4 µs, shown in figures S40 and S41 respectively. It is seen that for the mono-exponential approximation (figure S40), normalised sensitivity ≥ 0.95 is achieved at 30 K with a mixing time of ~100-200 µs, while for the bi-exponential approximation (figure S41) it is seen that for t_{max} = 1.5 µs a normalised sensitivity ≥ 0.90 is achieved at 30 K with a mixing time of 200 µs. At t_{max} = 4 µs normalised sensitivity at 30 K falls to ~0.55, but is still optimised at that temperature with a mixing time of 200 µs.

This process optimises sensitivity, while also minimising the effect of spectral diffusion by keeping the mixing time short. This is significant because spectral diffusion is more prominent at longer mixing times and can lead to RIDME background functions which are fitted poorly by second-order polynomials, making background correction problematic. However, it should also be mentioned that for the quantification of Δ and specifically the product $\Delta \times \Delta_{Tmix}{}^{-1}$, a longer mixing time can be beneficial, particularly when $T_{mix} >> T_1$, since in this case the product $\Delta \times \Delta_{Tmix}{}^{-1}$ is less sensitive to error in T_1 estimation, and the robustness of the approach is increased. Therefore, a mixing time of 200 µs was used for all Cu^{II}-nitroxide 5-pulse RIDME measurements.

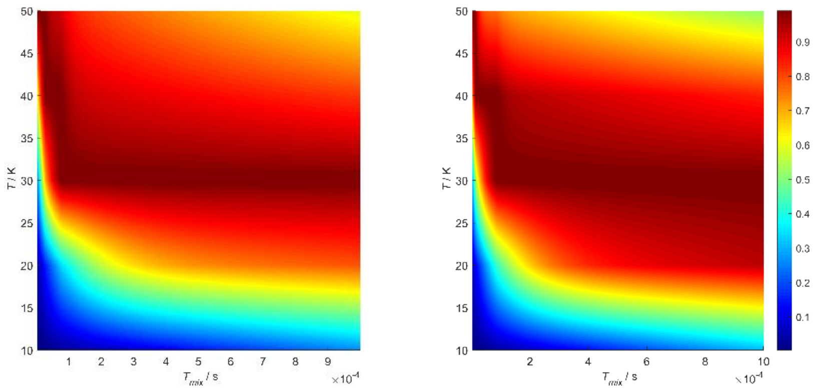

Figure S40. Contour plot of sensitivity calculated from equation 4 in the 5-pulse Cu^{II}-NO^{II} RIDME experiment, using experimental values for the 25 µM I6H/N8H/K28R1 + 100 µM Cu^{II}-IDA sample, for temperatures in the range 10-50 K, numerically simulating mixing block intervals (T_{mix}) in the range 0-1000 µs, and for t_{max} = 1.5 µs (left) and 4 µs (right).

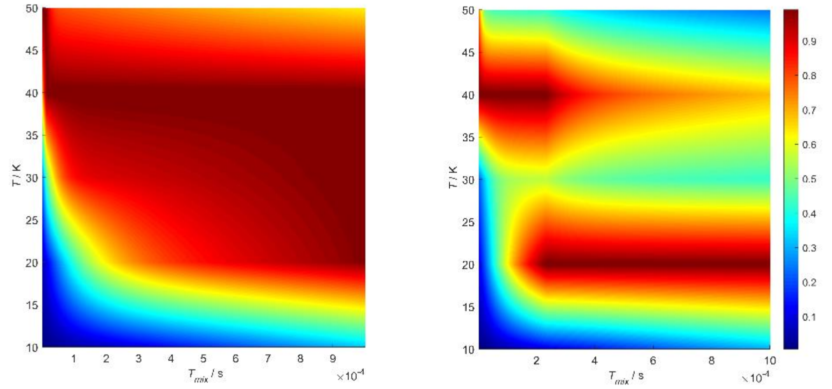

Figure S41. Contour plot of sensitivity calculated from equation 5 in the 5-pulse Cu^{II}-NO^{II} RIDME experiment, for the 25 µM I6H/N8H/K28R1 + 100 µM Cu^{II}-IDA sample, for temperatures in the range 10-50 K, numerically simulating mixing block intervals (T_{mix}) in the range 0-1000 µs, and for t_{max} = 1.5 µs (left) and 4 µs (right).

2.12 RIDME Sensitivity Estimation:

The sensitivity improvement when comparing Q-band Cu^{II}-NO RIDME and Cu^{II}-Cu^{II} RIDME and PELDOR was estimated using an approach outlined previously.[16] In this approach, the modulation depth of each trace is calculated in DeerAnalysis, then the noise level in each trace is estimated from the imaginary part of the phase-corrected and normalised time-trace, and the modulation depth is divided by this value, giving the sensitivity as modulation-to-noise ratio. This is then normalised for number of echoes by division with the square-root of the total echoes per point considering all averaging and phase cycles giving the sensitivity per echo (S_e). Different signal averaging rates based on different relaxation times can be taken into account by multiplying with the square root of the averaging rate to yield the sensitivity per unit time (S_t).

For a comparison of the sensitivity of Cu^{II}-Cu^{II} and Cu^{II}-nitroxide RIDME and Cu^{II}-Cu^{II} PELDOR, samples of 25 µM I6R1/K28H/Q32H GB1 + 30 µM Cu^{II}-NTA and 25 µM I6H/N8H/K28H/Q32H GB1 + 50 µM Cu^{II}-NTA were prepared (to give approximately equimolar equivalents of double-histidine motifs and Cu^{II}-NTA). Samples were measured at 30 K and 122 points, recorded with respective shot-repetition times of 1, 2, and 30 ms for Cu^{II}-Cu^{II} RIDME, PELDOR and Cu^{II}-nitroxide RIDME measurements (corresponding to rates of 1,000, 500 and 33 Hz). For the Cu^{II}-Cu^{II} and Cu^{II}-nitroxide RIDME respectively, mixing block intervals (T_{mix}) of 34 and 200 µs were used.

At this concentration, the Cu^{II}-nitroxide RIDME trace shows strong dipolar modulation even after a single scan and single shot-per-point, this was problematic for estimation of the trace noise, since the root-mean square deviation (RMSD) is dominated by the error in the Tikhonov fitting rather than the noise. Imperfections of the phase cycle and other artefacts dominated the imaginary part. Therefore, dummy RIDME and PELDOR traces were run, at which the respective echo was integrated multiple times at $t = 0$ and dipolar evolution was not incremented. This has made nuclear modulation averaging obsolete and it was not used for dummy experiments. However, for capturing the experimental phase cycling schemes, dummy RIDME traces were recorded with one shot per point and an 8-step phase cycle (i.e., each point corresponds to 8 total echoes). To compensate for this, the dummy PELDOR trace was recorded using four shots per point and a 2-step phase cycle (i.e., each point corresponds to 8 total echoes). The resulting noise traces are shown below in figure S42, and the calculated RMSD is given in table S27. These results show that the detected echo carries the lowest noise for Cu^{II}-nitroxide RIDME whereas it is approximately a factor 3 and 15 higher for Cu^{II}-Cu^{II} RIDME and PELDOR, respectively. This does not yet consider modulation depths or signal averaging.

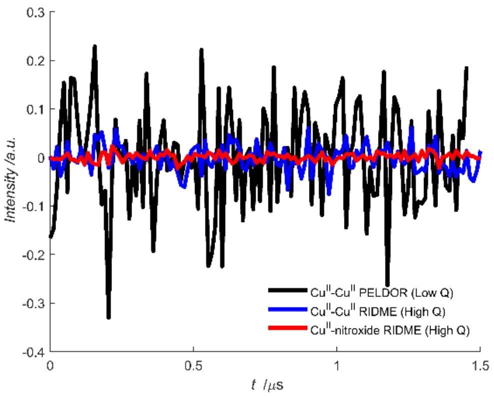

Figure S42: A comparison of the noise traces measured for Cu^{II}-Cu^{II} PELDOR and Cu^{II}-Cu^{II} and Cu^{II}-nitroxide RIDME experiments, shown respectively in black, blue and red.

Sample	Experiment	RMSD Estimate	Relative noise
25 µM I6H/N8H/K28H/Q32H GB1 + 50 µM Cu^{II}-NTA	Cu^{II}-Cu^{II} PELDOR	1.38 x 10^{-1}	14.9
25 µM I6H/N8H/K28H/Q32H GB1 + 50 µM Cu^{II}-NTA	Cu^{II}-Cu^{II} RIDME	2.93 x 10^{-2}	3.16
25 µM I6R1/K28H/Q32H GB1 + 30 µM Cu^{II}-NTA	Cu^{II}-nitroxide RIDME	9.26 x 10^{-3}	1.00

Table S27: A comparison of the estimated RMSD of the noise traces shown in figure S42. Dummy measurements were run by setting the period of dipolar evolution to 0 and not incrementing *t*, therefore yielding traces without dipolar modulation.

In this work, Cu^{II}-Cu^{II} PELDOR modulation depths at Q-band were found to be limited to ~1%, while Cu^{II}-Cu^{II} RIDME is experimentally observed to yield 20% and Cu^{II}-nitroxide RIDME is (in practice) limited to ~45%. Experimental raw and processed RIDME and PELDOR traces are shown below in figures S43-45, with modulation depths and background correction parameters given in table S28. As only the modulated part of the signal contains the desired structural information, the modulation depths contributes linearly to the effect sensitivity. This can be expressed in the modulation-to-noise ratio (given by the modulation depth divided by the root mean square noise). For the present comparison this yields the highest sensitivity per echo (S_e) for Cu^{II}-nitroxide RIDME whereas it is factors ~7 and ~670 lower for Cu^{II}-Cu^{II} RIDME and PELDOR, respectively. This does not yet consider signal averaging.

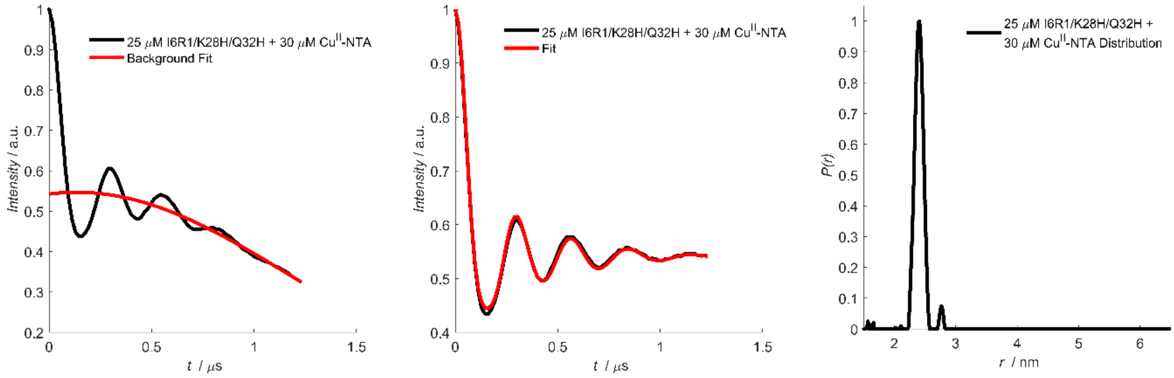

Figure S43: Cu^{II}-nitroxide RIDME measured for 25 µM I6R1/K28H/Q32H GB1 + 30 µM Cu^{II}-NTA, with the raw trace, background corrected trace and distance distribution shown left-to-right. Data is shown in black, and the background correction and Tikhonov fit are shown in red.

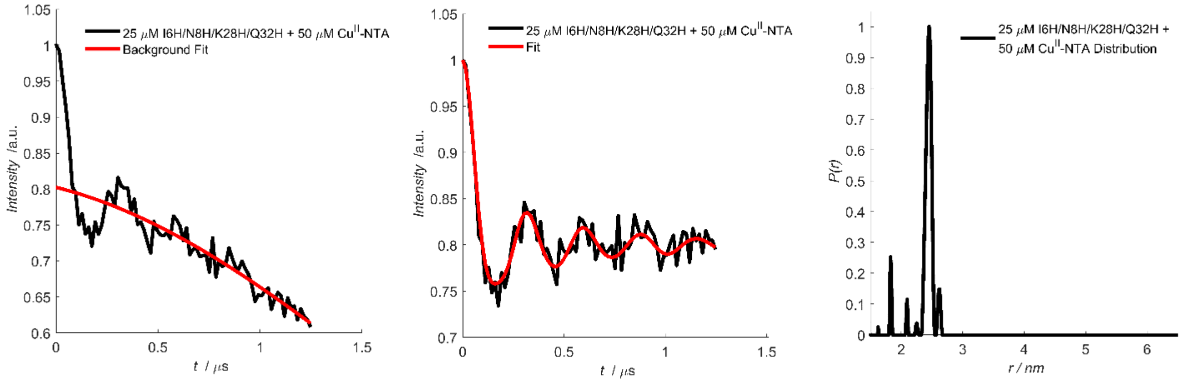

Figure S44: Cu^{II}-Cu^{II} RIDME measured for 25 µM I6H/N8H/K28H/Q32H GB1 + 50 µM Cu^{II}-NTA, with the raw trace, background corrected trace and distance distribution shown left-to-right. Data is shown in black, and the background correction and Tikhonov fit are shown in red.

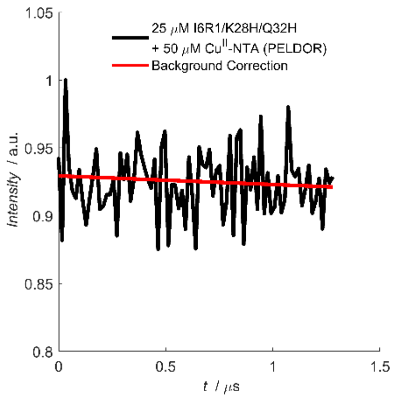

Figure S45: Cu^{II}-Cu^{II} PELDOR measured for 25 µM I6H/N8H/K28H/Q32H GB1 + 50 µM Cu^{II}-NTA. Data is shown in black, and the background correction is shown in red.

Experiment	Zero-time [ns]	Background start [ns]	Background cut-off [ns]	Modulation depths (Δ)
Cu^{II}-nitroxide RIDME	206	324	1232	0.45
Cu^{II}-Cu^{II} RIDME	204	216	1248	0.20
Cu^{II}-Cu^{II} PELDOR	171	320	1280	-

Table S28: A comparison of background correction parameters and modulation depths for traces shown in figures S43-45.

Both Cu^{II}-Cu^{II} PELDOR and Cu^{II}-Cu^{II} RIDME measurements are averaged faster than the Cu^{II}-nitroxide RIDME. For simplicity we consider an average enhancement of the averaging rate by a factor of 20. All factors relevant for determination of the sensitivities per echo (S_e) and per unit time (S_t) are given in table S29.

Experiment	RMS noise	Δ	S_e	Relative S_e	Averaging rate	S_t	Relative S_t
Cu^{II}-Cu^{II} PELDOR	1.38 x 10^{-1}	0.01	2.56 x 10^{-2}	1.00	660	0.658	1.00
Cu^{II}-Cu^{II} RIDME	2.93 x 10^{-2}	0.20	2.41	94.2	660	62.0	94.2
Cu^{II}-nitroxide RIDME	9.26 x 10^{-3}	0.45	17.2	671	33.3	99.1	151

Table S29: Sensitivity for Cu^{II}-Cu^{II} PELDOR and RIDME and Cu^{II}-nitroxide RIDME.

Given that the signal-to-noise (and consequently modulation-to-noise) ratio is proportional to the square root of the number of averages, this translates to a loss in sensitivity for Cu^{II}-nitroxide RIDME due to slower averaging. The overall sensitivity gain for Cu^{II}-nitroxide and Cu^{II}-Cu^{II} RIDME over Cu^{II}-Cu^{II} PELDOR is ~150-fold and ~100-fold, respectively. Directly comparing Cu^{II}-Cu^{II} RIDME and Cu^{II}-Cu^{II} PELDOR there is about a factor of 20 from modulation depth (1% vs. 20%) and another factor of 5 from the RMSD noise.

PELDOR is recorded off-resonance in an over-coupled resonator leading to weaker pulses and detection sensitivity while RIDME is detected in a critically coupled resonator. In combination this will lead to two orders of magnitude (5 × 20 = 100) improvement. It should be appreciated that these values are discussed as a non-exhaustive treatment, but are sufficient to conclude that both Cu^{II}-Cu^{II} and Cu^{II}-nitroxide RIDME experiments are approximately two orders of magnitude more sensitive than Cu^{II}-Cu^{II} PELDOR at Q-band frequencies. However, this will become more favourable for PELDOR when ultra-wideband setups are employed.

2.13 25 µM and 75 µM RIDME Pseudo-Titration Series:

As mentioned in the manuscript, RIDME pseudo-titrations were also performed for 75 µM and 25 µM I6R1/K28H/Q32H GB1 in presence of Cu^{II}-IDA and Cu^{II}-NTA, respectively, and for 25 µM I6H/N8H/K28R1 in presence of Cu^{II}-IDA and Cu^{II}-NTA. Corresponding data is shown in figure S46 below, with K_D estimates given in table S30. Initially these binding isotherms were processed and fitted using a univariate error function where only K_D could vary and Δ_{Tmix} was always 0.5. In this case the hyperbolic function was poorly resolved, and so led to adoption of a bi-variate fitting approach, which also scaled Δ_{Tmix} (this was predicated on the observation that in practice Δ_{Tmix} is closer to 0.45) and this is discussed more in-depth in section 2.14 of the SI.

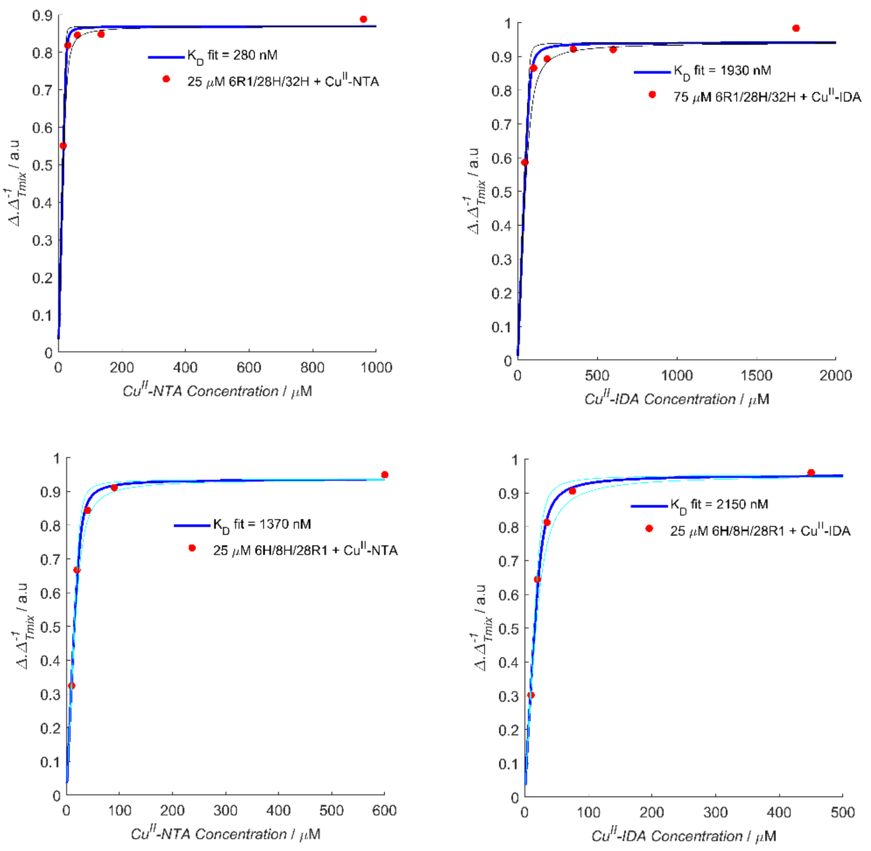

Figure S46: Binding isotherms of the RIDME pseudo-titrations, for 25 µM and 75 µM 6R1/28H/32H GB1 + Cu^{II}-NTA and Cu^{II}-IDA, (top right and left), respectively, and 25 µM 6H/8H/28R1 GB1 + Cu^{II}-NTA and Cu^{II}-IDA (bottom right and left), respectively. K_D values are given in table S30. The cyan traces show isotherms for a K_D a factor 2 higher and lower than the best fit (solid blue line). The black traces show isotherms for a K_D a factor 5 and 4 higher and lower than the best fit, for 6R1/28H/32H in presence of Cu^{II}-NTA and Cu^{II}-IDA, respectively.

Construct-Chelator Permutation	K_D Estimate at 239 K [µM]	K_D Estimate from RIDME pseudo-titrations [µM]
I6R1/K28H/Q32H GB1 + Cu^{II}-NTA	0.22	0.28
I6R1/K28H/Q32H GB1 + Cu^{II}-IDA	2.48	1.93
I6H/N8H/K28R1 GB1 + Cu^{II}-NTA	0.75	1.37
I6H/N8H/K28R1 GB1 + Cu^{II}-IDA	1.73	2.15

Table S30. The estimated K_D values taken from the RIDME binding isotherms given in figure S46, using a bi-variate fitting approach.

It can be seen from the cyan traces that for the 6H/8H/28R1 construct, for the Cu^{II}-IDA and Cu^{II}-NTA pseudo-titration series the fit is sensitive to a factor 2 in the estimated K_D. However, for the other pseudo-titrations, the fit was sensitive to a factor 5 and 4 in K_D by visual inspection for Cu^{II}-NTA and Cu^{II}-IDA, respectively, shown as black traces. This posed the issue of sensitivity to low (particularly sub-µM) K_D as is estimated from the 6R1/28H/32H + Cu^{II}-NTA pseudo-titration series, and so this was the motivation to measure the 500 nM pseudo-titration series with this construct. It should also be noted that the fit value of Δ_{Tmix} ranges from 0.43-0.49, whereas the theoretical limit is 0.5.

A comparison of the ITC-extrapolated and RIDME-determined K_D values is given below in table S31, and it is seen that these differ by less than a factor 2, showing good agreement.

Pseudo-titration Series	Predicted K_D [nM]	Calculated Δ_{Tmix} from Bi-variate Fit
25 µM I6R1/K28H/Q32H GB1 + Cu^{II}-NTA	280	0.434
75 µM I6R1/K28H/Q32H GB1 + Cu^{II}-IDA	1380	0.490
25 µM I6H/N8H/K28R1 GB1 + Cu^{II}-NTA	1370	0.467
25 µM I6H/N8H/K28R1 GB1 + Cu^{II}-IDA	2150	0.476

Table S31: A comparison of the predicted K_D values at 239 K, using van't Hoff's equation and the thermodynamic data from ITC (section 2.6), with K_D values estimated from the RIDME pseudo-titrations shown in figure S46.

This is better visualised in figure S47 below, showing the behaviour of K_Ds with temperature in the range 175-300 K. The black dots indicate the temperature at which the RIDME-determined K_D values match the extrapolated ITC-determined K_D using van't Hoff's equation. For all samples, the temperature is remarkably consistent to a temperature between 230 and 240 K. This is an internal control, since all RIDME pseudo-titration samples are measured with 50% (v/v) deuterated ethylene glycol and aqueous buffer, meaning the temperatures where the dynamics are frozen-out should be similar. Most importantly, this demonstrates there is strong numerical agreement between the RIDME-determined K_D values and the room-temperature ITC-determined values, once one takes account of the different temperature regimes. The precise temperatures and log(K_D) values are given in table S32.

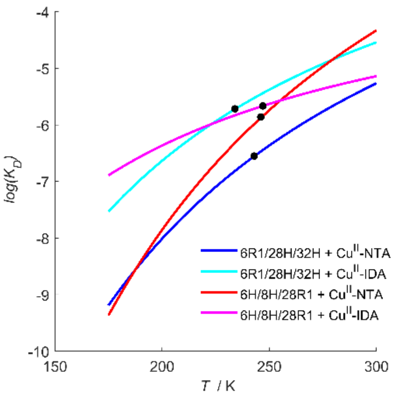

Figure S47: The dependence of K_D on temperature, calculated using van't Hoff's equation and the empirical ΔH values taken from ITC (section 2.6). Here the solid lines show the predicted behaviour of each construct/Cu^{II}-chelate dissociation constant as a function of temperature, and the black scatter indicates the temperature at which the RIDME-determined K_D intersects the predicted affinity from ITC data.

Construct-Chelator Permutation	Temperature [K]	log(K_D)
I6R1/K28H/Q32H GB1 + Cu^{II}-NTA	243	-6.55
I6R1/K28H/Q32H GB1 + Cu^{II}-IDA	234	-5.71
I6H/N8H/K28R1 GB1 + Cu^{II}-NTA	246	-5.86
I6H/N8H/K28R1 GB1 + Cu^{II}-IDA	247	-5.67

Table S32: A comparison of the temperature values where the RIDME-determined K_D intersects with the prediction using the van't Hoff's equation and the thermodynamic data from ITC (section 2.6), with the corresponding log(K_D) values also given.

It is shown in figure S47 that the 75 µM pseudo-titration of 6R1/K28H/Q32H in presence of Cu^{II}-IDA deviates slightly from the trend observed for the other pseudo-titrations. This is reflected by the lower temperature at which the RIDME-determined K_D intersects with the ITC prediction and suggests that the deviation is the result of an over-estimation of the affinity from the RIDME pseudo-titration. This is also reflected by the RMSD of the fit which is approximately a factor 3 greater than for the other series, and so poses an interesting point of discussion regarding error analysis, which is currently being pursued. However, even with this in mind the RIDME- and ITC-determined K_D values have exquisite numerical agreement, suggesting this approach is highly robust.

2.14 The Influence of T_1 and Δ_{Tmix} on K_D Estimation from Modulation Depth Quantitation:

It was necessary to investigate the influence of parameters on the quality of K_D fits and estimation and validate the robustness of the methodology; especially considering that the K_D estimation relies on several parameters, such as T_1, Δ_{Tmix}, and assumes empirical modulation depth can be determined with high accuracy. Initially, the K_D was approximated through minimisation of a uni-variate error function, shown in the top left panel in figure S48; in this case only K_D could vary, with T_1 values being determined empirically for each discrete sample in the pseudo-titration series and Δ_{Tmix} calculated according to eq. 1 in the main text.

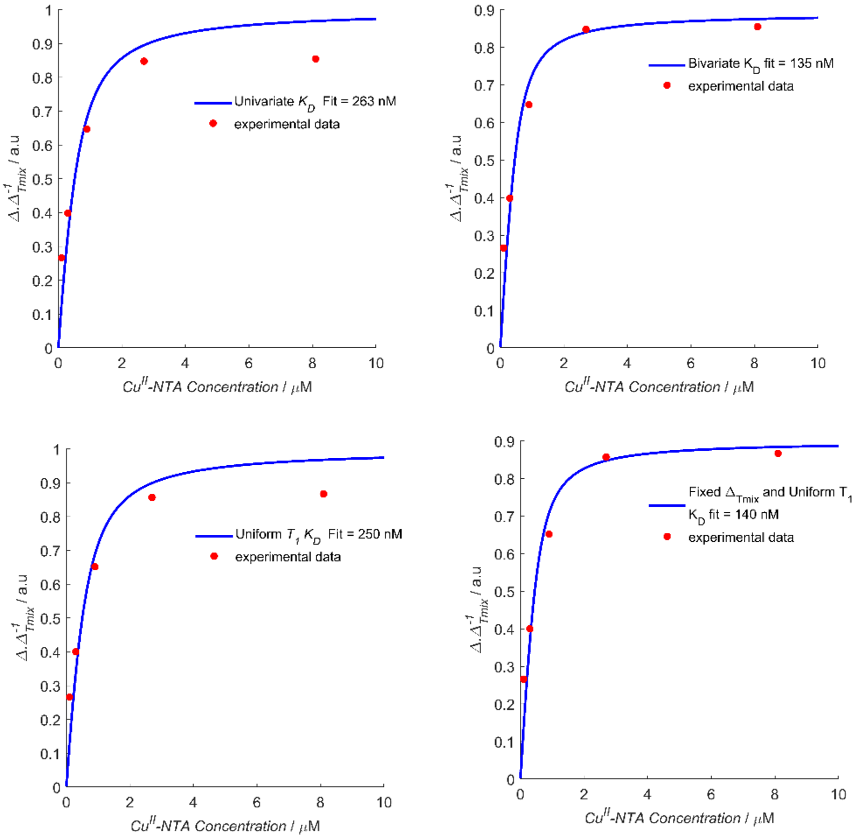

Figure S48. A comparison of the different fits and approaches to approximate K_D from the 0.5 µM I6R1/K28H/Q32H Cu^{II}-NTA pseudo-titration series. The experimental data, and fits are shown as red dots and solid blue, respectively. The top row shows uni-variate and bi-variate fits of K_D, (left and right, respectively) and the bottom row shows uni-variate fit of K_D with a uniform T_1 value, and with a uniform T_1 value and scaling factor for Δ_{Tmix} (left and right, respectively).

Fitting Approach	K_D Approximation [µM]
Uni-variate (Individual T_1 Values)	0.26
Bi-variate	0.14
Uni-variate (Uniform T_1 Value)	0.25
Uni-variate (Uniform T_1 Value and Δ_{Tmix} Scaling Factor)	0.14

Table S33. A comparison of the different K_D values approximated from the 0.5 µM I6R1/K28H/Q32H Cu^{II}-NTA pseudo-titration series by the different fitting approaches, shown in figure S48.

Since it was observed empirically that Δ values tended towards 0.45, as mentioned in the main text, the product $\Delta \times \Delta_{Tmix}{}^{-1}$ tended towards 0.9 rather than 1.0, and therefore Δ_{Tmix} was also estimated simultaneously with K_D using a bi-variate fit; this is shown in the top right panel in figure 48. This improved the fit significantly, hence the decision to scale-down the observed Δ. It was then predicted that the approximated K_D from the bi-variate fit should be the same, if Δ_{Tmix} was fixed to a value of 0.45 instead of 0.5, and then a uni-variate fit was used; this is shown in the bottom right panel in figure S48.

To check the influence of longitudinal relaxation times, a uniform value of T_1, taken from the sample with the lowest ligand concentration, was assumed across the pseudo-titration set, and the K_D approximated by a uni-variate fit; this is shown in the bottom left panel in figure S48. There is little difference between a uni-variate fit, in which each sample in the pseudo-titration has an individual T_1, and that in which a uniform T_1 is assumed. This is likely because the mixing block interval (T_{mix}) is relatively long with respect to T_1, 200 µs compared to ~50 µs; therefore, large relative variations in T_1 may not have a significant impact on the calculated Δ_{Tmix}, and this is particularly true for the hetero-spin case (metal and inorganic radical). The approximated K_D values for all fits vary by less than a factor 2 and are given in table S33.

2.15 Theoretical Extension of Modulation Depth Quantitation to a Two-site Binding Model:

In the case of a single binding-site and single ligand, the saturation function is described by the quadratic given in equation 2 of the main-text. Here, a complete derivation of this equation is given, before expanding modulation depth quantitation to treat a two-site binding model.

$$\Delta \times \Delta_{Tmix}{}^{-1} = \frac{(K_D + [P]_t + [M]_t) - \sqrt{(K_D + [P]_t + [M]_t)^2 - 4[P]_t[M]_t}}{2[P]_t} \qquad (12)$$

Let us first define Δ and Δ_{Tmix} as the experimentally observed and theoretical maximum of Δ for a given ratio of T_{mix} and T_1. We also assume that all protein is quantitatively labelled with nitroxide, and that free nitroxide is quantitatively removed, such that only a protein-bound nitroxide contributes to the detected echo. Each protein is assumed to have a single dH Cu^{II}-binding site and all sites have identical affinity. Consider that when all dH-motifs are empty, Δ = 0 and so the corresponding quotient between Δ and Δ_{Tmix} is also 0. When all dH-motifs are loaded, all nitroxide labels have a Cu^{II}-chelate spin-label partner, and so all are modulated by the dipolar frequency, therefore $\Delta = \Delta_{Tmix}$ and thus the quotient is 1. In all other cases the echo will be the weighted sum of signals from proteins with empty and occupied sites and thus the quotient is directly reporting the fractional saturation of the binding site θ.

For the reaction: P + M ⇋ PM

$$K_D = \frac{[P][M]}{[PM]} = \frac{([P]_t - [PM])([M]_t - [PM])}{[PM]} \qquad (13)$$

Where $[P]_t$ and $[M]_t$ are total protein and metal ion concentrations, respectively, and [PM] is the concentration of protein-metal ion complex.

Then:

$$[PM] \times K_D = ([P]_t - [PM])([M]_t - [PM]) \qquad (14)$$

And:

$$[PM] \times K_D = ([P]_t \times [M]_t) - ([PM] \times [P]_t) - ([M]_t \times [PM]) + [PM]^2 \quad (15)$$

Thus:

$$0 = [PM]^2 - ([P]_t + [M]_t + K_D) \times [PM] + [P]_t \times [M]_t \quad (16)$$

Which is of the form:

$$ax^2 + bx + c = 0$$

Where: $x = [PM]$, $a = 1$, $b = -([P]_t + [M]_t + K_D)$ and $c = [P]_t \times [M]_t$ and therefore:

$$[PM] = \frac{\left(([P]_t + [M]_t + K_D) - \sqrt{([P]_t + [M]_t + K_D)^2 - 4 \times [P]_t \times [M]_t}\right)}{2} \quad (17)$$

Division by total protein ($[P]_t$) yields an expression of the single binding-site saturation function.
Considering the assumptions provided above, one can write the formal expression:

$$\Delta \times \Delta_{Tmix}{}^{-1} = \Theta = \frac{[PM]}{[P]_t} = \frac{(K_D + [P]_t + [M]_t) - \sqrt{(K_D + [P]_t + [M]_t)^2 - 4[P]_t[M]_t}}{2[P]_t} \quad (18)$$

For example in the case of 6H/8H/28H/32H GB1 in presence of Cu^{II}-NTA, choosing $T_{mix}/T_1 = 0.7$ for maximum sensitivity and with the observed modulation depth of 0.18 $\Delta \times \Delta_{Tmix}{}^{-1}$ becomes 0.72. This means 72% of detected Cu^{II} spins are in a protein with two occupied dH sites. Even without the competition by the second site the previous K_D estimate of 285 μM for β-sheet[15c] would only lead to 45% occupation (eq. 2 main-text), suggesting a much stronger affinity of binding.

As mentioned, this approach will tend to an over-estimation of loading, since it does not account for competition between the two-sites, and the influence this has on free metal concentration, therefore a full treatment for a two-site binding model is desirable. The quotient between Δ and Δ_{Tmix} in this case can be expressed as follows:

$$\Delta \times \Delta_{Tmix}{}^{-1} = \frac{[M]_{1,2}}{[M]_t} \quad (19)$$

Let us first define Δ and Δ_{Tmix} as above, where $[M]_t$ is the total Cu^{II}-chelate concentration, and $[M]_{1,2}$ is defined as the concentration of Cu^{II}-chelate intra-molecularly coupled to a second Cu^{II}-chelate spin label in a tetra-histidine construct. In other words, the concentration of spin label in doubly-labelled tetra-histidine protein. Here, we are assuming the detected Cu^{II}-echo is proportional to $[M]_t$, which is not necessarily true for changing speciation or differential rates of T_1 relaxation between free- and bound Cu^{II}-chelate. It is important to emphasise that unlike in the Cu^{II}-nitroxide case, where Δ increases to Δ_{Tmix} in a hyperbolic fashion as a function of increasing ligand concentration, for the tetra-histidine construct Δ is a log-normal function with respect to ligand concentration. Put another way, when excess Cu^{II}-chelate is added (under conditions of full saturation of dH sites) it will contribute to the detected Cu^{II}-echo but will not be intra-molecularly coupled to another spin label, which will reduce Δ overall. Thus, if we again consider a case where there is no dH-loading, the protein will not contribute to the detected echo. In case of free Cu^{II}-label or singly-labelled protein Δ will be 0, as will the quotient between Δ and Δ_{Tmix}. When all Cu^{II}-chelate is bound to dH-sites, (excess free Cu^{II} is negligible), then the quotient is 1. As all Cu^{II}-chelate is either free or bound to dH sites the quotient reflects the ratio of Cu^{II} in doubly-labelled protein to total Cu^{II}.

The definition of $[M]_{1,2}$ is given as:

$$[M]_{1,2} = 2\theta_{1,2}[P]_t \quad (20)$$

Let us first define $[Cu^{II}]_{1,2}$ as above, where $[P]_t$ is defined as the total concentration of tetra-histidine construct and $\theta_{1,2}$ is defined as the fractional saturation for both dH-motifs and will depend on the K_Ds of the sites, and free metal concentration, $[M]$. The factor two accounts for the stoichiometry of 2:1 between dH-bound metal and tetra-histidine protein.
The fractions of protein with zero, one and two metal ions bound can best be defined by the binding polynomial.[19]

$$Z = 1 + (K_1 + K_2)[M] + (K_1K_2K_{12})[M]^2 \quad (21)$$

With the K_i being the association constants and K_{12} describing the cooperativity of binding. In this framework the fraction of unloaded, singly loaded and doubly loaded protein are, respectively

$$\theta_0 = \frac{1}{Z} \tag{22}$$

$$\theta_1 = \frac{(K_1 + K_2)[M]}{Z} \tag{23}$$

$$\theta_{1,2} = \frac{(K_1 K_2 K_{12})[M]^2}{Z} \tag{24}$$

With K_1=1/K_{D_1}, K_2=1/K_{D_2} and K_{12}= 1 the different fractions can be calculated if the free metal concentration is known.[20]

References

[1] T. F. Cunningham, M. R. Putterman, A. Desai, W. S. Horne, S. Saxena, *Angew. Chem. Int. Ed. Engl.* **2015**. *54*, 6330; *Angew. Chem.* **2015.** *127*, 6428.
[2] M. R. Mehlenbacher, F. Bou-Abdallah, X. X. Lin, A. Melman, *Inorganica. Chimica. Acta.* **2015,** *437*, 152.
[3] S. Milikisyants, F. Scarpelli, M. G. Finiguerra, M. Ubbink, M. Huber, *J. Magn. Reson.* **2009.** *201*, 48.
[4] M. Pannier, S. Veit, A. Godt, G. Jeschke, H. W. Spiess, *J. Magn. Reson.* **2000.** *142*, 331.
[5] K. Keller, A. Doll, M. Qi, A. Godt, G. Jeschke, M. Yulikov, *J. Magn. Reson.* **2016.** *272*, 108.
[6] G. Jeschke, V. Chechik, P. Ionita, A. Godt, H. Zimmermann, J. Banham, C. R. Timmel, D. Hilger, H. Jung, *Appl. Magn. Reson.* **2006.** *30*, 473.
[7] S. Rhazzaghi, M. Qi, A. Nalepa, A. Godt, G. Jeschke, A. Savitsky, M. Yulikov, *J. Phys. Chem. Lett.* **2014.** *5*, 3970.
[8] C. D. Schwieters, J. J. Kuszewski, N. Tjandra, G. M. Clore, *J. Magn. Reson*. **2003.** *160*, 65.
[9] M. D. J. Powell, *Comput. J*. **1964.** *7*, 155.
[10] L. Verlet, *Phys. Rev. Lett.* **1967.** *159*, 98.
[11] G. Hagelueken, R. Ward, J. H. Naismith, O. Schiemann, *Appl. Magn. Reson.* **2012**. *42*, 377.
[12] (a) Y. Polyhach, E. Bordignon, G. Jeschke, *Phys. Chem.Chem. Phys.* **2011**, *13*, 2356-2366; (b) G. Jeschke, *Prot. Sci.* **2018**, *27*, 76-85; (c) S. Ghosh, S. Saxena, G. Jeschke, *Appl*. *Magn*. *Reson*. **2018**. *11*, 1281.
[13] J. Gao, F. Xing, Y. Bai, S. Zhu, *Dalton. Trans.* **2014**. *43*, 7964.
[14] N. J. Greenfield, *Nat. Protoc.* **2006.** *1*, 2876.
[15] S. Ghosh, M. J. Lawless, G. S. Rule, S. Saxena, *J. Magn. Reson.* **2018.** *286*, 163.
[16] (a) S. Valera, K. Ackermann, C. Pliotas, H. Huang, J. H. Naismith, B. E. Bode, *Chem. Eur. J.* **2016**, 22, 4700; (b) K. Ackermann, C. Pliotas, S. Valera, J. H. Naismith, B. E. Bode, *Biophys. J.* **2017.** *113*, 1968.
[17] G. Jeschke, Y. Polyhach, *Phys. Chem. Chem. Phys.* **2007**, *9*, 1895.
[18] S. Rebsdat, D. Mayer in *Ullmann's Encyclopedia of Industrial Chemistry*, Wiley-VCH, **2000**, DOI: 10.1002/14356007.a10_101
[19] J. J. Wyman, *Adv. Protein Chem.* **1964**, *19*, 223–286; (b) D. F. Senear, M. Brenowitz, *J. Biol. Chem.* **1991**, *266*, 13661–13671.
[20] Z. X. Wang, R. F. Jiang, *FEBS Lett.* **1996**, *392*, 245–249.

Author Contributions

JLW and KA produced GB1 constructs, JLW, KA and AJS performed their biophysical characterisation, AG and BEB developed EPR methodology, JLW and BEB performed the EPR experiments, JLW, DGN and BEB modelled data. All authors contributed to data analysis, validation and discussion. DGN and BEB devised the project, JLW and BEB wrote the manuscript with input from all authors.
